# *In silico* mapping of non-canonical DNA structures across the human ribosomal DNA locus

**DOI:** 10.1093/g3journal/jkaf299

**Published:** 2025-12-10

**Authors:** Jyoti D Adala, Bruce A Knutson

**Affiliations:** Department of Biochemistry and Molecular Biology, State University of New York Upstate Medical University, Syracuse, NY 13210, United States; Department of Biochemistry and Molecular Biology, State University of New York Upstate Medical University, Syracuse, NY 13210, United States

**Keywords:** ribosomal DNA (rDNA), ribosomal RNA (rRNA), RNA polymerase I (Pol I), non-canonical DNA structures (NCS), R-loops, G-quadruplexes (G4s), i-motifs (iMs), GC content, transcriptional regulation, evolutionary conservation

## Abstract

Ribosomal DNA (rDNA) encodes the precursor transcripts for ribosomal RNAs (rRNAs), which are processed into the structural and catalytic components of the ribosome, making them indispensable for protein synthesis and cell viability. Uniquely, the transcribed human rDNA locus is exceptionally GC-rich, a feature that promotes the formation of non-canonical DNA structures (NCS) such as R-loops, G-quadruplexes (G4s), and i-motifs (iMs). While previous studies have reported NCS in specific regions of human rDNA, there is no comprehensive map of their distribution across the entire human rDNA sequence. Here, we use validated computational tools to systematically identify predicted NCS sequences (PNCSS) across the human rDNA locus. Our analyses reveal that R-loop-, G4-, and iM-forming sequences are non-randomly distributed in the rDNA. These PNCSS are enriched in non-coding spacer regions, including 5′ external transcriber spacer (5′ETS), internal transcriber spacers (ITS1 and ITS2), and the 3′ETS. PNCSS are also enriched in specific subdomains of the 28S coding region, while they are strikingly depleted from the 18S region. These motifs exhibit strong strand asymmetry, frequent co-localization, and evolutionarily conserved enrichment across vertebrate species. Notably, regions enriched for PNCSS are inversely correlated with RNA polymerase I (Pol I) occupancy, suggesting these structures might impede transcription and serve regulatory or quality control functions. Together, our findings define a coherent and conserved non-canonical structure architecture within the human rDNA locus. These PNCSS represent genomic hotspots for structural elements that regulate rDNA biology and represent targetable features for therapeutic intervention.

## Introduction

The genomic organization of ribosomal DNA (rDNA) is highly conserved across eukaryotes, reflecting its essential role in ribosome biogenesis and cellular growth. In humans, rDNA genes are organized as tandem repeats located within nucleolar organizing regions on the short arms of acrocentric chromosomes (Tschochner and Hurt 2003; Moss et al. 2007; Baßler and Hurt 2019). Each rDNA repeat consists of a transcribed unit and an intergenic spacer (IGS), which border transcribed units (Smirnov et al. 2021). The transcribed unit comprises sequences encoding the 18S, 5.8S, and 28S rRNAs, which are separated by internal transcribed spacers (ITS1 and ITS2) and flanked by external transcribed spacers (5′ ETS and 3′ ETS) (Sharifi and Bierhoff 2018). Transcription by RNA Polymerase I (Pol I) produces a long precursor 47S rRNA that is processed into mature 18S, 5.8S, and 28S rRNAs that are integral structural and catalytic components of the ribosome (Kuhn et al. 2007; Schneider 2012; Scull and Schneider 2019; Vanden Broeck and Klinge 2024). Tight regulation of rDNA transcription is essential, as its dysregulation has been implicated in various pathologies including cancer, neurodegeneration, and developmental disorders, such as Treacher Collins syndrome (Dixon et al. 2007; Trainor et al. 2009; Hannan et al. 2013; Hallgren et al. 2014; Sulima et al. 2019; Warmerdam and Wolthuis 2019; Kampen et al. 2020; Bursac et al. 2021; Pitts and Laiho 2022; Zhou et al. 2025).

A striking feature of the rDNA transcription unit is the unusually high guanine-cytosine (GC) content, generally above 70% and reaching up to ∼90% in certain regions (Symonova 2019; Fan et al. 2022). The rDNA locus GC content far exceeds the average GC content of the human genome (∼41%) (Piovesan et al. 2019) as well as other functional genomic regions, including coding exons (50% to 55%; Kalari et al. 2006; Amit et al. 2012), introns (35% to 45%); (Qiu et al. 2024), and untranslated regions (UTRs; typically ∼35% to 60%; Zhang et al. 2004). Although GC-richness is known to influence gene regulation elsewhere in the genome in terms of regulating gene expression (Amit et al. 2012; Qiu et al. 2024) the significance of rDNA's extreme GC content remains largely underexplored.

Compared to the classical Watson and Crick B form DNA, GC-rich sequences are prone to forming alternative stable structures known as non-canonical DNA structures (NCS) (Smirnov et al. 2023). Notable examples of NCS include R-loops, G-quadruplexes (G4s), and i-motifs (iMs). R-loops are three-stranded nucleic acid structures that form co-transcriptionally when a nascent RNA transcript hybridizes with its complementary DNA strand, displacing the non-template strand as single-stranded DNA (Thomas et al. 1976; Drolet et al. 1995; Aguilera and García-Muse 2012; Santos-Pereira and Aguilera 2015). R-loops often accumulate in regulatory genomic regions such as CpG islands, gene termini, and immunoglobulin switch regions (Roy et al. 2008; Roy and Lieber 2009; Ginno et al. 2013; Wahba et al. 2013; Chen et al. 2017; Niehrs and Luke 2020; Xu et al. 2023). In yeast, R-loops are especially enriched within rDNA, where their persistence impedes RNA polymerase I (Pol I) transcription, particularly in the absence of topoisomerase I (Top1) (El Hage et al. 2010). Supporting this, studies in human cells have also identified clusters of Pol I-associated R-loops across the rDNA locus (Malig et al. 2020).

G-quadruplexes (G4s) are stable secondary structures that arise in guanine-rich DNA when the strand becomes single-stranded, allowing guanine bases to interact via Hoogsteen hydrogen bonding (Sen and Gilbert 1988; Hanakahi et al. 1999). G4s form planar G-tetrads that stack into multilayered structures stabilized by monovalent cations such as potassium (Sen and Gilbert 1988; Duquette et al. 2004; Maizels 2006; Spiegel et al. 2020; Varshney et al. 2020). G4s are enriched at telomeres, promoters, and replication origins, and are implicated in transcriptional regulation, replication stress, and genomic instability (Eddy and Maizels 2006; Hansel-Hertsch et al. 2016; Huppert and Balasubramanian 2005, 2007; Shen et al. 2021). Recent studies have identified G4s within the rDNA of both plants and humans, where they are hypothesized to modulate rDNA transcription (Hansel-Hertsch et al. 2016; Havlová and Fajkus 2020; Datta et al. 2021). In addition, several therapeutic strategies now aim to stabilize rDNA G4s to disrupt ribosome biogenesis in cancer cells (Mergny and Hélène 1998; Drygin et al. 2011; Hansel-Hertsch et al. 2017; Musso et al. 2018; Sanchez-Martin et al. 2021). G4s frequently co-occur with R-loops and directly promote each other's formation (Lyu et al. 2022; Wulfridge and Sarma 2024). During R-loop formation, the displaced guanine-rich single-stranded DNA often folds into a G4, resulting in a coupled G4-R-loop structure referred to as a G-loop (Duquette et al. 2004; Maizels and Gray 2013; Kuznetsov et al. 2018; Lee et al. 2020). The potential interplay between R-loop and G4 and their co-occurrence is largely unknown in the rDNA locus.

Complementing G4s are cytosine-rich DNA structures, known as iMs, which arise from intercalated cytosine pairs (Gehring et al. 1993; Manzini et al. 1994). While iMs form independently, they frequently arise on the cytosine-rich strand opposite to G4s, reflecting the strand complementary inherent to GC-rich sequences (Abou Assi et al. 2018; Tao et al. 2024; Deep et al. 2025). Although early studies suggested that iMs form only under non-physiological acidic conditions, more recent work has demonstrated their formation in living cells including within the nucleolus, the site of Pol I transcription and the rDNA locus (Wright et al. 2017; Zeraati et al. 2018). In the genome, iMs are enriched at gene promoters and regulate transcription of key oncogenes (eg *MYC, KRAS, BCL2*) (Brooks et al. 2010; Lian et al. 2012; Kendrick et al. 2014; Saha et al. 2020). Despite individual reports of R-loops, G4s, and iMs in rDNA across various species (El Hage et al. 2010; Zeraati et al. 2018; Mestre-Fos et al. 2019; Havlová and Fajkus 2020), their distribution and potential regulatory roles in the human rDNA locus remain largely unexplored (Chmurciakova et al. 2022; Smirnov et al. 2023). This knowledge gap stems in part from the highly repetitive and GC-rich nature of rDNA, which complicates standard sequence alignment, genome assembly, and epigenomic profiling. As a result, rDNA is frequently underrepresented or excluded from genome-wide datasets, limiting our understanding of the rDNA structural and functional landscape.

We hypothesize that the human rDNA locus harbors hotspots for the formation of non-canonical DNA structures (NCS) due to its high GC content, repetitive architecture, and robust transcriptional activity. To test this, we applied multiple computational approaches to systematically identify and map sequence elements predicted to form R-loops, G4s, and iMs across the rDNA repeat unit. Our analyses revealed that predicted non-canonical DNA structure sequences (PNCSS) are unevenly distributed and exhibit evolutionary conservation. Notably, we found that sequences with a high propensity to form R-loops and G4s show an inverse correlation with Pol I occupancy. Together, these findings suggest that NCS represent underappreciated features of the rDNA landscape that may influence Pol I transcription and rRNA processing in human cells.

## Materials and methods

### Reference rDNA sequence

To perform a comprehensive sequence-level analysis of the human rDNA locus, we first reconstructed a complete reference rDNA unit. A search of the NCBI database (Sayers et al. 2025) identified 2 entries: U13369.1 (Gonzalez and Sylvester 1995) (https://www.ncbi.nlm.nih.gov/nuccore/555853) and KY962518.1 (Kim et al. 2018) (https://www.ncbi.nlm.nih.gov/nuccore/KY962518.1). U13369.1 contains undefined nucleotides in the IGS region; therefore, we used the more recent and complete KY962518.1, which is 44,838 nucleotides (nt) in length. To generate a full rDNA unit for analysis, we added ∼2,000 nt of promoter sequence from the IGS to the upstream of 5′ETS. The resulting reconstructed rDNA unit includes the promoter, 5′ETS, 18S, ITS1, 5.8S, ITS2, 28S, 3′ETS, and IGS and was used in all downstream analyses.

### Prediction of non-canonical DNA structures sequences

R-loop forming sequence (RLFS): RLFS were identified using QmRLFS-Finder (Wongsurawat et al. 2012; Jenjaroenpun et al. 2015, 2017), which applies a quantitative model of R-loop architecture. The algorithm searches for 3 elements: an R-loop initiation zone (RIZ; requires ≥2 guanine clusters and ≥50% G content), a linker zone (0 to 50 nt of any nucleotide composition, providing flexibility), and an R-loop elongation zone (REZ; 100 to 2,000 nt with ≥40% G content, required for stable propagation). The program outputs genomic coordinates, sequence, and strand information. RLFSs were further classified into RIZ subtypes m1 (G_3_N_1-10_G_3_N_1-10_G_3_) and m2 (G_4_N_1-10_G_4_).

Deep learning-enhance R-loop prediction tool (DeepER): Nucleotide base level R-loop prediction was performed using deep learning tool, DeepER (Hu et al. 2024). DeepER tool employs a bidirectional LSTM architecture trained on high throughput sequencing R-loop mapping datasets. For each nucleotide, DeepER outputs a probability of the nucleotide's participation in forming R-loop structure. Predictions were generated using Script.py, as recommended by the authors.

G-quadruplex forming sequence (G4FS): The human rDNA locus sequence was scanned for G4FS (Huppert and Balasubramanian 2005; Todd et al. 2005; Lombardi and Londoño-Vallejo 2020). This identifies the classical intramolecular G4 motif with 4 G-tracts separated by short loops using the pattern G_3+_N_1-7_ G_3+_N_1-7_ G_3+_N_1-7_ G_3+_. Here, G_3+_ denotes a run of ≥3 guanines, while N_1-7_ denotes loop of any nucleotides of length 1 to 7 (Papp et al. 2023). The program output genomic coordinates, sequence, and strand in a tab-separated file.

i-motif forming sequence (iMFS): C-rich motifs predicted to form iMs were identified with iM-Seeker using end-to-end model with the default setting (Yu et al. 2024). The search motif C_3-10_N_1-12_C_3-10_N_1-12_C_3-10_N_1-12_C_3-10_, requires 4 cytosine tracts (3 to 10 nt) separated by flexible loops (1 to 12 nt). The tool outputs genomic coordinates and sequences of all potential iMFS in a comma-separated file.

### Visualization of rDNA

We used karyoploteR (v1.30.0) (Gel and Serra 2017) in R (v4.4.1) to visualize the human rDNA locus. A custom karyotype was generated with the rDNA locus represented as a single artificial chromosome using the *plotKaryotype* function. Structural annotations were displayed with *kpRect*, which draws colored rectangles to demarcate annotated rDNA regions, each assigned a distinct color for clarity.

Predicted sequence features were visualized with 2 complementary functions *kpPlotRegions and kpPlotCoverage*. *kpPlotRegions* highlights each predicted motif interval as a discrete block along the sequence (eg an interval spanning positions 1 to 10 and another spanning 2 to 15 are shown as 2 separate blocks, regardless of overlap). In contrast, *kpPlotCoverage* aggregates overlapping intervals into coverage profiles, where peaks height is proportional to the number of overlapping sequences (eg RLFS, G4FS, or iMFS) sharing the same nucleotide. This approach was applied to plot coverage of RLFS, G4FS, and iMFS across the rDNA locus.

### Curation, regional assignment, and quantification of predicted non-canonical DNA structure sequences

PNCSS (eg RLFS, G4FS, iMFS) were imported from tab or comma-delimited files containing genomic coordinates, sequence, name, and strand into R using data.table (v1.17.6) (Barrett et al. 2024) and tidyverse (v2.0.0) (Wickham et al. 2019) packages. Each PNCSS category was then assigned to an rDNA subregion in a strand-specific manner based on its start position. Features were summarized per region to obtain PNCSS category counts; density (PNCSS counts normalized by region length); and PNCSS category proportion (PNCSS divided by total PNCSS in that category). Visualization was generated in ggplot2 (v3.5.2) (Wickham 2016), with density on the *y*-axis and region on the *x*-axis. A consistent color palette was used across all figures. Summary tables (region, length, count, density, and percentage contribution) were exported as CSV for reproducibility and serve as inputs for subsequent analysis.

### Nucleotide composition and strand asymmetry

The human rDNA locus sequence was imported in FASTA format into R using Biostrings (v2.72.1) (Pagès et al. 2024). Base composition (A, T, G, C) was calculated as both raw counts and percentages. GC content was computed as (G + C)/(total bases) using 100 bp sliding window to capture local variation. GC skew was calculated as (G − C)/(G + C), where positive values indicate G-rich and negative values indicate C-rich regions. Plots of nucleotide composition, GC content, and GC skew were generated in ggplot2 (v3.5.2) (Wickham 2016).

### RNA polymerase I (Pol I) coverage summarization

Preprocessed BigWig (bw) files for RNA Pol I chromatin immunoprecipitation sequencing (ChIP-seq) were obtained from the public GitHub repository of Paralkar et al. (George et al. 2023) (https://github.com/vikramparalkar/rDNA-Mapping-Genomes). To summarize signal intensity across the rDNA locus, we divided the human rDNA locus reference sequence into 100 consecutive, non-overlapping intervals (bins) using a custom BED file. Coverage values were extracted with deepTools multiBigwigSummary (Ramirez et al. 2016) for Pol I. For each interval (bin) defined in the custom BED file, the tool calculated the mean signal intensity and saved a tab-delimited output table. The tabular output was imported into R for downstream analysis. Signal values for each track were subsequently rescaled to a 0 to 1 range (min–max normalization) to facilitate comparison of Pol I with RLFS, G4FS, and iMFS.

### Integration of *RNA* Pol I Ch*IP* signals with PNCSS

We used the custom BED created in previous step that contains 100 non-overlapping bin coordinates for the human rDNA locus reference sequence. RLFS, G4FS, and iMFS datasets were assigned to bins by counting motif start sites within each interval. For each dataset, values were rescaled to the range 0 to 1 (min–max normalization) to enable comparability across tracks. These normalized values were plotted together with Pol I on dual *y* axes (Pol I on the left; predicted non-canonical DNA structure sequence counts on the right). To assess relationships quantitatively, we performed 2 complementary analyses: bin-wise directionality and Pearson correlation. In bin-wise directionality, for each adjacent bin, we calculated the difference (Δ) in normalized values relative to the previous bin and recorded whether ChIP signal for Pol I and count of each predicted NCS changed in the same or opposite direction. Pearson correlation coefficients were also calculated between Pol I occupancy and each predicted structure's normalized counts across all bins, with significance assessed using 2-tailed tests. This approach allowed us to visualize local anti-correlated patterns (line plots) and quantify global inverse relationships (scatterplots with regression fits).

### Evolutionary conservation

We analyzed rDNA transcription units (5′ETS–3′ETS) from *Macaca mulatta* (Rhesus macaque, KX061890) (Agrawal and Ganley 2016) (https://www.ncbi.nlm.nih.gov/nuccore/KX061890), *Mus musculus* (Mouse, BK000964) (Grozdanov et al. 2003) (https://www.ncbi.nlm.nih.gov/nuccore/BK000964.3), and *Gallus gallus* (Chicken, KT445934) (Dyomin et al. 2016) (https://www.ncbi.nlm.nih.gov/nuccore/KT445934). Pairwise global alignment was performed by aligning these sequences to the human rDNA locus (KY962518.1) (Kim et al. 2018) (https://www.ncbi.nlm.nih.gov/nuccore/KY962518.1) using Biostrings (v2.72.1) pwalign::pairAlignment (Pagès et al. 2024) with parameters match = 2, mismatch = −3, gap opening = −5, and gap extension = −2, yielding pairwise percent identities across full units and individual regions. PNCSS were generated in each species using the same tools as described above in visualization of rDNA method section.

### Extraction of rDNA sequences from human rDNA repeats

Full-length rDNA sequence from 5 human chromosomes 13, 14, 15, 21, and 22 were obtained from the T2T-CHM13v2.0 genome assembly. These sequences were analyzed for PNCSS. All PNCSS output BED files were converted to bedGraph format using standard bedtools (v2.31.1) utilities (Quinlan and Hall 2010). The resulting bedGraph were uploaded to the UCSC Genome Browser for custom track visualization and figure generation (Perez et al. 2025).

## Results

### Human rDNA exhibits region-specific GC enrichment and strand asymmetry

**Fig. 1.**
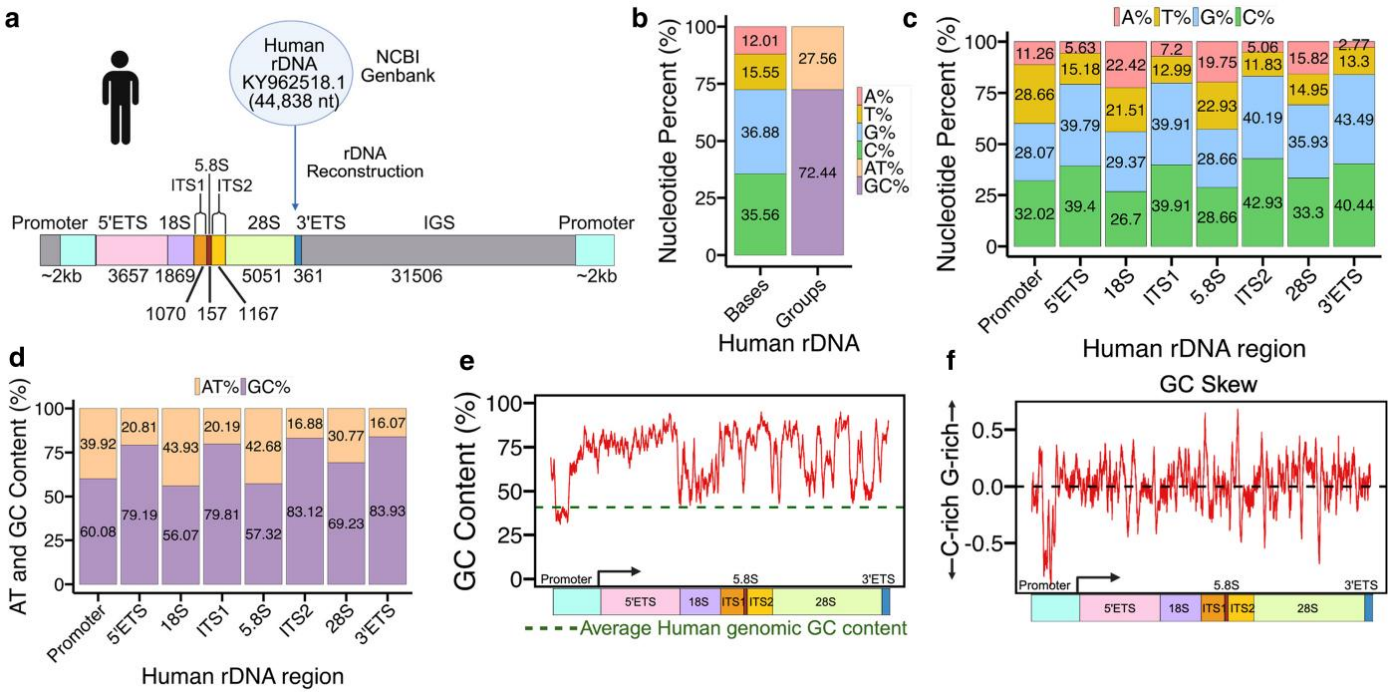
Nucleotide composition and GC Landscape of Human rDNA locus. a) Human rDNA sequence KY962518.1 was selected and extended with ∼2 kb of promoter sequence from the IGS upstream of the 5′ETS to generate a complete reference used for downstream analysis. Each region within rDNA is color-coded, with region name is indicated above and nucleotide lengths shown below. b) Represents the overall nucleotide composition, as well as AT and GC content, of the human rDNA transcriptional unit. c) Percentages of A, T, G, and C across annotated rDNA regions. G and C nucleotides are consistently more abundant than A and T. d) GC vs AT composition for each region within rDNA, showing higher GC enrichment in 5′ETS, ITS1, ITS2, and 3′ETS, and 28S regions. e) GC content calculated in 100-bp sliding windows across the locus. The *x*-axis shows a schematic representation of the human rDNA locus. f) GC skew across the rDNA locus in 100-bp window (*x* axis), with *y* axis scaled from 0.5 to -0.5, highlighting alternating G-rich and C-rich domains.

To investigate the potential for NCS formation within the human rDNA locus, we first reconstructed a complete reference rDNA unit. We used the full-length human rDNA sequence KY962518.1 (44,838 nucleotides, nt) (Kim et al. 2018) from the NCBI database as the backbone. We also included ∼2 kb upstream promoter sequence from the IGS to retain known regulatory elements upstream of the 5′ external transcribed spacer (5′ ETS) (Fig. 1a). We refer to this reconstructed sequence as the human rDNA locus throughout our analysis. Consistent with prior reports, the transcriptional unit (5′ ETS to 3′ ETS), which includes the coding regions for 18S, 5.8S, and 28S rRNAs as well as non-coding spacers (ITS1 and ITS2), was globally GC-rich, with a GC content of 72.44% compared to 27.56% AT content (Fig. 1b; Fan et al. 2022).

We hypothesized that the human rDNA locus contains localized sequence features that favor NCS formation due to its high transcriptional activity and extreme GC enrichment. Because the locus is composed of both coding and spacer regions, with coding regions being critical for pre-ribosome complex formation, we anticipated that these sequence features would not be evenly distributed but instead cluster in discrete functional domains. To test this, we calculated the nucleotide composition across both coding and non-coding regions of the rDNA locus. We found that all regions were enriched in guanine (G) and cytosine (C) relative to adenine (A) and thymine (T) but the degree of enrichment varied by region (Fig. 1c, Supplementary Table 1). Coding regions such as 18S and 5.8S displayed moderate G (29.37 and 28.66%) and C content (26.7 and 28.66%), while 28S contained higher levels with 35.93% of G and 33.3% of C. In contrast, spacer regions showed significantly higher G or C content, ranging from 39.4% to 43.49% (Fig. 1c).

To further explore these differences in base composition, we next calculated AT vs GC content across each region of the transcriptional unit to determine how base composition is distributed (Fig. 1d, Supplementary Table 1). Spacer regions, particularly the 5′ and 3′ ETS and ITS1/2, displayed the highest GC content ranging from 79.19% to 83.93%, followed by the 28S (69.23%), while 18S and 5.8S exhibited comparatively lower GC levels 56.07% and 57.32%, respectively (Fig. 1d). These findings reveal that GC content is not uniformly distributed in the rDNA locus, and that spacer regions, rather than coding regions, are the most GC-enriched (Fig. 1d). This is particularly notable as non-coding regions in the human genome, such as introns, intergenic regions, and UTRs, are typically AT-rich unlike those of the rDNA spacer regions.

Extending beyond global trends, we next examined local GC variation, as GC fluctuations may help define distinct rDNA regions where we found dynamic profile within rDNA regions. For example, GC content rose from ∼40% in the IGS/promoter to ∼90% in the 5′ ETS, declined sharply to in the 18S, increased in ITS1, dipped again in 5.8S, and rose again in ITS2 (Fig. 1e). The 28S region displayed a pattern of sharp GC peaks and valleys, indicative of internal compositional heterogeneity, while GC content remained elevated through the 3′ ETS (Fig. 1e). The non-linear profile of GC content of the rDNA locus suggests functional domain boundaries and possible hotspots for secondary structure formation.

Given the high and variable GC content, we next assessed GC skew to understand the strand asymmetry distribution of G and C nucleotides as a proxy for potential strand-biased structural features (Fig. 1f). GC skew identifies strand-biased guanine enrichment (positive skew) or cytosine enrichment (negative skew). These skew patterns are predictive of NCS such as R-loops and G4s that form in G-rich regions (positive skew) and i-motifs (iMs) that form in C-rich regions (negative skew) (Fig. 1f). Our analysis revealed distinct and localized peaks of both positive and negative GC skew across the locus (Fig. 1f), indicating that rDNA contains discrete G-rich and C-rich sequences. Taken together, these data reveal that the human rDNA locus is not only globally GC-rich but also displays region-specific base composition and localized GC heterogeneity, features that create a strong propensity to form different types of NCS depending on sequence context.

### Predicted R-loop sequences hotspots are enriched in spacer and 28S regions of human rDNA

**Fig. 2.**
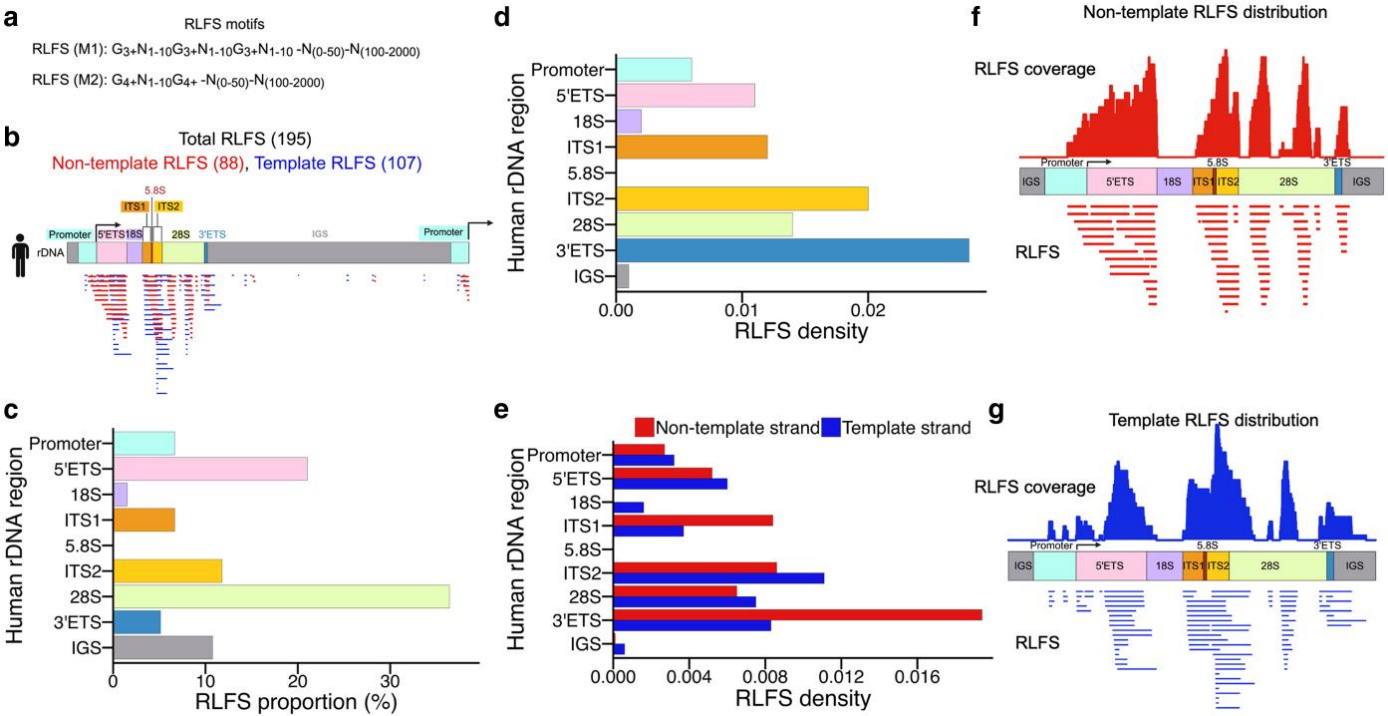
Strand- and region-specific mapping of RLFS in the human rDNA locus. a) RLFS motif search representation. b) Schematic overview showing the distribution of 195 RLFSs across the human rDNA locus, with RLFS clusters of varying length detected on both non-template (red) and template (blue) strands, spanning all spacer regions and within the 28S gene but absent in 18S. c and d) Quantification of RLFSs in the human rDNA locus. The *Y* axis lists rDNA regions, with the *X* axis representing RLFS proportion (c) and RLFS density (d) normalized to region length. e) Strand-specific counts by rDNA region, with red and blue bars denoting non-template and template strands, respectively. f and g) RLFSs coverage plots across the rDNA locus, with peaks reflecting nucleotides shared by multiple overlapping RLFSs on the non-template (f) and template (g) strands. The top panel shows the strand-specific coverage signals, and the lower panel displays the individual RLFS.

Considering the elevated GC content, localized compositional heterogeneity, and strand asymmetry across the rDNA locus, we next asked whether these features correlate with predicted R-loop formation. R-loop structures are enriched at GC-rich loci and have been reported in rDNA across various species, including yeast (El Hage et al. 2010; Ginno et al. 2013). To assess the potential of the human rDNA locus sequence to form R-loops, we applied the Quantitative model for R-Loop Forming Sequence (QmRLFS) Finder algorithm. QmRLFS-finder is a validated bioinformatic tool that predicts R-loop forming sequence (RLFS) based on a defined sequence architecture in a natural or artificial DNA sequence (Fig. 2a) (Wongsurawat et al. 2012; Jenjaroenpun et al. 2015, 2017) (http://r-loop.org/). The model identifies 3 components: (1) an R-loop initiation zone (RIZ), a linker, and an R-loop elongation zone (REZ) on the non-template DNA (Fig. 2a). The RIZ requires clusters of guanines, at least 2 (M1) or 3 (M2) together with ≥50% G content (Fig. 2a). The REZ, a 100 to 2,000 nt sequence with ≥40% G content that facilitates R-loop propagation (Fig. 2a). The linker is a flexible region of 0 to 50 nt with any nucleotide composition that connects RIZ and REZ (Fig. 2a).

We selected QmRLFS finder for primary RLFS annotation in rDNA locus because it is a sequence-based tool that remains widely adopted for R-loop analysis across diverse genomes. Recent studies incorporate QmRLFS predictions in their analysis or benchmarking frameworks (Lin et al. 2022; Vanhaeren et al. 2025). Among newer approaches, deep learning-based R-loop prediction tools, such as DeepER (Hu et al. 2024), have gained popularity due to their high predictive accuracy. To complement QmRLFS predictions, we evaluated DeepER, which outputs a probability for each nucleotide's involvement in R-loop formation. DeepER revealed a series of sharp, high-probability peaks across similar regions in rDNA that were also identified by QmRLFS (Supplementary Fig. 1a and b). These peaks were strand-specific and closely aligned with QmRLFS, supporting the underlying sequence propensity for stable RNA–DNA hybrid formation (Supplementary Fig. 1a and b). Consistently, QmRLFS and DeepER R-loop predictions converge on similar hotspots such as promoter, 5′ETS, ITS1, ITS2, 28S, and 3′ETS, suggesting that these methods better capture the intrinsic sequence propensity for R-loop formation in rDNA.

Utilizing QmRLFS model, we identified 195 RLFSs across the rDNA locus: 88 RLFSs were predicted on the non-template strand (red) and 107 on the template strand (blue) (Fig. 2b, Supplementary Table 2). These RLFSs were non-uniformly distributed across the promoter region through the 3′ ETS and often clustered in specific domains. Several RLFSs originated from the same genomic coordinates but varied in length, suggesting the presence of a hotspot with overlapping R-loop potential (Fig. 2b). Notably, the 18S and 5.8S regions contained few or no RLFSs and multiple gaps were evident within the 28S region (Fig. 2b). To quantitatively compare RLFS distribution, we assigned RLFSs to annotated rDNA regions based on their point of origin, regardless of whether they extended into adjacent regions. The 28S rRNA region contained the largest number of RLFSs (71 out of 195) followed by order 5′ETS (41) > ITS2 (23) (Fig. 2c, Table 1 RLFS). Identical numbers were observed in the promoter and ITS1(13 each), followed by IGS (21), 3′ETS (10), 18S (3), and finally the 5.8S region, which contained none (0) (Fig. 2c, Table 1 RLFS). Because the rDNA regions differ in length, we normalized RLFS density to region length. This analysis revealed that all regions except 5.8S harbored at least one RLFS (Fig. 2d). Spacer regions consistently exhibited the highest RLFS densities and ranked 3′ ETS > ITS2 > ITS1 > 5′ ETS > IGS (Fig. 2d). Among coding regions, 28S exhibited the highest RLFS density, comparable to ITS1 and 5′ ETS, while 18S had the lowest (Fig. 2d).

**Table 1.** Distribution of predicted non-canonical DNA structure sequences (PNCSS) across the human rDNA locus.

PNCSS	RLFS			G4FS			iMFS		
	Total	Non-template	Template	Total	Non-template	Template	Total	Non-template	Template
Human rDNA repeat	195	88	107	210	60	150	85	57	28
Promoter	13	6	7	12	0	12	5	5	0
5′ETS	41	19	22	23	6	17	7	7	0
18S	3	0	3	0	0	0	1	1	0
ITS1	13	9	4	4	4	0	3	3	0
5.8S	0	0	0	0	0	0	0	0	0
ITS2	23	10	13	17	5	12	7	7	1
28S	71	33	38	76	21	55	15	15	0
3'ETS	10	7	3	5	4	1	3	2	0
IGS	21	4	17	73	20	53	44	17	27

Because the RLFS algorithm relies on the orientation of transcription, we examined the strand-specific distribution of RLFSs on the template and non-template strands. RLFSs were found on both template and non-template strands in most regions (Fig. 2e, Table 1 RLFS). Interestingly, all RLFSs in the 18S region were exclusively on the template strand (Fig. 2e, blue), while the ITS1 and 3′ ETS regions were strongly biased toward the non-template strand (Fig. 2e, red). Except for 5.8S and 18S, all other regions showed RLFSs on both strands, highlighting a strand- and region-specific distribution of R-loop potential within the rDNA locus (Fig. 2e). We then generated nucleotide-resolution RLFS coverage plots to visualize local enrichment patterns. In these plots, peaks indicate the number of overlapping RLFSs at each nucleotide position, with higher peaks reflecting stronger potential hotspots for R-loops (Fig. 2f and g). On the non-template strand, RLFSs initiated near the promoter and extended across the 5′ ETS but were absent from the 18S region (Fig. 2f). RLFSs reappeared in ITS1 and ITS2 and exhibited distinct valleys and peaks, resembling a claw-like periodicity within segments of the 28S, consistent with the underlying GC landscape. RLFSs continued through the 3′ ETS, forming dense peaks (Fig. 2f). On the template strand, RLFSs coverage also began near the promoter and extended through the 5′ETS into the 5′ region of 18S, while the remainder of 18S was completely devoid of RLFS (Fig. 2g). These RLFSs reappeared in ITS1 and extended into the 5′ region of 28S (Fig. 2g). Within 28S, RLFSs were present but displayed a distinct positional pattern, with alternating peaks and gaps before and reappearing again in 3′ETS (Fig. 2g).

These results indicate that both strands are prone to R-loop formation, but in region-specific and strand-biased manners. Collectively, our analyses demonstrate that the human rDNA locus harbors sequence-intrinsic potential for R-loop formation that is both non-uniformly distributed and strand-dependent, with a strong preference for spacer regions, suggesting a role in regulating rDNA transcription or pre-rRNA processing.

### Potential G-quadruplex hotspots parallel predicted R-loop sequence distribution in human rDNA

**Fig. 3.**
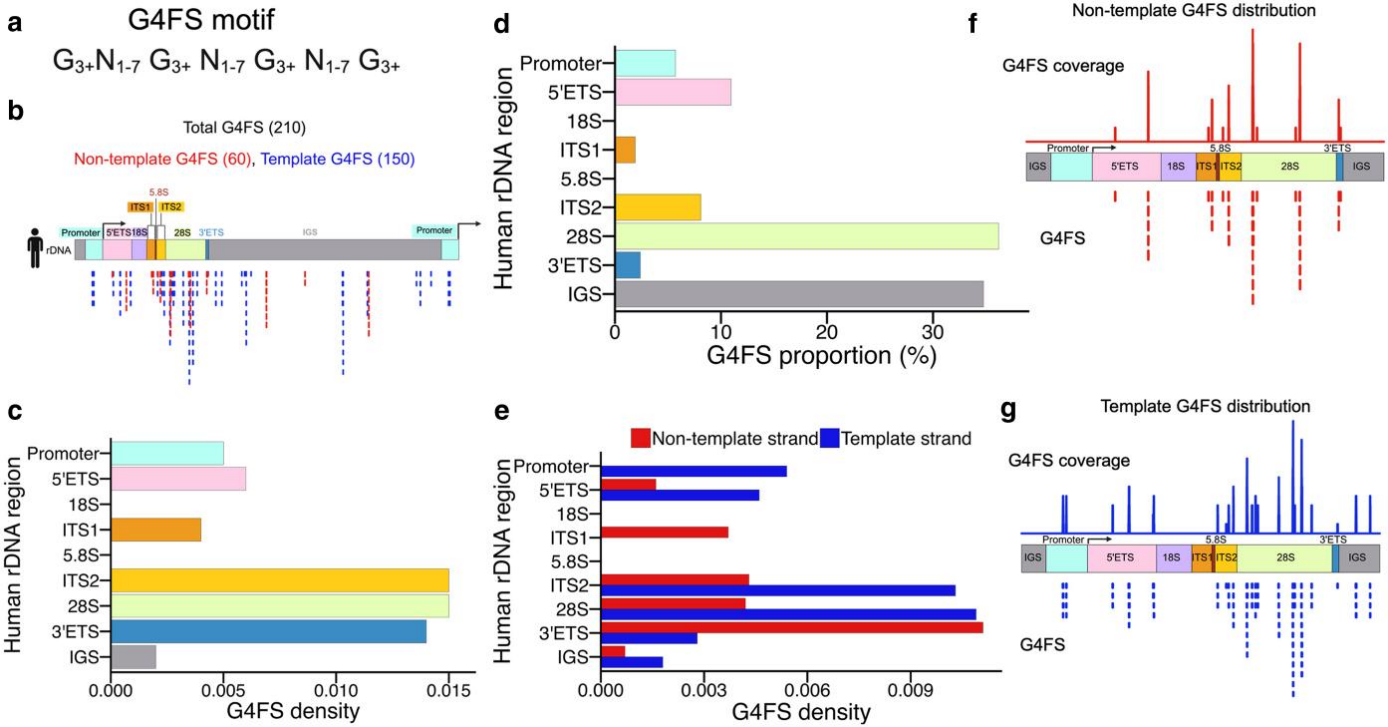
Strand- and region-specific mapping of potential G-quadruplex forming sequence (G4FS) in the human rDNA. a) G4FS motif search representation. b) Schematic shows the location of 210 G4FSs across the human rDNA sequence, with red and blue lines representing non-template and template strands, respectively. Clusters were observed in all spacer regions and the 28S coding region in a strand-specific manner. c and d) Quantification by region is shown as proportions of total G4FSs (c) and as density normalized to region length (d). e) Strand-specific G4FSs counts are shown above each bar, where red and blue bars denote non-template and template strands, respectively. f and g) Coverage plots for G4FSs across the rDNA locus are presented for the non-template (f) and template (g) strands. The top panel shows the strand-specific coverage signals, and the lower panel displays the individual G4FSs.

Given the extreme GC content of the rDNA and the strand asymmetry of RLFSs, we hypothesized that guanine-rich sequences could form G4 motifs in a region-specific manner, similar to R-loops. To test this, we scanned the reconstructed rDNA sequence for G-quadruplex forming sequence (G4FS), using a widely accepted regular expression pattern (Fig. 3a) (Huppert and Balasubramanian 2005; Todd et al. 2005; Lombardi and Londoño-Vallejo 2020; Papp et al. 2023). This pattern (G_3+_N_1-7_G_3+_N_1-7_G_3+_N_1-7_G_3+_), defines runs of ≥3 consecutive guanines (G_3+_), separated by loops 1 to 7 nucleotides (N_1-7_) and identifies high-confidence canonical G4 motifs capable of forming stable 4-stranded structures under physiological conditions (Fig. 3a) (Chambers et al. 2015; Marsico et al. 2019). Although other non-canonical G4 configurations exist, such as bulge G4s (Mukundan and Phan 2013; Piazza et al. 2015; Papp et al. 2023), our analysis focused on G4 canonical form, which is the most broadly validated in genome-wide studies (Marsico et al. 2019). Using this approach, we identified 210 G4FS across the human rDNA locus, 60 on the non-template strand (red) and 150 on the template strand (blue) (Fig. 3b, Supplementary Table 3). Like RLFSs, G4FSs originating from same origin form distinct clusters, predominantly in spacer regions (5′ ETS, ITS1/2, 3′ ETS, and IGS) and in selective regions within the 28S (Fig. 3b). When quantified based on their region of origin, the 28S region contained the largest number of G4FSs (76 out of 210) (Fig. 3c, Table 1 G4FS). The next most enriched G4FSs regions were IGS (73), 5′ETS (23), ITS2 (17), promoter (12), 3′ETS (5), and ITS1 (4), while none were detected in the 18S or 5.8S regions (Fig. 3c, Table 1 G4FS). After normalizing by region length, the highest G4FSs densities were found in the 28S, followed by spacer regions in the order ITS2 > 3′ ETS > 5′ ETS > IGS (Fig. 3d).

Next, we assessed strand-specific G4FSs distributions. On the template strand, G4FS were most abundant in 28S, followed by ITS2, promoter, 5′ ETS, 3′ ETS, and IGS (Fig. 3e, Table 1 G4FS). In contrast, G4FS were absent from 18S, 5.8S, and ITS1 (Fig. 3e, Table 1 G4FS). On the non-template strand, G4FSs were most enriched in the 3′ ETS, followed by ITS2, 28S, ITS1, 5′ ETS, and the IGS but were absent from the promoter, 18S, and 5.8S (Fig. 3e, Table 1 G4FS). Although G4FSs often mirrored the distribution of RLFSs, they were less abundant overall in some regions. For example, compared to RLFSs counts within the same region, non-template G4FSs were reduced in the promoter, 5′ ETS, ITS regions, 3′ ETS, and 28S but were elevated in the IGS (Fig. 3e, Table 1 G4FS). On the template strand, G4FSs were more abundant in the promoter and IGS but were reduced in 5′ ETS, ITS1, and 3′ ETS (Fig. 3f, Table 1 G4FS).

Local strand-specific enrichment can indicate a higher potential to form G4s; therefore, we generated G4FSs coverage plots to assess the rDNA region-specific formation of these NCS. On the non-template strand, sharp peaks indicating greater overlap of G4FSs were evident in spacer regions and within regions of 28S but absent from the promoter, 18S, and 5.8S, indicating highly localized G4-forming hotspots (Fig. 3f). On the template strand, we observed more overlapping G4FSs signals, with intense peaks in the promoter, 5′ETS, ITS2, and 3′ETS (Fig. 3g). Among coding regions, we only see G4FS peaks within regions of 28S (Fig. 3g). Overall, our results demonstrate that G4FSs are non-randomly distributed across the human rDNA locus, with a strong preference for spacer elements and portions of the 28S region, mirroring but not completely overlapping with R-loop potential.

### Strand-specific distribution of predicted i-motif forming sequences in human rDNA

**Fig. 4.**
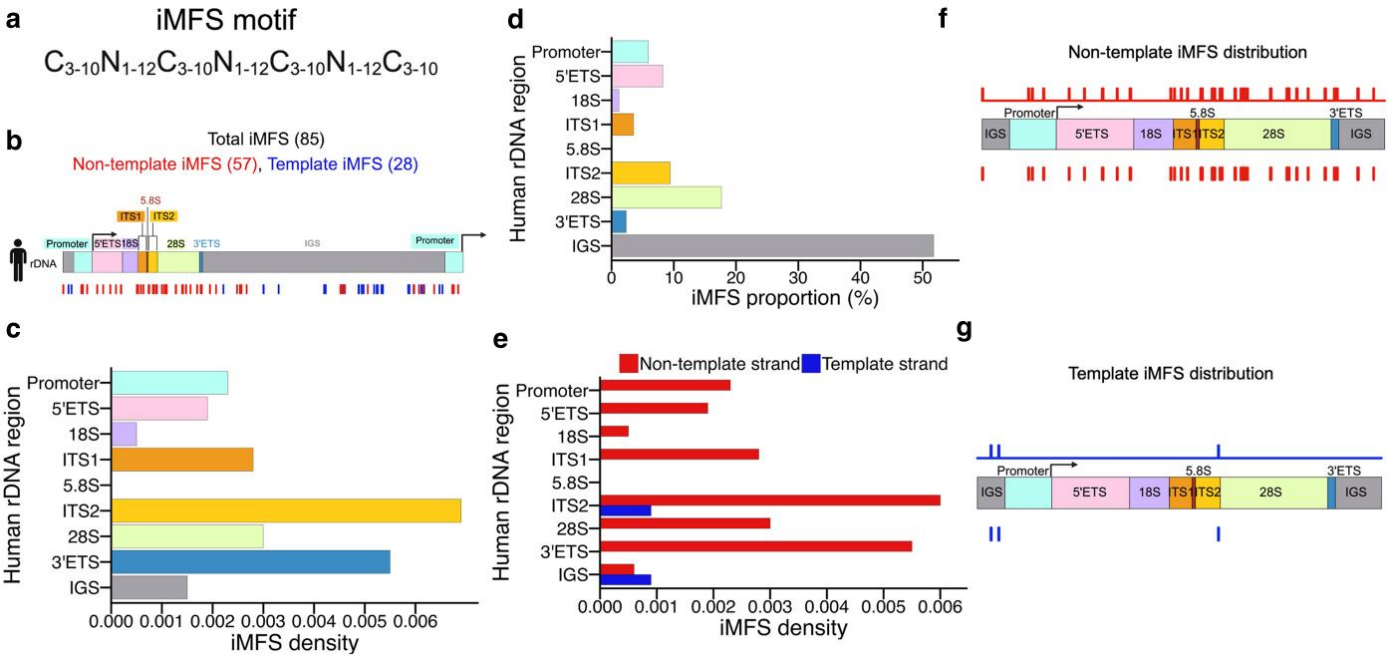
Strand-biased distribution of predicted i-Motif forming sequence (iMFS) in the human rDNA locus. a) iMFS motif search representation. b) Schematic showing the location of 85 iMFSs across the human rDNA sequence, with red and blue lines representing non-template and template strands, respectively. c and d) Quantification by region as proportions of total iMFSs (c) and as density normalized to region length (d). e) Strand-specific counts by region, with red and blue bars denoting non-template and template strands, respectively. f and g) Coverage plots of iMFSs across the rDNA locus on the non-template (f) and template (g) strands. The top panel shows the strand-specific coverage signals, and the lower panel displays the individual iMFS.

Regions with negative GC skew in our earlier analysis (Fig. 1f) correspond to C-rich tracts within the locus, providing sequence contexts favorable for i-Motif (iM) formation. iMs typically occurs on the strand complementary to G4s and forms in cytosine-rich sequences under physiological conditions (Tao et al. 2024). While less studied than R-loops or G4s, iMs have been implicated in transcriptional regulation at promoters and in other repetitive genomic regions (Abou Assi et al. 2018; Luo et al. 2023). To complete our analysis of non-canonical DNA structures in rDNA, we assessed the potential for iM formation. We used iM-Seeker, a computational tool designed to identify i-motif forming sequence (iMFS) (Yang et al. 2024). iM-seeker scans for C_3-10_N_1-12_C_3-10_N_1-12_C_3-10_N_1-12_C_3-10_ motifs, which form 4 cytosine tracts (each 3 to 10 nucleotides) separated by loop sequences of 1 to 12 nucleotides (Fig. 4a) (Yang et al. 2024). Applying this motif to the human rDNA locus, we identified 85 iMFSs, including 57 on the non-template strand and 28 on the template strand (Fig. 4b, Supplementary Table 4). Unlike RLFSs and G4FSs, iMFSs did not show strong clustering or overlap (Fig. 4b). This lack of clustering suggests that iMs are not merely mirror images of G4s but instead iMFS can form independently of G4FS. When quantified by region of origin, all regions except 5.8S contained at least one iMFS (Fig. 4c, Table 1 iMFS). Among all rDNA regions, 28S contributes the highest proportion of iMFSs (15 out of 85) followed by IGS (44), ITS2 (7), 5′ETS (7), promoter (5), ITS1 (3), 3′ETS (3), and 18S (1) (Fig. 4c, Table 1 iMFS). After normalizing for region length, spacer regions again exhibited the highest iMFS density, with the order: ITS2 > 3′ ETS > ITS1 > 5′ ETS (Fig. 4d). Among coding regions, 28S remained the most enriched (Fig. 4d). However, overall iMFS counts were much lower than those of RLFS or G4FS (Table 1).

Strand analysis revealed a strong bias toward the non-template strand, which contained iMFSs in all regions except 5.8S (Fig. 4e, Table 1 iMFS). In contrast, template-strand iMFSs were detected only in ITS2 and IGS (Fig. 4e, Table 1 iMFS). This asymmetry was further reflected in strand-specific coverage profiles. On the non-template strand, iMFSs were most abundant in promoter, 5′ETS, ITS1, ITS2, and 3′ ETS, with scattered signals in 28S and a single motif detected at the 3′end of 18S (Fig. 4f). Within 28S, regions of local iMFS enrichment alternated with iM-free zones, echoing patterns seen with RLFSs and G4FSs (Fig. 4f). On the template strand, only ITS2 and IGS showed detectable signal, with no iMFSs observed in 28S, 18S, or the external spacers (Fig. 4g). Although iMFSs were less abundant than RLFSs or G4FSs, they exhibited a clear strand bias and region-specific enrichment, particularly within ITS regions, 3′ ETS, and 28S (Table 1), indicating that i-motif potential is not uniformly distributed but instead constrained by both sequence context and transcriptional orientation, suggesting specialized structural or regulatory roles within selected rDNA subdomains.

### Structural motif co-enrichment across the rDNA locus

Given the shared GC rich consensus sequence similarities between RLFS, G4FS, and iMFS, we examined whether these PNCSS structures may co-occur within the human rDNA locus, rather than forming independently. For example, the RIZ of the RLFS and the G4FS rely on guanine-rich tracts, and in many cases, the RIZ sequence motif resembles the G4FS motif (Kuznetsov et al. 2018). In addition, iMFSs typically form on the complementary strand to G4s, indicating an intrinsic strand-based relationship (Abou Assi et al. 2018). Based on these shared features, we predicted that RIZ (including both M1 and M2 motifs), G4FS, and iMFS can co-occur in specific regions of the rDNA locus. Additionally, we expect that these PNCSS exhibit positive correlations with each other in their frequency and distribution, reflecting their potential for functional cooperation.

**Fig. 5.**
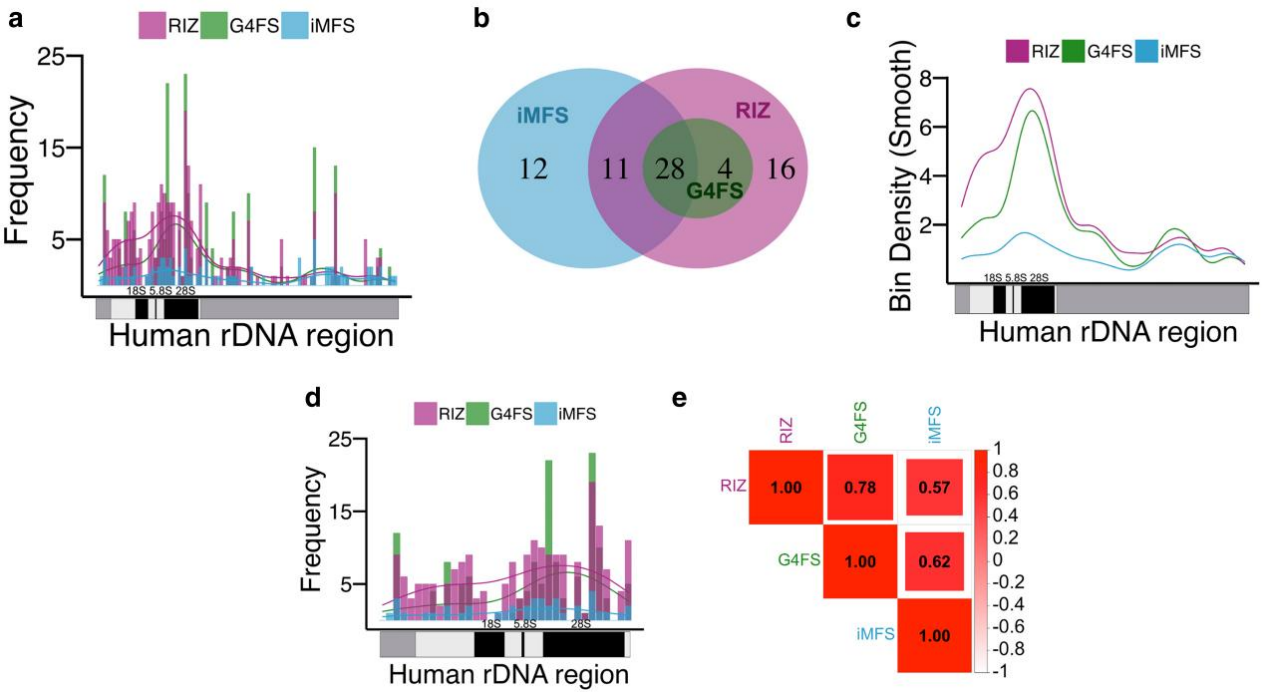
Overlapping distribution of RIZ, G4FS, and iMFS across the human rDNA locus. a) Frequency distribution of R-loop initiation zone (RIZ, pink), G4FS (green), and iMFS (blue) across the rDNA locus divided into 100 bins, with the *x* axis showing the full-length rDNA schematic. Overlapping signals are indicated by mixed bar colors. b) Venn diagram showing the distribution and overlap of RIZ, G4FS, and iMFS across 100 rDNA bins. The largest overlap corresponds to bins containing all 3 structures (28 bins). c) Smoothed bin density plots showing distribution trends of the 3 structures. d) Frequency distribution of non-canonical structure counts across the transcriptional rDNA region. e) Pearson correlation matrix showing correlations between RLFS, G4FS, and iMFS counts across bins.

To assess PNCSS co-enrichment across the rDNA locus, we divided the human rDNA reference sequence into 100 equally sized bins and quantified the frequency of RIZ, G4FS, and iMFS (Fig. 5a, Supplementary Table 5). Notably, we observed several instances where NCS sequence motifs overlapped, suggesting potential co-localization of 2 or more NCS (Fig. 5a and 5b). In addition, we found some NCS that formed independently. For instance, RIZs were most frequently observed independently (16 bins), and iMFS were found in 12 unique bins (Fig. 5b). Pairwise overlaps included 11 bins shared between iMFS and RIZs, 28 between iMFS and G4FS, and 4 between RIZs and G4FS (Fig. 5b). To further assess this relationship, we examined bin density overlays, which reflected both the magnitude and co-enrichment of the 3 PNCSS types across the rDNA (Fig. 5c). Specifically, we observed prominent enrichment within the 5′ ETS, a sharp depletion across the 18S, and renewed clustering in ITS1 and ITS2, with a pronounced peak in the 28S region and sustained signals into the 3′ ETS (Fig. 5d). Among the 3, RIZ and G4FS were the most abundant overall, especially in non-coding spacer regions (Fig. 5d).

To quantify these associations, we performed Pearson correlation analysis between PNCSS classes across all bins. This revealed a moderately strong positive correlation between RIZ and G4FS (*R* = 0.78), followed by G4FS and iMFS (*R* = 0.62), and RIZ and iMFS (*R* = 0.57) (Fig. 5e). These results demonstrate that RIZ, G4FS, and iMFS exhibit co-localization and significant positive correlations across the rDNA locus. The enrichment of all 3 PNCSS classes within spacer regions (ITS and ETS) and 28S coding region suggest that these structural elements may interact or reinforce one another's formation.

### PNCSS profiles across all human rDNA repeats

Given the pronounced PNCSS enrichment in specific regions of the human rDNA reference sequence, we next examined whether similar patterns appear across all rDNA arrays in the human genome. The new human genome assembly T2T CHM13v2.0 (Nurk et al. 2022) contains partial rDNA arrays on 5 chromosomes 13, 14, 15, 21, and 22. To assess how PNCSS features are distributed across the human rDNA landscape, we examined the rDNA-containing regions on all 5 chromosomes using the T2T CHM13v2.0 assembly visualized in the UCSC Genome Browser (Perez et al. 2025). Each of those chromosome harbors a distinct segment of the rDNA array, enabling direct comparison of PNCSS patterns across chromosomes. Despite the fragmented nature of these arrays, we qualitatively observed that all rDNA repeats show similar strand-specific RLFS (Supplementary Fig. 2), G4FS (Supplementary Fig. 3), and iMFS profiles (Supplementary Fig. 4), likely reflecting the high degree of sequence identity shared among rDNA units. Thus, the PNCSS landscape is remarkably uniform across the 5 chromosome rDNA arrays.

### Non-canonical DNA structures are inversely associated with Pol I occupancy

NCS are known to influence transcription by altering DNA topology, impeding polymerase progression, or recruiting regulatory factors. These effects are well established in the context of RNA Polymerase II (Pol II), where R-loops and G4s contribute to promoter-proximal pausing and transcriptional stalling (Chambers et al. 2015; Zardoni et al. 2021). However, whether similar structural features impact Pol I activity within the ribosomal DNA (rDNA) locus remains unclear. To address this, we examined the relationship between predicted NCS-forming sequences and Pol I occupancy across the rDNA repeat.

**Fig. 6.**
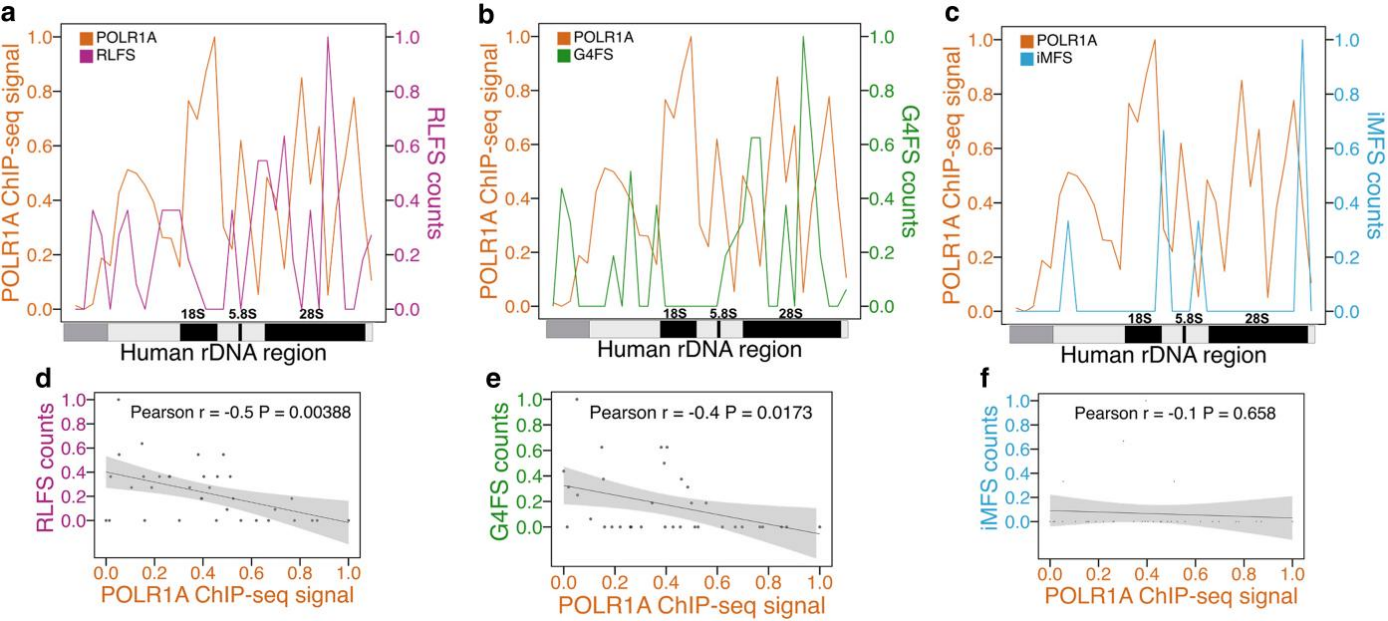
Inverse relationship between POLR1A occupancy and predicted non-canonical DNA structures sequences (PNCSS) in human rDNA. a–c) Publicly available POLR1A ChIP-seq data (orange, George et al. 2023) mapped to the human rDNA transcription unit compared with predicted RLFS (pink, a), G4FS (green, b), and iMFS (blue, c). All signals were scaled from 0 to 1. d–f) Scatterplots with Pearson correlations between POLR1A and RLFS (d), G4FS (e), and iMFS (f).

We hypothesized that regions enriched for PNCSS would exhibit reduced Pol I occupancy. To test this, we analyzed publicly available POLR1A ChIP-seq data. POLR1A encodes the catalytic and largest subunit of Pol I and serves as a widely used proxy for Pol I binding across the rDNA locus (George et al. 2023). We divided the rDNA repeat into 100 equal-length bins and compared POLR1A ChIP-seq signal intensity with the distribution of predicted RLFS, G4FS, iMFS (Fig. 6a–c, Supplementary Table 6). Our analysis revealed an inverse trend between PNCSS density and POLR1A occupancy across the transcribed region. For example, the 18S region, which contains the fewest predicted structures, displayed one of the highest POLR1A ChIP-seq peaks. This pattern supports a potential negative association between NCS presence and Pol I engagement within the rDNA locus.

To more precisely assess the relationship between POLR1A occupancy and PNCSS distribution, we compared changes in signal directionality across 100 bins to determine whether POLR1A and PNCSS signals varied in parallel or inverse patterns. In ∼83% of bins, POLR1A levels changed in the opposite direction to RLFS counts, with similar anti-correlated patterns observed for G4FS (∼86%) and iMFS (∼85%), indicating a consistent inverse trend (Supplementary Table 6).

To quantify these relationships, we performed Pearson correlation analysis. POLR1A signal was significantly negatively correlated with RLFS (*R* = −0.50, *P* < 0.01, Fig. 6d) and G4FS (*R* = −0.40, *P* < 0.05, Fig. 6e), while its correlation with iMFS was weak and not statistically significant (*R* = −0.10, *P* = 0.7, Fig. 6f). Although modest in strength, these negative correlations reinforce the observation that RLFS- and G4FS-enriched regions tend to be less accessible to Pol I.

Taken together, these results demonstrate that Pol I occupancy is inversely associated with PNCSS density, particularly with RLFSs, suggesting that these structures may hinder Pol I recruitment or elongation, similar to what has been observed for Pol II at G-rich or highly structured regions (Szlachta et al. 2018; Shen et al. 2021). The anti-correlation between PNCSS and Pol I occupancy was most prominent in spacer regions, where PNCSS density coincided with reduced Pol I binding. In contrast, PNCSS density was lowest in highly transcribed coding regions such as 18S and portions of 28S. This spatial distribution supports a model in which NCS accumulate in spacer regions while being excluded from core coding regions, potentially acting as regulatory barriers to Pol I activity across the rDNA locus.

### Evolutionary conservation of non-canonical DNA structures distribution at the rDNA locus

**Fig. 7.**
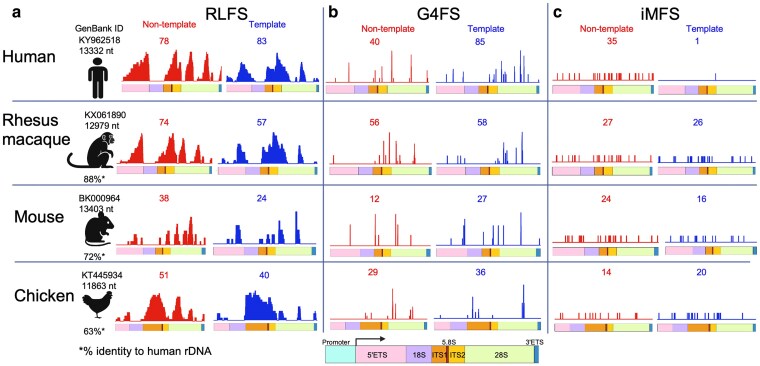
Predicted non-canonical DNA structure sequences (PNCSS) are evolutionarily conserved across rDNA loci of multiple species. a–c) RLFS (a, left), G4FS (b, middle), and iMFS (c, right) across the rDNA transcription units of human (KY962518) (Kim et al. 2018), *Macaca mulatta* (Rhesus macaque, KX061890) (Agrawal and Ganley 2016), *Mus musculus* (Mouse, BK000964) (Grozdanov et al. 2003), and *Gallus gallus* (Chicken, KT445934) (Dyomin et al. 2016). Each panel is divided by strand: non-template (red) and template (blue). Numbers above each plot indicate the total count of predicted structures on that strand. Each panel includes a schematic of the transcription unit, color-coded by rDNA region. The cartoon figure at the bottom shows the full transcription unit with region names, serving as a reference for the color codes used above. Asterisk(*) indicate percent identity of each species' rDNA relative to human.

Although the human rDNA exhibits clear enrichment for RLFSs, G4FSs, and iMFSs, it remains unclear whether these patterns persist across species with divergent rDNA sequences. To address this, we examined PNCSS distribution across multiple vertebrate species to assess whether PNCSS distribution in rDNA is an evolutionarily conserved biological feature. We analyzed rDNA transcription units spanning the 5′ ETS to 3′ ETS from *Macaca mulatta* (Rhesus macaque, KX061890) (Agrawal and Ganley 2016), *Mus musculus* (Mouse, BK000964) (Grozdanov et al. 2003), and *Gallus gallus* (Chicken, KT445934) (Dyomin et al. 2016) and compared to the human rDNA sequence (KY962518) (Kim et al. 2018). These species were selected because their rDNA transcriptional unit are complete and publicly available. As a first step, we evaluated overall sequence conservation by aligning each species' rDNA unit to human and calculating pairwise percent identity. Human rDNA (Fig. 7 Human) shares 88.11% identity with Rhesus macaque (Fig. 7 Rhesus macaque), 71.86% with mouse (Fig. 7 Mouse), and 62.65% with chicken (Fig. 7 Chicken). Among individual regions, the 18S and 5.8S rRNA genes were the most highly conserved (>98% to 99%), whereas spacer regions (5′ ETS, ITS1/2, 3′ ETS) showed substantial divergence (Supplementary Table 7). The 28S gene exhibited moderate to high conservation (∼78% to 94%), with chicken consistently showing the lowest identity across all regions (Supplementary Table 7).

Despite sequence divergence, PNCSS were detectable in all species (Fig. 7). Using the same computational pipelines, we mapped RLFSs (Fig. 7a), G4FSs (Fig. 7b), and iMFSs (Fig. 7c) across each species' rDNA unit. Humans showed the highest total number of PNCSS followed by rhesus macaque, mouse, and chicken, mirroring the hierarchy of sequence conservation (Fig. 7a–c). However, PNCSS were not evenly distributed (Fig. 7). Among the PNCSS, RLFSs were more frequent despite their longer motif requirements, (Fig. 7a, Supplementary Table 8) followed G4FSs (Fig. 7b, Supplementary Table 9) and iMFSs (Fig. 7c, Supplementary Table 10). RLFSs and G4FSs were predicted for both strands but with distinct strand bias (Fig. 7a and b). RLFSs more common on the non-template strand (Fig. 7a), while G4FSs enriched on the template strand in all species (Fig. 7b). For iMFSs, the distribution varies between species (Fig. 7c), in human, rhesus macaque, and mouse rDNA, iMFSs showed a preference for the non-template strand, whereas in chicken, iMFSs were more common on the template strand (Fig. 7c).

All species exhibited enrichment of PNCSS within non-coding spacer regions, including the 5′ ETS, ITS1/2, 3′ ETS and within regions of the 28S (Fig. 7). Although iMFSs were detected within 18S in rhesus macaque, mouse, and chicken, the human 18S region remained largely devoid of PNCSS, suggesting a human-specific constraint (Fig. 7c). Notably, the 28S region consistently contained hotspots for all 3 structural classes, even in chicken rDNA, which is most divergent from human rDNA (Fig. 7a–c). The most striking exception was observed for iMFSs strand bias (Fig. 7c). Humans displayed a pronounced preference for iMFSs formation on the non-template strand, a bias that was not seen in the other species (Fig. 7c). Despite these species-specific features, the presence of NCS in chicken rDNA, with only ∼63% sequence identity, underscores that non-canonical structural potential is conserved beyond strict primary sequence homology (Fig. 7). Together, these findings indicate that the abundance, strand bias, and regional enrichment of PNCSS within the rDNA transcription unit are conserved across vertebrates (Fig. 7). The evolutionary persistence of these patterns supports a model in which NCS contribute to the functional architecture of rDNA across species.

## Discussion

In this study, we provide an analysis of PNCSS across the human rDNA unit that reveals a highly ordered architecture. We observed clusters of RLFSs and G4FSs originating from the same regions, particularly within non-coding spacers and select subdomains of 28S, suggesting that these loci harbor hotspots for R-loop and G4 formation. Predicted R-loops, G4s, and iMs are enriched in non-coding spacers, including the promoter, 5′ETS, internal transcribed spacers (ITS1/2), and 3′ETS, as well as in specific, structurally permissive subdomains of 28S. By contrast, 18S and 5.8S are notably devoid of such structures, with the absence in 5.8S likely due to its small size. These motifs co-localize in strand-asymmetric clusters, align with known GC heterogeneity, and importantly, they show an inverse association with RNA Pol I occupancy. Together, these features suggest that rDNA PNCSS in non-coding spacers and selected 28S regions function as regulatory checkpoints that may influence transcription, processing, and nucleolar organization.

### Preferential accumulation of PNCSS across the rDNA locus

Our computational analysis reveals a striking non-uniform distribution of PNCSS across the human rDNA locus. We observed a strong enrichment of RLFSs, G4FSs, and iMFSs in non-coding spacer regions, particularly the promoter, 5′ external transcribed spacer (5′ ETS), internal transcribed spacers (ITS1 and ITS2), and 3′ ETS. This pattern is consistent with the higher GC content and repetitive nature of these regions, which might predispose them to forming stable non-B DNA structures. In contrast, the 18S rDNA was notably absent from all 3 classes of PNCSS. This scarcity may reflect evolutionary constraints that preserve the sequence and structural integrity of this essential and highly conserved rRNA component, which is integral to the small ribosomal subunit (Lafontaine and Tollervey 2001). The absence of destabilizing secondary structures in 18S may be critical for ensuring high-fidelity transcription and processing of the rRNA precursor. Interestingly, portions of the 28S rDNA exhibited a distinct accumulation of PNCSS, particularly RLFSs and G4FSs, displaying a periodic “claw-like” arrangement. These clusters appear to align with underlying GC-rich sequence patches in the rDNA. While identified as DNA-based NCS, such sequence features could also influence folding of the corresponding 28S rRNA transcript. It is possible that these non-canonical motifs are tolerated, or even functionally relevant, within the larger and more structurally complex 28S rRNA, which contributes to the ribosome's peptidyl transferase center and large subunit architecture (Lafontaine and Tollervey 2001). Taken together, our findings underscore the architectural complexity of the rDNA locus and highlight how non-canonical structure sequence elements are differentially distributed between functional domains.

Interestingly, the accumulation of PNCSS within the 28S rRNA coding region appears to be spatially confined to predicted expansion segments and surface-exposed subdomains. These regions are known to tolerate greater sequence plasticity compared to the highly conserved core functional domains of rRNA. Such structural flexibility may permit the accumulation of PNCSS, including RLFS, G4FS, and iMFS. Indeed, this pattern aligns with population-scale rDNA polymorphism data showing that 18S is highly conserved across individuals, whereas the 28S rRNA harbors disproportionately higher variability within its expansion segments (Fan et al. 2022). The combination of elevated GC content, local sequence heterogeneity, and potential for torsional stress in these regions might provide a permissive substrate for NCS formation. These features reflect a delicate balance between functional tolerance and structural complexity within rDNA, enabling certain regions to accommodate dynamic sequence elements while maintaining overall transcription integrity. Future work should explore whether these predicted structures form in vivo and how their presence affects ribosomal gene expression, nucleolar organization, and cellular homeostasis. In addition, systematic quantification of chromosome-specific variation in PNCSS and its relationship to subtle sequence differences among rDNA units represents an important direction for understanding functional heterogeneity and variability within the rDNA array.

### Interdependence between structural motifs

The strong positive correlation between RLFS and G4FS in the rDNA suggests that these elements occur at similar sites in the rDNA. This is consistent with studies showing that transcription through G-rich DNA generate an RNA:DNA hybrid while the displaced strand folds into a G4 (Duquette et al. 2004; Lee et al. 2020) and with genome-wide maps reporting colocalization of native G4s and R loops (Lyu et al. 2022). Prior work also indicates that G4s and R loops stabilize each other and act together (Ribeiro de Almeida et al. 2018; Wulfridge et al. 2023; Wulfridge and Sarma 2024). In the rDNA, such mutual stabilization could influence promoter-proximal initiation and elongation, and it may also raise genome instability. Stabilizing G4s increases R-loop levels and prolongs single-stranded DNA exposure, leading to DNA damage (De Magis et al. 2019). Given the repetitive nature, high transcriptional activity, and structural complexity of the rDNA locus, such regions are increasingly recognized as hotspots for DNA damage and genome instability. In this context, the interplay between G4s and R loops may not only perturb rRNA transcription but also create persistent lesions that challenge replication and repair pathways, contributing to nucleolar stress and disease-associated phenotypes.

In contrast, we found that iMFS on the complementary C-rich strand were less coordinated with RLFS and G4FS. This is notable, as iMs are often proposed to form opposite G4 structures, yet in the rDNA they appear less aligned. It will be important to test whether this pattern is unique to the rDNA or holds genome-wide. One explanation is sequence-based, since cytosine-rich regions reduce the chance of forming stable R loops and G4s (Bedrat et al. 2016; Cui et al. 2016). A further possibility is that evolutionary pressure has reduced iM stability at regulatory elements to minimize transcriptional interference. Together, these explanations provide a specific rationale for why iMFS are less coordinated with RLFS and G4FS in the rDNA.

### Evolutionary conservation of PNCSS reveals functional significance in rDNA

Across human, rhesus macaque, mouse, and chicken rDNA, we observe a conserved architectural pattern. PNCSS are enriched in rDNA spacer regions, largely excluded from the 18S region, and selectively enriched within variable/expansion subdomains of 28S (Nadel et al. 2015; Abraham et al. 2020). Despite primary sequence divergence in spacers, this organizational pattern is preserved, supporting a conserved role for NCS in modulating rDNA chromatin and co-transcriptional maturation. A major unresolved question is why evolution has preserved such “weak spots” in rDNA, as NCS might physically slow down or stall Pol I. Additionally, we observed a negative correlation between predicted NCS density and Pol I occupancy. One explanation is that these sites function in quality control. For example, R-loops accumulate from the 5′ETS to the 5′ end of 18S but are sparse downstream in wild-type yeast (El Hage et al. 2010). In defective transcription (eg TopI Δ strains), Pol I stalls and aborts early, preventing defective pre-rRNAs from being elongated into the 28S region (El Hage et al. 2010). This “abort early” strategy conserves resources, where pausing at the beginning of 18S is a conserved checkpoint to help RNA folding that are needed for ribosome biogenesis (Schneider et al. 2007; Aguilera and García-Muse 2012).

### Therapeutic opportunities at GC-rich and non-canonical sites

Several clinical agents already exploit GC-rich and G4-prone DNA regions to exert their therapeutic effects (Peltonen et al. 2014; Mitteaux et al. 2021; Jacobs et al. 2022). Among the most well-characterized are the Pol I inhibitors CX-5461 and BMH-21, which bind GC-rich DNA and stabilize G4 structures. Both agents disrupt rRNA synthesis and induce nucleolar stress across a range of cancer types (Drygin et al. 2011; Zimmer et al. 2016; Xu et al. 2017; Ferreira et al. 2020). Broadly, our analysis identified several therapeutically relevant regions in the rDNA locus that include GC-rich spacer regions adjacent to the promoter and 5′ ETS, as well as structurally dynamic domains within the expansion regions of the 28S rRNA gene associated with stress adaptation. These regions exhibit extreme GC content, conformational flexibility, and key regulatory features, making them promising targets for G4-binding compounds, intercalators, and agents that modulate R-loop dynamics (Teng et al. 2021).

Overall, our study defined the first comprehensive, locus-wide map of NCS potential across human rDNA and its vertebrate orthologs. This PNCSS map of the human rDNA potentially highlights regions important for rDNA biology, such as Pol I transcription and/or rRNA processing. Furthermore, this PNCSS map broadens the scope of tractable therapeutic targets within the rDNA landscape and may represent regions that act as vulnerabilities associated with disease onset or progression. Future studies will be essential to validate the functional regions of rDNA and determine how their selective targeting influences rRNA transcription, nucleolar structure, and cell viability.

## Supplementary Material

jkaf299_Supplementary_Data

## Data Availability

All scripts developed for this manuscript are deposited in Github at https://github.com/adalaj/rDNA_project repository. Supplemental material available at G3 online.
