# Supplementary material for: In silico mapping of non-canonical DNA structures across the human ribosomal DNA locus
Source: G3 (Bethesda). 2025 Dec 10;16(2):jkaf299. doi: 10.1093/g3journal/jkaf299 (PMC12869075; doi:10.1093/g3journal/jkaf299)
Supplement: jkaf299_Supplementary_Data [file jkaf299_supplementary_data.zip › Supplemental_Material_G3-2025-406410.pdf]

Supplementary Figure 1

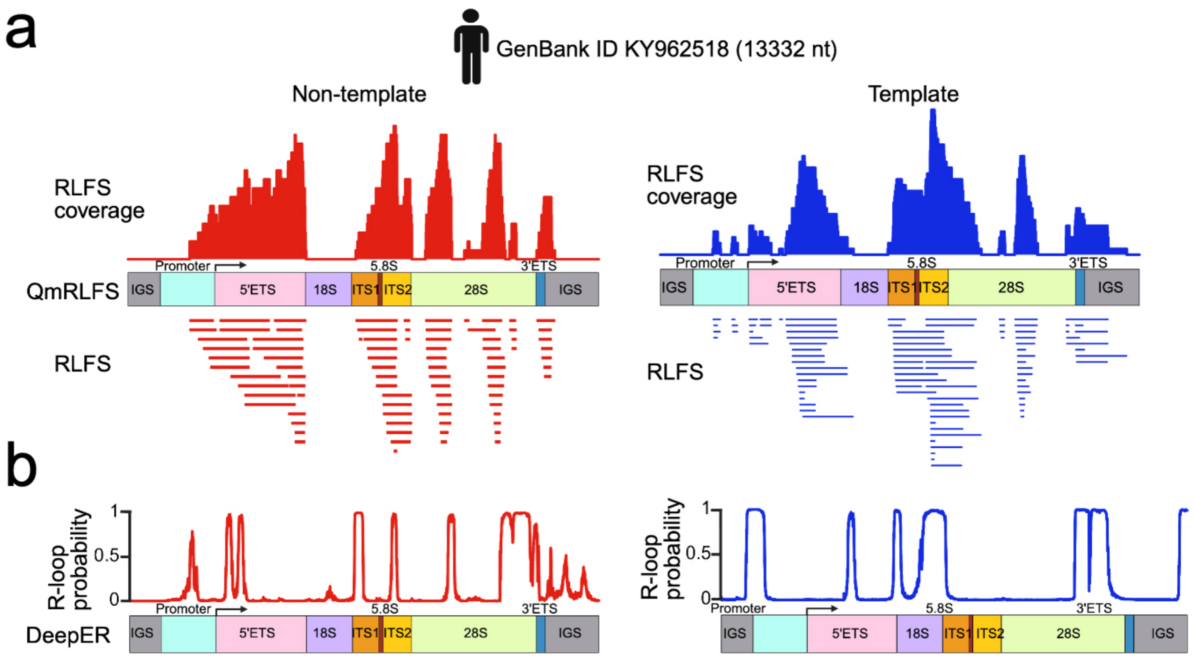

Supplementary Figure 2

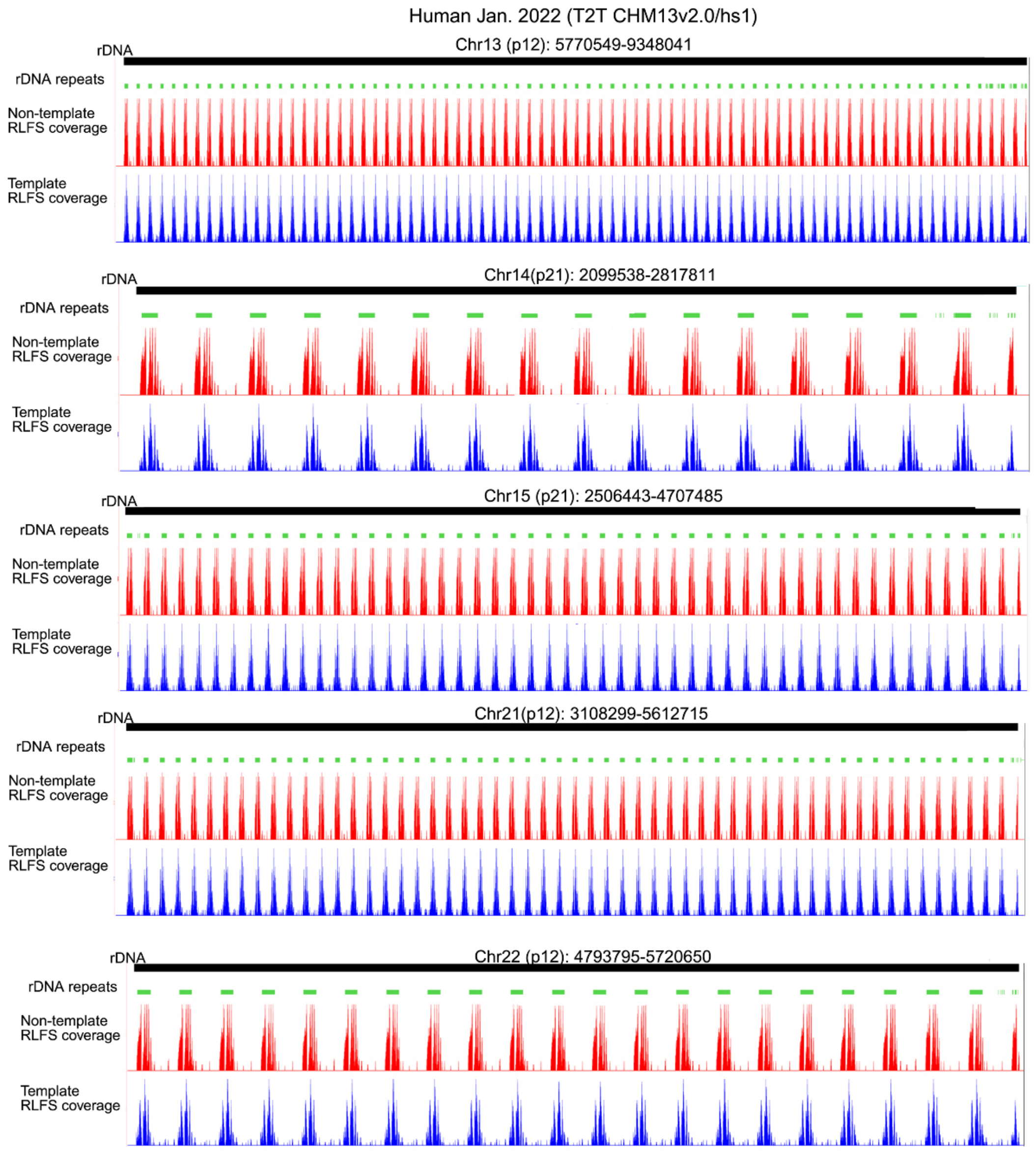

Supplementary Figure 3

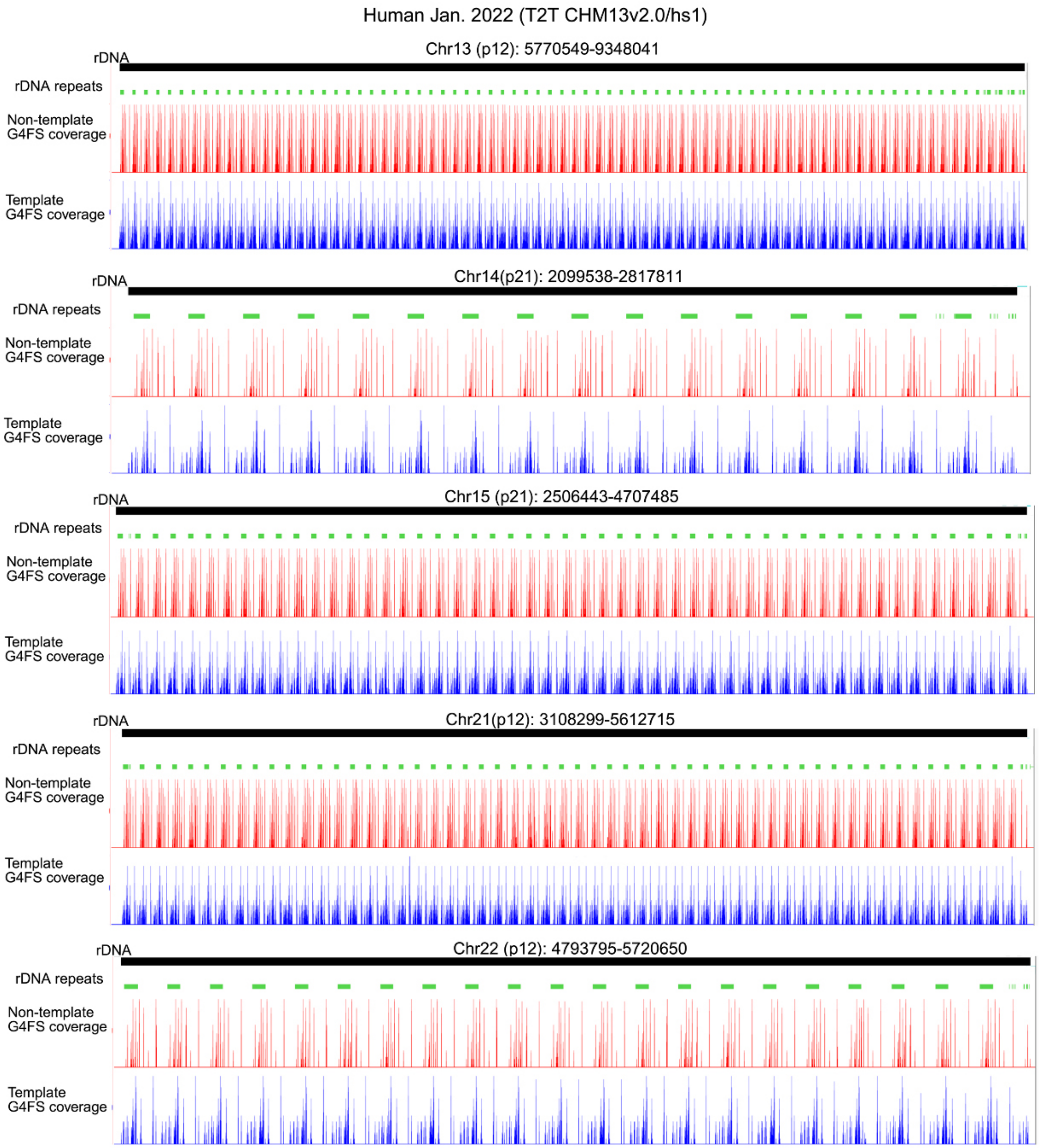

Supplementary Figure 4

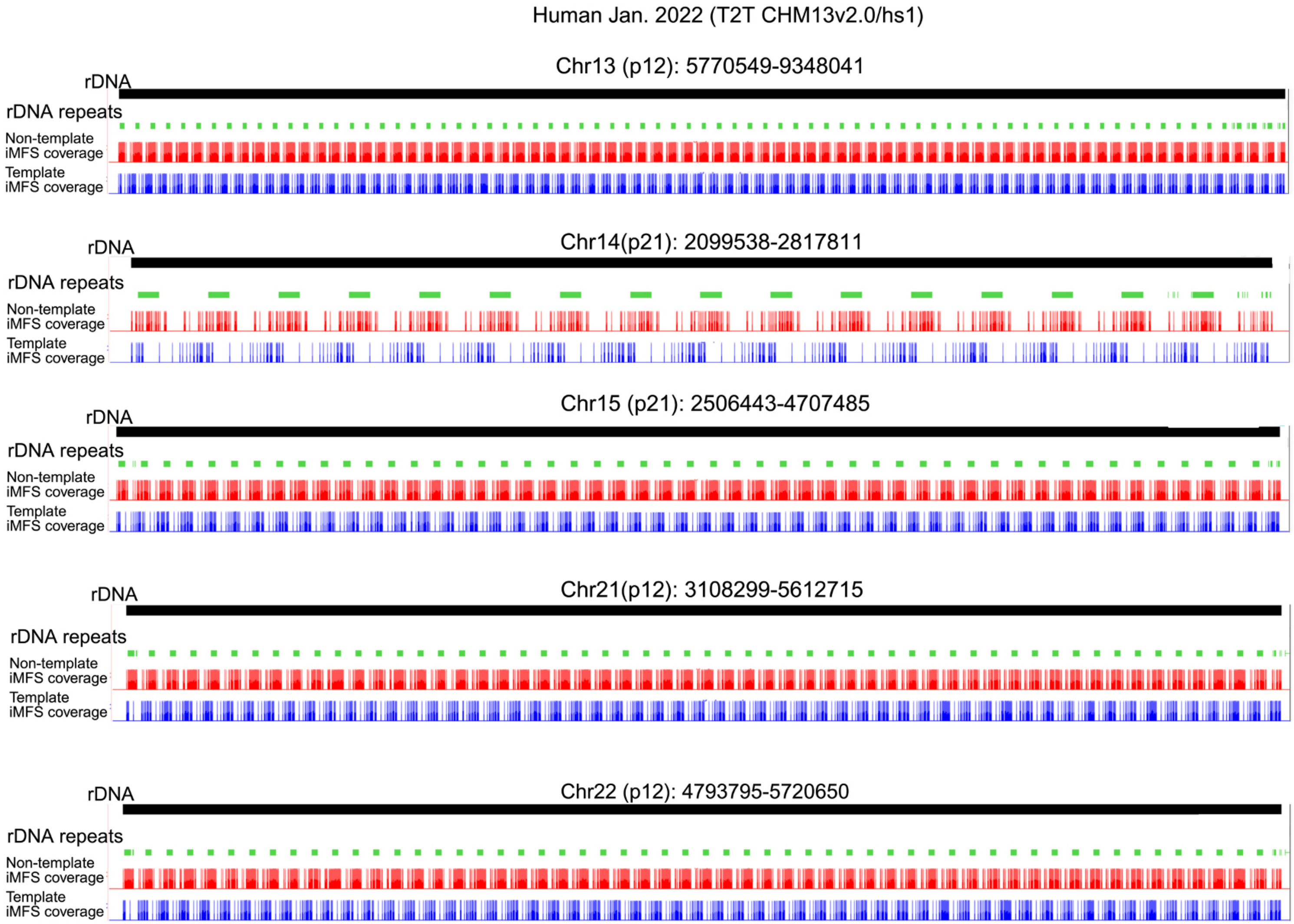

Supplementary Table 1. Characterization of the human rDNA locus KY962518

Name	Details	Total_nucleotides	A	A%	T	T%	G	G%	C	C%	GC_skew_value	x_axis	GC_perc	AT_perc
Promoter_KY962518	1299_3500	2202	248	11.26	631	28.66	618	28.07	705	32.02	-0.065759637	2202	60.08	39.92
5'ETS_KY962518	3501_7157	3657	206	5.63	555	15.18	1455	39.79	1441	39.4	0.004834254	5859	79.19	20.81
18S_KY962518	7158_9026	1869	419	22.42	402	21.51	549	29.37	499	26.7	0.047709924	7728	56.07	43.93
ITS1_KY962518	9027_10096	1070	77	7.2	139	12.99	427	39.91	427	39.91	0	8798	79.81	20.19
5.8S_KY962518	10097_10253	157	31	19.75	36	22.93	45	28.66	45	28.66	0	8955	57.32	42.68
ITS2_KY962518	10254_11420	1167	59	5.06	138	11.83	469	40.19	501	42.93	-0.032989691	10122	83.12	16.88
28S_KY962518	11421_16471	5051	799	15.82	755	14.95	1815	35.93	1682	33.3	0.038032599	15173	69.23	30.77
3'ETS_KY962518	16472_16832	361	10	2.77	48	13.3	157	43.49	146	40.44	0.03630363	15534	83.93	16.07
IGS_KY962518	16833_48338	29305	4585	15.65	9665	32.98	6147	20.98	8908	30.4	-0.183394221	44839	51.37	48.63
entire_rdna_KY962518	1299_46136	44838	6434	14.35	12368	27.58	11682	26.05	14354	32.01	-0.102627132	NA	58.07	41.93
coding_rdna	3501_16832	13332	1601	12.01	2073	15.55	4917	36.88	4741	35.56	0.008104908	103009	72.44	27.56
no_igs_KY962518	1299_16832	15534	1849	11.9	2704	17.41	5535	35.63	5446	35.06	-0.065759637	118543	70.69	29.31

Supplementary Table 2. Mapping and characterization of R-loop forming sequences (RLFSs) in the human rDNA locus.

rDNA_region	#Name	model	location:RLFS_start-end	length_RLFS	start_RIZ	end_RIZ	length_RIZ	G_RIZ	3Gs_RIZ	4Gs_RIZ	perc_G_RIZ	Linker	start_REZ	end_REZ	length_REZ	G_REZ	3Gs_REZ	4Gs_REZ	perc_G_REZ	strand
18S	KY962518	m1	KY962518:5647-7660	2014	7646	7660	14	10	3	0	71.43	0	5646	7646	2000	806	84	35	40.3	-
18S	KY962518	m1	KY962518:5393-7417	2025	7393	7417	24	15	3	2	62.5	1	5392	7392	2000	800	84	34	40	-
18S	KY962518	m2	KY962518:5403-7417	2015	7402	7417	15	11	2	2	73.33	0	5402	7402	2000	802	85	34	40.1	-
28S	KY962518	m1	KY962518:12007-13075	1069	12006	12025	19	13	3	1	68.42	0	12025	13075	1050	420	49	24	40	+
28S	KY962518	m1	KY962518:12029-13048	1020	12028	12041	13	11	3	1	84.62	0	12041	13048	1007	403	46	23	40.02	+
28S	KY962518	m1	KY962518:12043-12850	808	12042	12058	16	13	3	3	81.25	0	12058	12850	792	317	37	18	40.03	+
28S	KY962518	m1	KY962518:12140-12849	710	12139	12165	26	16	3	1	61.54	47	12212	12849	637	255	30	15	40.03	+
28S	KY962518	m1	KY962518:12249-13001	753	12248	12263	15	11	3	0	73.33	1	12264	13001	737	295	31	16	40.03	+
28S	KY962518	m1	KY962518:12477-13072	596	12476	12488	12	10	3	1	83.33	39	12527	13072	545	218	22	11	40	+
28S	KY962518	m1	KY962518:12551-13043	493	12550	12579	29	21	5	3	72.41	27	12606	13043	437	175	16	7	40.05	+
28S	KY962518	m1	KY962518:12607-12931	325	12606	12626	20	12	3	0	60	3	12629	12931	302	121	9	6	40.07	+
28S	KY962518	m1	KY962518:13721-14830	1110	13720	13735	15	10	3	0	66.67	25	13760	14830	1070	428	50	20	40	+
28S	KY962518	m1	KY962518:14303-15094	792	14302	14319	17	11	3	0	64.71	0	14319	15094	775	310	35	20	40	+
28S	KY962518	m1	KY962518:14322-15040	719	14321	14342	21	14	3	2	66.67	1	14343	15040	697	279	31	18	40.03	+
28S	KY962518	m1	KY962518:14502-15196	695	14501	14534	33	24	4	3	72.73	0	14534	15196	662	265	29	14	40.03	+
28S	KY962518	m1	KY962518:14539-15171	633	14538	14556	18	13	3	2	72.22	0	14556	15171	615	246	26	12	40	+
28S	KY962518	m1	KY962518:14608-15093	486	14607	14630	23	15	5	2	65.22	1	14631	15093	462	185	17	8	40.04	+
28S	KY962518	m1	KY962518:14778-15040	263	14777	14797	20	15	3	2	75	1	14798	15040	242	97	9	5	40.08	+
28S	KY962518	m1	KY962518:14812-15030	219	14811	14827	16	13	3	2	81.25	33	14860	15030	170	68	6	3	40	+
28S	KY962518	m1	KY962518:15406-15679	274	15405	15421	16	12	3	3	75	48	15469	15679	210	84	9	5	40	+
28S	KY962518	m1	KY962518:15491-15679	189	15490	15511	21	14	3	2	66.67	11	15522	15679	157	63	6	3	40.13	+
28S	KY962518	m1	KY962518:16090-16219	130	16199	16219	20	16	3	3	80	0	16089	16199	110	44	4	2	40	-
28S	KY962518	m1	KY962518:14053-14955	903	14933	14955	22	16	3	2	72.73	1	14052	14932	880	352	46	25	40	-
28S	KY962518	m1	KY962518:14106-14880	775	14856	14880	24	18	5	3	75	1	14105	14855	750	300	38	21	40	-
28S	KY962518	m1	KY962518:14116-14854	739	14835	14854	19	16	5	2	84.21	45	14115	14790	675	270	33	19	40	-
28S	KY962518	m1	KY962518:14168-14752	585	14737	14752	15	14	4	3	93.33	0	14167	14737	570	228	28	16	40	-
28S	KY962518	m1	KY962518:14175-14684	510	14664	14684	20	11	3	1	55	3	14174	14661	487	195	23	14	40.04	-
28S	KY962518	m1	KY962518:14174-14624	451	14598	14624	26	13	3	1	50	0	14173	14598	425	170	20	13	40	-
28S	KY962518	m1	KY962518:14165-14501	337	14481	14501	20	16	4	3	80	0	14164	14481	317	127	15	9	40.06	-
28S	KY962518	m1	KY962518:14247-14458	212	14443	14458	15	12	3	2	80	0	14246	14443	197	79	10	6	40.1	-
28S	KY962518	m1	KY962518:14292-14423	132	14408	14423	15	13	3	3	86.67	0	14291	14408	117	47	6	3	40.17	-
28S	KY962518	m1	KY962518:13461-13665	205	13647	13665	18	13	3	3	72.22	0	13460	13647	187	75	10	5	40.11	-
28S	KY962518	m1	KY962518:13515-13644	130	13630	13644	14	11	3	1	78.57	1	13514	13629	115	46	5	4	40	-
28S	KY962518	m1	KY962518:10718-12729	2012	12717	12729	12	9	3	0	75	0	10717	12717	2000	814	102	42	40.7	-
28S	KY962518	m1	KY962518:10528-12544	2017	12527	12544	17	14	3	3	82.35	0	10527	12527	2000	810	101	37	40.5	-
28S	KY962518	m1	KY962518:10529-12432	1904	12409	12432	23	14	3	2	60.87	1	10528	12408	1880	752	93	32	40	-
28S	KY962518	m1	KY962518:10710-12246	1537	12224	12246	22	16	4	2	72.73	0	10709	12224	1515	606	74	26	40	-
28S	KY962518	m1	KY962518:10717-12003	1287	11976	12003	27	21	3	3	77.78	0	10716	11976	1260	504	67	20	40	-
28S	KY962518	m1	KY962518:10727-11974	1248	11963	11974	11	9	3	0	81.82	0	10726	11963	1237	495	64	20	40.02	-
28S	KY962518	m1	KY962518:10752-11959	1208	11938	11959	21	15	4	2	71.43	0	10751	11938	1187	475	60	18	40.02	-
28S	KY962518	m1	KY962518:9530-11551	2022	11529	11551	22	14	3	2	63.64	0	9529	11529	2000	801	89	33	40.05	-
28S	KY962518	m2	KY962518:12029-13043	1015	12028	12046	18	15	4	2	83.33	2	12048	13043	995	398	45	22	40	+
28S	KY962518	m2	KY962518:12049-12850	802	12048	12058	10	9	2	2	90	0	12058	12850	792	317	37	18	40.03	+
28S	KY962518	m2	KY962518:12162-13011	850	12161	12181	20	14	3	2	70	48	12229	13011	782	313	34	16	40.03	+
28S	KY962518	m2	KY962518:12437-13043	607	12436	12457	21	12	3	2	57.14	16	12473	13043	570	228	25	12	40	+
28S	KY962518	m2	KY962518:12574-13043	470	12573	12584	11	10	3	2	90.91	22	12606	13043	437	175	16	7	40.05	+
28S	KY962518	m2	KY962518:12630-12871	242	12629	12649	20	12	3	2	60	17	12666	12871	205	82	5	3	40	+
28S	KY962518	m2	KY962518:13581-13771	191	13580	13594	14	11	2	2	78.57	45	13639	13771	132	53	4	0	40.15	+
28S	KY962518	m2	KY962518:14322-15042	721	14321	14337	16	11	2	2	68.75	0	14337	15042	705	282	32	18	40	+
28S	KY962518	m2	KY962518:14502-15233	732	14501	14521	20	14	2	2	70	0	14521	15233	712	285	31	15	40.03	+
28S	KY962518	m2	KY962518:14529-15184	656	14528	14547	19	14	4	2	73.68	0	14547	15184	637	255	27	13	40.03	+
28S	KY962518	m2	KY962518:14574-15127	554	14573	14592	19	13	2	2	68.42	10	14602	15127	525	210	22	10	40	+
28S	KY962518	m2	KY962518:14778-15044	267	14777	14787	10	9	2	2	90	0	14787	15044	257	103	10	5	40.08	+
28S	KY962518	m2	KY962518:14819-15030	212	14818	14827	9	8	2	2	88.89	33	14860	15030	170	68	6	3	40	+
28S	KY962518	m2	KY962518:15406-15656	251	15405	15416	11	8	2	2	72.73	45	15461	15656	195	78	8	5	40	+
28S	KY962518	m2	KY962518:15491-15679	189	15490	15511	21	14	3	2	66.67	11	15522	15679	157	63	6	3	40.13	+
28S	KY962518	m2	KY962518:16090-16219	130	16199	16219	20	16	3	3	80	0	16089	16199	110	44	4	2	40	-
28S	KY962518	m2	KY962518:14053-14955	903	14933	14955	22	16	3	2	72.73	1	14052	14932	880	352	46	25	40	-
28S	KY962518	m2	KY962518:14089-14880	792	14865	14880	15	12	4	2	80	0	14088	14865	777	311	40	22	40.03	-
28S	KY962518	m2	KY962518:14155-14860	706	14847	14860	13	11	3	2	84.62	1	14154	14846	692	277	35	20	40.03	-
28S	KY962518	m2	KY962518:14168-14752	585	14737	14752	15	14	4	3	93.33	0	14167	14737	570	228	28	16	40	-
28S	KY962518	m2	KY962518:14154-14501	348	14490	14501	11	10	3	2	90.91	0	14153	14490	337	135	16	10	40.06	-
28S	KY962518	m2	KY962518:14172-14485	314	14473	14485	12	10	2	2	83.33	0	14171	14473	302	121	14	8	40.07	-
28S	KY962518	m2	KY962518:14247-14452	206	14443	14452	9	8	2	2	88.89	0	14246	14443	197	79	10	6	40.1	-
28S	KY962518	m2	KY962518:14279-14423	145	14414	14423	9	8	2	2	88.89	1	14278	14413	135	54	7	4	40	-
28S	KY962518	m2	KY962518:13425-13665	241	13652	13665	13	9	2	2	69.23	1	13424	13651	227	91	12	6	40.09	-
28S	KY962518	m2	KY962518:13465-13651	187	13640	13651	11	9	2	2	81.82	1	13464	13639	175	70	9	4	40	-
28S	KY962518	m2	KY962518:10593-12606	2014	12592	12606	14	9	2	2	64.29	0	10592	12592	2000	807	101	40	40.35	-
28S	KY962518	m2	KY962518:10535-12544	2010	12534	12544	10	9	2	2	90	0	10534	12534	2000	812	102	38	40.6	-
28S	KY962518	m2	KY962518:10520-12531	2012	12519	12531	12	10	2	2	83.33	0	10519	12519	2000	804	100	36	40.2	-
28S	KY962518	m2	KY962518:10527-12432	1906	12413	12432	19	11	2	2	57.89	0	10526	12413	1887	755	94	32	40.01	-
28S	KY962518	m2	KY962518:10710-12236	1527	12224	12236	12	10	3	2	83.33	0	10709	12224	1515	606	74	26	40	-
28S	KY962518	m2	KY962518:10705-12003	1299	11985	12003	18	14	2	2	77.78	1	10704	11984	1280	512	68	21	40	-
28S	KY962518	m2	KY962518:9543-11551	2009	11542	11551	9	8	2	2	88.89	0	9542	11542	2000	803	90	33	40.15	-
3'ETS	KY962518	m1	KY962518:16580-17095	516	16579	16604	25	20	4	2	80	44	16648	17095	447	179	22	8	40.04	+
3'ETS	KY962518	m1	KY962518:16689-17125	437	16688	16702	14	10	3	1	71.43	3	16705	17125	420	168	19	7	40	+
3'ETS	KY962518	m1	KY962518:16777-17079	303	16776	16805	29	18	3	2	62.07	49	16854	17079	225	90	11	2	40	+
3'ETS	KY962518	m1	KY962518:16471-16827	357	16811	16827	16	9	3	0	56.25	1	16470	16810	340	136	13	5	40	-

3'ETS	KY962518	m1	KY962518:16448-16776	329	16758	16776	18	12	3	1	66.67	46	16447	16712	265	106	9	3	40	-
3'ETS	KY962518	m2	KY962518:16497-17231	735	16496	16512	16	12	3	2	75	47	16559	17231	672	269	30	11	40.03	+
3'ETS	KY962518	m2	KY962518:16592-17095	504	16591	16604	13	12	3	2	92.31	44	16648	17095	447	179	22	8	40.04	+
3'ETS	KY962518	m2	KY962518:16699-17095	397	16698	16709	11	8	2	2	72.73	1	16710	17095	385	154	17	5	40	+
3'ETS	KY962518	m2	KY962518:16802-17079	278	16801	16818	17	10	2	2	58.82	36	16854	17079	225	90	11	2	40	+
3'ETS	KY962518	m2	KY962518:16112-16633	522	16615	16633	18	11	2	2	61.11	32	16111	16583	472	189	17	7	40.04	-
5'ETS	KY962518	m1	KY962518:3704-5711	2008	3703	3729	26	19	3	2	73.08	2	3731	5711	1980	792	76	28	40	+
5'ETS	KY962518	m1	KY962518:4145-6162	2018	4144	4162	18	13	3	1	72.22	0	4162	6162	2000	810	74	29	40.5	+
5'ETS	KY962518	m1	KY962518:4709-6764	2056	4708	4733	25	18	4	2	72	31	4764	6764	2000	800	76	29	40	+
5'ETS	KY962518	m1	KY962518:5055-7078	2024	5054	5078	24	17	4	2	70.83	0	5078	7078	2000	808	77	28	40.4	+
5'ETS	KY962518	m1	KY962518:5905-7190	1286	5904	5929	25	14	3	1	56	1	5930	7190	1260	504	49	19	40	+
5'ETS	KY962518	m1	KY962518:6007-7020	1014	6006	6033	27	17	4	2	62.96	0	6033	7020	987	395	39	12	40.02	+
5'ETS	KY962518	m1	KY962518:6461-7167	707	6460	6476	16	13	4	2	81.25	1	6477	7167	690	276	30	10	40	+
5'ETS	KY962518	m1	KY962518:6518-7147	630	6517	6530	13	11	3	1	84.62	0	6530	7147	617	247	25	8	40.03	+
5'ETS	KY962518	m1	KY962518:6606-7147	542	6605	6632	27	15	3	1	55.56	0	6632	7147	515	206	22	7	40	+
5'ETS	KY962518	m1	KY962518:6737-7135	399	6736	6757	21	13	3	2	61.9	1	6758	7135	377	151	15	5	40.05	+
5'ETS	KY962518	m1	KY962518:6841-7135	295	6840	6854	14	10	3	0	71.43	14	6868	7135	267	107	11	5	40.07	+
5'ETS	KY962518	m1	KY962518:5023-7044	2022	7022	7044	22	14	3	0	63.64	0	5022	7022	2000	813	92	34	40.65	-
5'ETS	KY962518	m1	KY962518:5001-7021	2021	7000	7021	21	15	3	1	71.43	0	5000	7000	2000	807	90	33	40.35	-
5'ETS	KY962518	m1	KY962518:4974-6992	2019	6973	6992	19	13	3	2	68.42	0	4973	6973	2000	800	87	31	40	-
5'ETS	KY962518	m1	KY962518:5130-6599	1470	6582	6599	17	12	3	1	70.59	18	5129	6564	1435	574	70	23	40	-
5'ETS	KY962518	m1	KY962518:5227-6517	1291	6494	6517	23	14	3	2	60.87	28	5226	6466	1240	496	59	20	40	-
5'ETS	KY962518	m1	KY962518:5227-6393	1167	6377	6393	16	9	3	0	56.25	1	5226	6376	1150	460	54	19	40	-
5'ETS	KY962518	m1	KY962518:5464-6191	728	6169	6191	22	15	3	2	68.18	1	5463	6168	705	282	32	13	40	-
5'ETS	KY962518	m1	KY962518:5529-6146	618	6125	6146	21	13	3	3	61.9	7	5528	6118	590	236	27	8	40	-
5'ETS	KY962518	m1	KY962518:5518-5733	216	5718	5733	15	12	3	1	80	1	5517	5717	200	80	12	1	40	-
5'ETS	KY962518	m1	KY962518:5529-5714	186	5695	5714	19	11	3	0	57.89	2	5528	5693	165	66	9	1	40	-
5'ETS	KY962518	m1	KY962518:4723-4845	123	4827	4845	18	11	3	1	61.11	0	4722	4827	105	42	3	2	40	-
5'ETS	KY962518	m1	KY962518:3952-4398	447	4380	4398	18	14	3	2	77.78	44	3951	4336	385	154	16	5	40	-
5'ETS	KY962518	m1	KY962518:3545-4273	729	4250	4273	23	13	3	0	56.52	11	3544	4239	695	278	26	10	40	-
5'ETS	KY962518	m1	KY962518:3543-3680	138	3659	3680	21	12	3	0	57.14	0	3542	3659	117	47	2	0	40.17	-
5'ETS	KY962518	m2	KY962518:3704-5716	2013	3703	3716	13	11	2	2	84.62	0	3716	5716	2000	800	77	28	40	+
5'ETS	KY962518	m2	KY962518:4455-6470	2016	4454	4465	11	8	2	2	72.73	5	4470	6470	2000	800	72	27	40	+
5'ETS	KY962518	m2	KY962518:4717-6764	2048	4716	4733	17	13	3	2	76.47	31	4764	6764	2000	800	76	29	40	+
5'ETS	KY962518	m2	KY962518:5055-7067	2013	5054	5067	13	11	3	2	84.62	0	5067	7067	2000	812	78	28	40.6	+
5'ETS	KY962518	m2	KY962518:5492-7061	1570	5491	5503	12	8	2	2	66.67	3	5506	7061	1555	622	60	22	40	+
5'ETS	KY962518	m2	KY962518:6007-7030	1024	6006	6022	16	12	3	2	75	3	6025	7030	1005	402	40	12	40	+
5'ETS	KY962518	m2	KY962518:6466-7167	702	6465	6476	11	10	3	2	90.91	1	6477	7167	690	276	30	10	40	+
5'ETS	KY962518	m2	KY962518:6737-7145	409	6736	6753	17	10	2	2	58.82	0	6753	7145	392	157	16	5	40.05	+
5'ETS	KY962518	m2	KY962518:4974-6983	2010	6973	6983	10	8	2	2	80	0	4973	6973	2000	800	87	31	40	-
5'ETS	KY962518	m2	KY962518:5227-6512	1286	6494	6512	18	11	2	2	61.11	28	5226	6466	1240	496	59	20	40	-
5'ETS	KY962518	m2	KY962518:5464-6181	718	6169	6181	12	9	2	2	75	1	5463	6168	705	282	32	13	40	-
5'ETS	KY962518	m2	KY962518:5536-6146	611	6136	6146	10	8	2	2	80	6	5535	6130	595	238	28	9	40	-
5'ETS	KY962518	m2	KY962518:3952-4398	447	4384	4398	14	11	2	2	78.57	48	3951	4336	385	154	16	5	40	-
5'ETS	KY962518	m2	KY962518:3545-4044	500	4028	4044	16	9	2	2	56.25	49	3544	3979	435	174	15	5	40	-
5'ETS	KY962518	m2	KY962518:3506-3856	351	3846	3856	10	8	2	2	80	1	3505	3845	340	136	11	3	40	-
5'ETS	KY962518	m2	KY962518:3538-3791	254	3782	3791	9	8	2	2	88.89	0	3537	3782	245	98	9	1	40	-
IGS	KY962518	m1	KY962518:22275-22506	232	22274	22304	30	18	3	1	60	2	22306	22506	200	80	7	2	40	+
IGS	KY962518	m1	KY962518:22410-22617	208	22409	22425	16	11	3	1	68.75	15	22440	22617	177	71	2	1	40.11	+
IGS	KY962518	m1	KY962518:36977-37100	124	36976	36990	14	12	3	2	85.71	0	36990	37100	110	44	7	4	40	+
IGS	KY962518	m1	KY962518:42905-43029	125	43006	43029	23	14	3	2	60.87	2	42904	43004	100	40	5	1	40	-
IGS	KY962518	m1	KY962518:36690-36900	211	36879	36900	21	15	3	2	71.43	0	36689	36879	190	76	11	5	40	-
IGS	KY962518	m1	KY962518:33622-33844	223	33828	33844	16	13	3	3	81.25	0	33621	33828	207	83	14	7	40.1	-
IGS	KY962518	m1	KY962518:27749-27863	115	27852	27863	11	9	3	0	81.82	2	27748	27850	102	41	0	0	40.2	-
IGS	KY962518	m1	KY962518:22027-22186	160	22171	22186	15	12	3	1	80	0	22026	22171	145	58	2	1	40	-
IGS	KY962518	m1	KY962518:21314-21581	268	21565	21581	16	12	3	1	75	0	21313	21565	252	101	11	6	40.08	-
IGS	KY962518	m1	KY962518:21332-21558	227	21545	21558	13	12	3	2	92.31	2	21331	21543	212	85	8	4	40.09	-
IGS	KY962518	m1	KY962518:19813-20031	219	20018	20031	13	10	3	1	76.92	4	19812	20014	202	81	5	1	40.1	-
IGS	KY962518	m1	KY962518:16484-18500	2017	18484	18500	16	12	3	1	75	1	16483	18483	2000	800	62	13	40	-
IGS	KY962518	m1	KY962518:16088-17766	1679	17754	17766	12	10	3	1	83.33	0	16087	17754	1667	667	57	15	40.01	-
IGS	KY962518	m1	KY962518:16112-17753	1642	17742	17753	11	9	3	0	81.82	1	16111	17741	1630	652	54	15	40	-
IGS	KY962518	m1	KY962518:16448-17741	1294	17730	17741	11	9	3	0	81.82	1	16447	17729	1282	513	39	8	40.02	-
IGS	KY962518	m1	KY962518:16503-17729	1227	17718	17729	11	9	3	0	81.82	19	16502	17699	1197	479	35	8	40.02	-
IGS	KY962518	m2	KY962518:36981-37100	120	36980	36990	10	9	2	2	90	0	36990	37100	110	44	7	4	40	+
IGS	KY962518	m2	KY962518:42905-43017	113	43006	43017	11	9	2	2	81.82	2	42904	43004	100	40	5	1	40	-
IGS	KY962518	m2	KY962518:36690-36888	199	36879	36888	9	8	2	2	88.89	0	36689	36879	190	76	11	5	40	-
IGS	KY962518	m2	KY962518:33618-33844	227	33834	33844	10	9	2	2	90	0	33617	33834	217	87	15	8	40.09	-
IGS	KY962518	m2	KY962518:21332-21558	227	21545	21558	13	12	3	2	92.31	2	21331	21543	212	85	8	4	40.09	-
ITS1	KY962518	m1	KY962518:9199-10874	1676	9198	9214	16	12	3	1	75	0	9214	10874	1660	664	69	34	40	+
ITS1	KY962518	m1	KY962518:9273-10837	1565	9272	9292	20	15	3	2	75	10	9302	10837	1535	614	65	32	40	+
ITS1	KY962518	m1	KY962518:9323-9453	131	9322	9343	21	15	3	1	71.43	0	9343	9453	110	44	4	3	40	+
ITS1	KY962518	m1	KY962518:9527-10852	1326	9526	9547	21	13	3	1	61.9	25	9572	10852	1280	512	54	27	40	+
ITS1	KY962518	m1	KY962518:9662-10856	1195	9661	9681	20	15	3	1	75	0	9681	10856	1175	470	49	25	40	+
ITS1	KY962518	m1	KY962518:9687-10852	1166	9686	9708	22	14	3	2	63.64	12	9720	10852	1132	453	46	23	40.02	+
ITS1	KY962518	m1	KY962518:9853-10798	946	9852	9871	19	11	3	1	57.89	5	9876	10798	922	369	40	20	40.02	+
ITS1	KY962518	m1	KY962518:10013-10854	842	10012	10027	15	14	4	3	93.33	0	10027	10854	827	331	34	16	40.02	+
ITS1	KY962518	m1	KY962518:9033-9721	689	9708	9721	13	10	3	1	76.92	41	9032	9667	635	254	28	13	40	-
ITS1	KY962518	m1	KY962518:9253-9522	270	9496	9522	26	17	3	2	65.38	2	9252	9494	242	97	8	5	40.08	-
ITS1	KY962518	m2	KY962518:10013-10854	842	10012	10027	15	14	4	3	93.33	0	10027	10854	827	331	34	16	40.02	+
ITS1	KY962518	m2	KY962518:9047-9569	523	9559	9569	10	9	2	2	90	33	9046	9526	480	192	20	9	40	-
ITS1	KY962518	m2	KY962518:9253-9513	261	9496	9513	17	12	2	2	70.59	2	9252	9494	242	97	8	5	40.08	-
ITS2	KY962518	m1	KY962518:10271-11452	1182	10270	10295	25	13	3	2	52	0	10295	11452	1157	463	43	22	40.02	+

ITS2	KY962518	m1	KY962518:10305-11452	1148	10304	10333	29	15	3	2	51.72	12	10345	11452	1107	443	40	20	40.02	+
ITS2	KY962518	m1	KY962518:10435-11460	1026	10434	10457	23	19	5	2	82.61	1	10458	11460	1002	401	34	18	40.02	+
ITS2	KY962518	m1	KY962518:10733-10863	131	10732	10751	19	12	3	1	63.16	0	10751	10863	112	45	3	2	40.18	+
ITS2	KY962518	m1	KY962518:11194-11383	190	11193	11211	18	13	3	2	72.22	42	11253	11383	130	52	6	2	40	+
ITS2	KY962518	m1	KY962518:9321-11343	2023	11320	11343	23	15	3	1	65.22	0	9320	11320	2000	823	91	36	41.15	-
ITS2	KY962518	m1	KY962518:9243-11262	2020	11242	11262	20	13	3	1	65	0	9242	11242	2000	808	88	37	40.4	-
ITS2	KY962518	m1	KY962518:9234-11239	2006	11225	11239	14	11	3	1	78.57	0	9233	11225	1992	797	85	36	40.01	-
ITS2	KY962518	m1	KY962518:9295-11004	1710	10987	11004	17	12	3	0	70.59	1	9294	10986	1692	677	73	33	40.01	-
ITS2	KY962518	m1	KY962518:9378-10940	1563	10929	10940	11	9	3	0	81.82	0	9377	10929	1552	621	66	30	40.01	-
ITS2	KY962518	m1	KY962518:10712-10912	201	10891	10912	21	15	3	1	71.43	0	10711	10891	180	72	9	5	40	-
ITS2	KY962518	m1	KY962518:10732-10890	159	10863	10890	27	19	3	3	70.37	0	10731	10863	132	53	6	2	40.15	-
ITS2	KY962518	m1	KY962518:9260-10418	1159	10399	10418	19	14	3	2	73.68	0	9259	10399	1140	456	45	23	40	-
ITS2	KY962518	m1	KY962518:9344-10359	1016	10333	10359	26	15	3	1	57.69	5	9343	10328	985	394	37	19	40	-
ITS2	KY962518	m2	KY962518:10292-11444	1153	10291	10309	18	13	2	2	72.22	5	10314	11444	1130	452	42	21	40	+
ITS2	KY962518	m2	KY962518:10442-11460	1019	10441	10457	16	14	4	2	87.5	1	10458	11460	1002	401	34	18	40.02	+
ITS2	KY962518	m2	KY962518:10611-11408	798	10610	10621	11	8	2	2	72.73	0	10621	11408	787	315	25	14	40.03	+
ITS2	KY962518	m2	KY962518:11141-11383	243	11140	11149	9	8	2	2	88.89	42	11191	11383	192	77	9	4	40.1	+
ITS2	KY962518	m2	KY962518:11194-11383	190	11193	11211	18	13	3	2	72.22	42	11253	11383	130	52	6	2	40	+
ITS2	KY962518	m2	KY962518:9230-11246	2017	11229	11246	17	12	3	2	70.59	0	9229	11229	2000	800	86	36	40	-
ITS2	KY962518	m2	KY962518:10723-10890	168	10872	10890	18	12	2	2	66.67	0	10722	10872	150	60	7	3	40	-
ITS2	KY962518	m2	KY962518:10748-10868	121	10852	10868	16	13	2	2	81.25	0	10747	10852	105	42	5	1	40	-
ITS2	KY962518	m2	KY962518:9260-10410	1151	10399	10410	11	10	2	2	90.91	0	9259	10399	1140	456	45	23	40	-
Promoter	KY962518	m1	KY962518:2489-3569	1081	2488	2506	18	13	3	3	72.22	48	2554	3569	1015	406	32	13	40	+
Promoter	KY962518	m1	KY962518:2840-4803	1964	2839	2860	21	14	3	1	66.67	36	2896	4803	1907	763	71	29	40.01	+
Promoter	KY962518	m1	KY962518:3274-4897	1624	3273	3288	15	11	3	0	73.33	4	3292	4897	1605	642	63	24	40	+
Promoter	KY962518	m1	KY962518:3324-4897	1574	3323	3346	23	12	3	1	52.17	1	3347	4897	1550	620	60	23	40	+
Promoter	KY962518	m1	KY962518:2843-3065	223	3049	3065	16	13	3	2	81.25	17	2842	3032	190	76	10	1	40	-
Promoter	KY962518	m1	KY962518:2828-2967	140	2954	2967	13	9	3	0	69.23	7	2827	2947	120	48	6	0	40	-
Promoter	KY962518	m1	KY962518:2081-2391	311	2380	2391	11	9	3	0	81.82	0	2080	2380	300	120	21	3	40	-
Promoter	KY962518	m1	KY962518:2112-2379	268	2368	2379	11	9	3	0	81.82	0	2111	2368	257	103	17	3	40.08	-
Promoter	KY962518	m1	KY962518:2118-2267	150	2249	2267	18	15	4	3	83.33	0	2117	2249	132	53	10	0	40.15	-
Promoter	KY962518	m2	KY962518:2489-3458	970	2488	2498	10	8	2	2	80	0	2498	3458	960	384	30	13	40	+
Promoter	KY962518	m2	KY962518:3033-4897	1865	3032	3044	12	9	2	2	75	18	3062	4897	1835	734	70	26	40	+
Promoter	KY962518	m2	KY962518:2861-3065	205	3053	3065	12	10	2	2	83.33	1	2860	3052	192	77	10	1	40.1	-
Promoter	KY962518	m2	KY962518:2112-2267	156	2256	2267	11	10	3	2	90.91	0	2111	2256	145	58	11	1	40	-

Supplementary Table 3. Mapping and characterization of predicted G-quadruplex forming sequences (G4FS) in the human rDNA locus.

rDNA_region	GenBank_Accession	G4FS_start	G4FS_end	G4FS_length	G4FS_sequence	G4FS_name	strand
28S	KY962518	12006	12033	27	GGGGGCGGGCTCCGGCGGGTGCGGGGG	G4CKY962518000012006	+
28S	KY962518	12007	12033	26	GGGGCGGGCTCCGGCGGGTGCGGGGG	G4CKY962518000012007	+
28S	KY962518	12008	12033	25	GGGCGGGCTCCGGCGGGTGCGGGGG	G4CKY962518000012008	+
28S	KY962518	12012	12037	25	GGGCTCCGGCGGGTGCGGGGGTGGG	G4CKY962518000012012	+
28S	KY962518	12022	12041	19	GGGTGCGGGGGTGGGCGGG	G4CKY962518000012022	+
28S	KY962518	12028	12046	18	GGGGGTGGGCGGGCGGGG	G4CKY962518000012028	+
28S	KY962518	12029	12046	17	GGGGTGGGCGGGCGGGG	G4CKY962518000012029	+
28S	KY962518	12030	12046	16	GGGTGGGCGGGCGGGG	G4CKY962518000012030	+
28S	KY962518	12034	12053	19	GGGCGGGCGGGGCCGGGGG	G4CKY962518000012034	+
28S	KY962518	12038	12058	20	GGGCGGGGCCGGGGGTGGGG	G4CKY962518000012038	+
28S	KY962518	12042	12068	26	GGGGCCGGGGGTGGGGTCGGCGGGGG	G4CKY962518000012042	+
28S	KY962518	12043	12068	25	GGGCCGGGGGTGGGGTCGGCGGGGG	G4CKY962518000012043	+
28S	KY962518	12248	12269	21	GGGAGGGCGCGCGGGTCGGGG	G4CKY962518000012248	+
28S	KY962518	14302	14325	23	GGGCTGGGTCGGTCGGGCTGGGG	G4CKY962518000014302	+
28S	KY962518	14516	14547	31	GGGGGCGGCGGCGGGGGGAGAAGGGTCGGGG	G4CKY962518000014516	+
28S	KY962518	14517	14547	30	GGGGCGGCGGCGGGGGGAGAAGGGTCGGGG	G4CKY962518000014517	+
28S	KY962518	14518	14547	29	GGGCGGCGGCGGGGGGAGAAGGGTCGGGG	G4CKY962518000014518	+
28S	KY962518	14528	14556	28	GGGGGGAGAAGGGTCGGGGCGGCAGGGG	G4CKY962518000014528	+
28S	KY962518	14529	14556	27	GGGGGAGAAGGGTCGGGGCGGCAGGGG	G4CKY962518000014529	+
28S	KY962518	14530	14556	26	GGGGAGAAGGGTCGGGGCGGCAGGGG	G4CKY962518000014530	+
28S	KY962518	14531	14556	25	GGGAGAAGGGTCGGGGCGGCAGGGG	G4CKY962518000014531	+
28S	KY962518	11934	11959	25	GGGGTCGGGAGGAACGGGGGGCGGG	G4CKY962518000011934	-
28S	KY962518	11938	11966	28	GGGTGGAGGGGTCGGGAGGAACGGGGGG	G4CKY962518000011938	-
28S	KY962518	11939	11966	27	GGGTGGAGGGGTCGGGAGGAACGGGGG	G4CKY962518000011939	-
28S	KY962518	11940	11966	26	GGGTGGAGGGGTCGGGAGGAACGGGG	G4CKY962518000011940	-
28S	KY962518	11941	11966	25	GGGTGGAGGGGTCGGGAGGAACGGG	G4CKY962518000011941	-
28S	KY962518	11950	11970	20	GGGCGGGTGGAGGGGTCGGG	G4CKY962518000011950	-
28S	KY962518	11955	11974	19	GGGAGGGCGGGTGGAGGGG	G4CKY962518000011955	-
28S	KY962518	11956	11974	18	GGGAGGGCGGGTGGAGGG	G4CKY962518000011956	-
28S	KY962518	11963	11981	18	GGGGGAAGGGAGGGCGGG	G4CKY962518000011963	-
28S	KY962518	11967	11989	22	GGGGCGGCGGGGGAAGGGAGGG	G4CKY962518000011967	-
28S	KY962518	12212	12246	34	GGGAGGACGCGGGGCCGGGGGGCGGAGACGGGGG	G4CKY962518000012212	-
28S	KY962518	12213	12246	33	GGGAGGACGCGGGGCCGGGGGGCGGAGACGGGG	G4CKY962518000012213	-
28S	KY962518	12214	12246	32	GGGAGGACGCGGGGCCGGGGGGCGGAGACGGG	G4CKY962518000012214	-
28S	KY962518	12395	12418	23	GGGGGTGGGCGCCGGGAGGGGGG	G4CKY962518000012395	-
28S	KY962518	12396	12418	22	GGGGGTGGGCGCCGGGAGGGGG	G4CKY962518000012396	-
28S	KY962518	12397	12418	21	GGGGGTGGGCGCCGGGAGGGG	G4CKY962518000012397	-
28S	KY962518	12398	12418	20	GGGGGTGGGCGCCGGGAGGG	G4CKY962518000012398	-
28S	KY962518	12515	12539	24	GGGGGCGCGGGGAGGAGGGGTGGG	G4CKY962518000012515	-
28S	KY962518	12519	12544	25	GGGGCGGGGGCGCGGGGAGGAGGGG	G4CKY962518000012519	-
28S	KY962518	12520	12544	24	GGGGCGGGGGCGCGGGGAGGAGGG	G4CKY962518000012520	-
28S	KY962518	13622	13644	22	GGGGCGTGGGCGGGAGGAGGGG	G4CKY962518000013622	-
28S	KY962518	13623	13644	21	GGGGCGTGGGCGGGAGGAGGG	G4CKY962518000013623	-
28S	KY962518	13630	13651	21	GGGGAGCGGGGCGTGGGCGGG	G4CKY962518000013630	-
28S	KY962518	13634	13657	23	GGGGGCGGGGAGCGGGGCGTGGG	G4CKY962518000013634	-
28S	KY962518	13640	13665	25	GGGGCTCCGGGGGCGGGGAGCGGGG	G4CKY962518000013640	-
28S	KY962518	13641	13665	24	GGGGCTCCGGGGGCGGGGAGCGGG	G4CKY962518000013641	-
28S	KY962518	14372	14407	35	GGGCCGCGAGGGGGGTGCCCCGGGCGTGGGGGGGG	G4CKY962518000014372	-
28S	KY962518	14373	14407	34	GGGCCGCGAGGGGGGTGCCCCGGGCGTGGGGGGG	G4CKY962518000014373	-
28S	KY962518	14374	14407	33	GGGCCGCGAGGGGGGTGCCCCGGGCGTGGGGGG	G4CKY962518000014374	-
28S	KY962518	14375	14407	32	GGGCCGCGAGGGGGGTGCCCCGGGCGTGGGGG	G4CKY962518000014375	-
28S	KY962518	14376	14407	31	GGGCCGCGAGGGGGGTGCCCCGGGCGTGGGG	G4CKY962518000014376	-
28S	KY962518	14377	14407	30	GGGCCGCGAGGGGGGTGCCCCGGGCGTGGG	G4CKY962518000014377	-
28S	KY962518	14383	14413	30	GGGGGAGGGCCGCGAGGGGGGTGCCCCGGG	G4CKY962518000014383	-
28S	KY962518	14392	14418	26	GGGGCGGGGGAGGGCCGCGAGGGGGG	G4CKY962518000014392	-
28S	KY962518	14393	14418	25	GGGGCGGGGGAGGGCCGCGAGGGGG	G4CKY962518000014393	-
28S	KY962518	14394	14418	24	GGGGCGGGGGAGGGCCGCGAGGGG	G4CKY962518000014394	-
28S	KY962518	14395	14418	23	GGGGCGGGGGAGGGCCGCGAGGG	G4CKY962518000014395	-
28S	KY962518	14404	14423	19	GGGGTGGGGCGGGGGAGGG	G4CKY962518000014404	-
28S	KY962518	14439	14458	19	GGGCGCGGGGTGGGGAGGG	G4CKY962518000014439	-
28S	KY962518	14473	14501	28	GGGGAGGGGGGAGGACGGGGAGCGGGGG	G4CKY962518000014473	-
28S	KY962518	14474	14501	27	GGGGAGGGGGGAGGACGGGGAGCGGGG	G4CKY962518000014474	-
28S	KY962518	14475	14501	26	GGGGAGGGGGGAGGACGGGGAGCGGG	G4CKY962518000014475	-
28S	KY962518	14835	14860	25	GGGGCCGGGGGGGTAGGGCGGGGGG	G4CKY962518000014835	-
28S	KY962518	14836	14860	24	GGGGCCGGGGGGGTAGGGCGGGGG	G4CKY962518000014836	-
28S	KY962518	14837	14860	23	GGGGCCGGGGGGGTAGGGCGGGG	G4CKY962518000014837	-
28S	KY962518	14838	14860	22	GGGGCCGGGGGGGTAGGGCGGG	G4CKY962518000014838	-
28S	KY962518	14842	14871	29	GGGGGGCGGACGGGGCCGGGGGGGTAGGG	G4CKY962518000014842	-
28S	KY962518	14847	14880	33	GGGGGGAACGGGGGGCGGACGGGGCCGGGGGGG	G4CKY962518000014847	-
28S	KY962518	14848	14880	32	GGGGGGAACGGGGGGCGGACGGGGCCGGGGGG	G4CKY962518000014848	-
28S	KY962518	14849	14880	31	GGGGGGAACGGGGGGCGGACGGGGCCGGGGG	G4CKY962518000014849	-
28S	KY962518	14850	14880	30	GGGGGGAACGGGGGGCGGACGGGGCCGGGG	G4CKY962518000014850	-
28S	KY962518	14851	14880	29	GGGGGGAACGGGGGGCGGACGGGGCCGGG	G4CKY962518000014851	-
28S	KY962518	15376	15401	25	GGGGACACCGGGGGGGCGCCGGGGG	G4CKY962518000015376	-
28S	KY962518	15377	15401	24	GGGGACACCGGGGGGGCGCCGGGG	G4CKY962518000015377	-
28S	KY962518	15378	15401	23	GGGGACACCGGGGGGGCGCCGGG	G4CKY962518000015378	-
3'ETS	KY962518	16591	16608	17	GGGGGTGGGGGGGAGGG	G4CKY962518000016591	+
3'ETS	KY962518	16592	16608	16	GGGGTGGGGGGGAGGG	G4CKY962518000016592	+
3'ETS	KY962518	16593	16608	15	GGGTGGGGGGGAGGG	G4CKY962518000016593	+
3'ETS	KY962518	16688	16709	21	GGGCCGGGACGGGGTCCGGGG	G4CKY962518000016688	+
3'ETS	KY962518	16751	16776	25	GGGACGGGGACCGGCGGGCCACGGG	G4CKY962518000016751	-
5'ETS	KY962518	4708	4733	25	GGGTGGACGGGGGGGCCTGGTGGGG	G4CKY962518000004708	+
5'ETS	KY962518	6460	6482	22	GGGTCGGGGGGTGGGGCCCGGG	G4CKY962518000006460	+
5'ETS	KY962518	6465	6488	23	GGGGGGTGGGGCCCGGGCCGGGG	G4CKY962518000006465	+
5'ETS	KY962518	6466	6488	22	GGGGGTGGGGCCCGGGCCGGGG	G4CKY962518000006466	+
5'ETS	KY962518	6467	6488	21	GGGGTGGGGCCCGGGCCGGGG	G4CKY962518000006467	+
5'ETS	KY962518	6468	6488	20	GGGTGGGGCCCGGGCCGGGG	G4CKY962518000006468	+
5'ETS	KY962518	4821	4845	24	GGGACGCCTGGGGAAGGGAGGGGG	G4CKY962518000004821	-
5'ETS	KY962518	4822	4845	23	GGGACGCCTGGGGAAGGGAGGGG	G4CKY962518000004822	-
5'ETS	KY962518	4823	4845	22	GGGACGCCTGGGGAAGGGAGGG	G4CKY962518000004823	-
5'ETS	KY962518	5665	5693	28	GGGGACCGCGAGGGCAAGGGCACCCGGG	G4CKY962518000005665	-
5'ETS	KY962518	5673	5698	25	GGGCCGGGGACCGCGAGGGCAAGGG	G4CKY962518000005673	-
5'ETS	KY962518	5679	5704	25	GGGCGAGGGCCGGGGACCGCGAGGG	G4CKY962518000005679	-
5'ETS	KY962518	5689	5714	25	GGGCACAGACGGGCGAGGGCCGGGG	G4CKY962518000005689	-
5'ETS	KY962518	5690	5714	24	GGGCACAGACGGGCGAGGGCCGGG	G4CKY962518000005690	-
5'ETS	KY962518	5695	5722	27	GGGGAAGAGGGCACAGACGGGCGAGGG	G4CKY962518000005695	-
5'ETS	KY962518	5701	5726	25	GGGCGGGGAAGAGGGCACAGACGGG	G4CKY962518000005701	-
5'ETS	KY962518	5711	5733	22	GGGCGGCGGGCGGGGAAGAGGG	G4CKY962518000005711	-
5'ETS	KY962518	6968	6992	24	GGGACCGGTGGGGCCGGGGCGGGG	G4CKY962518000006968	-
5'ETS	KY962518	6969	6992	23	GGGACCGGTGGGGCCGGGGCGGG	G4CKY962518000006969	-
5'ETS	KY962518	7000	7025	25	GGGAGGGAGCGAGCGGGCGCGGGGG	G4CKY962518000007000	-
5'ETS	KY962518	7001	7025	24	GGGAGGGAGCGAGCGGGCGCGGGG	G4CKY962518000007001	-
5'ETS	KY962518	7002	7025	23	GGGAGGGAGCGAGCGGGCGCGGG	G4CKY962518000007002	-
5'ETS	KY962518	7008	7033	25	GGGCGGACGGGAGGGAGCGAGCGGG	G4CKY962518000007008	-
IGS	KY962518	24080	24110	30	GGGGGTGCGGGAATGAGGGTGTGTGTGGGG	G4CKY962518000024080	+

IGS	KY962518	24081	24110	29	GGGGTGCGGGAATGAGGGTGTGTGTGGGG	G4CKY962518000024081	+
IGS	KY962518	24082	24110	28	GGGTGCGGGAATGAGGGTGTGTGTGGGG	G4CKY962518000024082	+
IGS	KY962518	24088	24116	28	GGGAATGAGGGTGTGTGTGGGGAGGGGG	G4CKY962518000024088	+
IGS	KY962518	24096	24123	27	GGGTGTGTGTGGGGAGGGGGTGCGGGG	G4CKY962518000024096	+
IGS	KY962518	24106	24128	22	GGGGAGGGGGTGCGGGGTGGGG	G4CKY962518000024106	+
IGS	KY962518	24107	24128	21	GGGAGGGGGTGCGGGGTGGGG	G4CKY962518000024107	+
IGS	KY962518	24111	24137	26	GGGGGTGCGGGGTGGGGACGGAGGGG	G4CKY962518000024111	+
IGS	KY962518	24112	24137	25	GGGGTGCGGGGTGGGGACGGAGGGG	G4CKY962518000024112	+
IGS	KY962518	24113	24137	24	GGGTGCGGGGTGGGGACGGAGGGG	G4CKY962518000024113	+
IGS	KY962518	28954	28980	26	GGGGAGAGAGGGGGAGAGGGGGGGGG	G4CKY962518000028954	+
IGS	KY962518	28955	28980	25	GGGAGAGAGGGGGAGAGGGGGGGGG	G4CKY962518000028955	+
IGS	KY962518	36976	36995	19	GGGAGGGGTGGGGGTGGGG	G4CKY962518000036976	+
IGS	KY962518	36980	36999	19	GGGGTGGGGGTGGGGTGGG	G4CKY962518000036980	+
IGS	KY962518	36981	36999	18	GGGTGGGGGTGGGGTGGG	G4CKY962518000036981	+
IGS	KY962518	36985	37006	21	GGGGGTGGGGTGGGTTGGGGG	G4CKY962518000036985	+
IGS	KY962518	36986	37006	20	GGGGTGGGGTGGGTTGGGGG	G4CKY962518000036986	+
IGS	KY962518	36987	37006	19	GGGTGGGGTGGGTTGGGGG	G4CKY962518000036987	+
IGS	KY962518	36991	37014	23	GGGGTGGGTTGGGGGTTGTGGGG	G4CKY962518000036991	+
IGS	KY962518	36992	37014	22	GGGTGGGTTGGGGGTTGTGGGG	G4CKY962518000036992	+
IGS	KY962518	17718	17733	15	GGGAGGGAGGGAGGG	G4CKY962518000017718	-
IGS	KY962518	17722	17737	15	GGGAGGGAGGGAGGG	G4CKY962518000017722	-
IGS	KY962518	17726	17741	15	GGGAGGGAGGGAGGG	G4CKY962518000017726	-
IGS	KY962518	17730	17745	15	GGGAGGGAGGGAGGG	G4CKY962518000017730	-
IGS	KY962518	17734	17749	15	GGGAGGGAGGGAGGG	G4CKY962518000017734	-
IGS	KY962518	17738	17753	15	GGGAGGGAGGGAGGG	G4CKY962518000017738	-
IGS	KY962518	17742	17757	15	GGGAGGGAGGGAGGG	G4CKY962518000017742	-
IGS	KY962518	17746	17761	15	GGGAGGGAGGGAGGG	G4CKY962518000017746	-
IGS	KY962518	17750	17766	16	GGGGAGGGAGGGAGGG	G4CKY962518000017750	-
IGS	KY962518	18474	18491	17	GGGAGGGAGGGGGAGGG	G4CKY962518000018474	-
IGS	KY962518	18478	18500	22	GGGGAGAGAGGGAGGGAGGGGG	G4CKY962518000018478	-
IGS	KY962518	18479	18500	21	GGGGAGAGAGGGAGGGAGGGG	G4CKY962518000018479	-
IGS	KY962518	18480	18500	20	GGGGAGAGAGGGAGGGAGGG	G4CKY962518000018480	-
IGS	KY962518	21006	21022	16	GGGGGGGTGGGGGGGG	G4CKY962518000021006	-
IGS	KY962518	21007	21022	15	GGGGGGGTGGGGGGG	G4CKY962518000021007	-
IGS	KY962518	21360	21390	30	GGGGGAGAAGCGGGGACAAGGGGGAGAGGG	G4CKY962518000021360	-
IGS	KY962518	21545	21575	30	GGGTAGCGGGACGTGACGGGGGGGTGGGGG	G4CKY962518000021545	-
IGS	KY962518	21546	21575	29	GGGTAGCGGGACGTGACGGGGGGGTGGGG	G4CKY962518000021546	-
IGS	KY962518	21547	21575	28	GGGTAGCGGGACGTGACGGGGGGGTGGG	G4CKY962518000021547	-
IGS	KY962518	21551	21581	30	GGGGGAGGGTAGCGGGACGTGACGGGGGGG	G4CKY962518000021551	-
IGS	KY962518	21552	21581	29	GGGGGAGGGTAGCGGGACGTGACGGGGGG	G4CKY962518000021552	-
IGS	KY962518	21553	21581	28	GGGGGAGGGTAGCGGGACGTGACGGGGG	G4CKY962518000021553	-
IGS	KY962518	21554	21581	27	GGGGGAGGGTAGCGGGACGTGACGGGG	G4CKY962518000021554	-
IGS	KY962518	21555	21581	26	GGGGGAGGGTAGCGGGACGTGACGGG	G4CKY962518000021555	-
IGS	KY962518	22167	22186	19	GGGTGGAGGGTGGGGCGGG	G4CKY962518000022167	-
IGS	KY962518	33677	33707	30	GGGACGTGGGTAGTGGGGGGAGCCGGGGGG	G4CKY962518000033677	-
IGS	KY962518	33678	33707	29	GGGACGTGGGTAGTGGGGGGAGCCGGGGG	G4CKY962518000033678	-
IGS	KY962518	33679	33707	28	GGGACGTGGGTAGTGGGGGGAGCCGGGG	G4CKY962518000033679	-
IGS	KY962518	33680	33707	27	GGGACGTGGGTAGTGGGGGGAGCCGGG	G4CKY962518000033680	-
IGS	KY962518	33742	33781	39	GGTGTTGGGAGGCGGGGGGGGGGGGGGCGGTTTGGGG	G4CKY962518000033742	-
IGS	KY962518	33743	33781	38	GGGTGTTGGGAGGCGGGGGGGGGGGGGGCGGTTTGGG	G4CKY962518000033743	-
IGS	KY962518	33744	33781	37	GGGTGTTGGGAGGCGGGGGGGGGGGGGGCGGTTTGGG	G4CKY962518000033744	-
IGS	KY962518	33753	33781	28	GGGTGTTGGGAGGCGGGGGGGGGGGGGG	G4CKY962518000033753	-
IGS	KY962518	33754	33781	27	GGGTGTTGGGAGGCGGGGGGGGGGGGG	G4CKY962518000033754	-
IGS	KY962518	33755	33781	26	GGGTGTTGGGAGGCGGGGGGGGGGGG	G4CKY962518000033755	-
IGS	KY962518	33756	33781	25	GGGTGTTGGGAGGCGGGGGGGGGGG	G4CKY962518000033756	-
IGS	KY962518	33757	33781	24	GGGTGTTGGGAGGCGGGGGGGGGG	G4CKY962518000033757	-
IGS	KY962518	33758	33781	23	GGGTGTTGGGAGGCGGGGGGGGG	G4CKY962518000033758	-
IGS	KY962518	33759	33781	22	GGGTGTTGGGAGGCGGGGGGGG	G4CKY962518000033759	-
IGS	KY962518	33760	33781	21	GGGTGTTGGGAGGCGGGGGGG	G4CKY962518000033760	-
IGS	KY962518	36800	36822	22	GGGGCGTGGGGCGTGGGTGGGG	G4CKY962518000036800	-
IGS	KY962518	36801	36822	21	GGGGCGTGGGGCGTGGGTGGG	G4CKY962518000036801	-
IGS	KY962518	36805	36829	24	GGGACGTGGGGCGTGGGGCGTGGG	G4CKY962518000036805	-
IGS	KY962518	36811	36837	26	GGGTGCCCGGGACGTGGGGCGTGGGG	G4CKY962518000036811	-
IGS	KY962518	36812	36837	25	GGGTGCCCGGGACGTGGGGCGTGGG	G4CKY962518000036812	-
IGS	KY962518	42990	43017	27	GGGGGAAGGGGAAAGGGGGGGTCCGGG	G4CKY962518000042990	-
IGS	KY962518	42996	43017	21	GGGGGAAGGGGAAAGGGGGGG	G4CKY962518000042996	-
IGS	KY962518	43476	43503	27	GGGATCCCCTGGGGAGGGGGTGGGGGG	G4CKY962518000043476	-
IGS	KY962518	43477	43503	26	GGGATCCCCTGGGGAGGGGGTGGGGG	G4CKY962518000043477	-
IGS	KY962518	43478	43503	25	GGGATCCCCTGGGGAGGGGGTGGGG	G4CKY962518000043478	-
IGS	KY962518	43479	43503	24	GGGATCCCCTGGGGAGGGGGTGGG	G4CKY962518000043479	-
IGS	KY962518	44827	44847	20	GGGCAACCAGGGAGGGCGGG	G4CKY962518000044827	-
IGS	KY962518	44831	44857	26	GGGGTTGTCTGGGCAACCAGGGAGGG	G4CKY962518000044831	-
ITS1	KY962518	9661	9691	30	GGGTCGGGGGCGGTGGTGGGCCCGCGGGGG	G4CKY962518000009661	+
ITS1	KY962518	9852	9882	30	GGGGGCGGGAACCCCCGGGCGCCTGTGGGG	G4CKY962518000009852	+
ITS1	KY962518	9853	9882	29	GGGGCGGGAACCCCCGGGCGCCTGTGGGG	G4CKY962518000009853	+
ITS1	KY962518	9854	9882	28	GGGCGGGAACCCCCGGGCGCCTGTGGGG	G4CKY962518000009854	+
ITS2	KY962518	10434	10457	23	GGGTGGCGGGGGGGAGAGGGGGG	G4CKY962518000010434	+
ITS2	KY962518	10732	10762	30	GGGTCCGGAAGGGGAAGGGTGCCGGCGGGG	G4CKY962518000010732	+
ITS2	KY962518	10742	10770	28	GGGGAAGGGTGCCGGCGGGGAGAGAGGG	G4CKY962518000010742	+
ITS2	KY962518	10743	10770	27	GGGAAGGGTGCCGGCGGGGAGAGAGGG	G4CKY962518000010743	+
ITS2	KY962518	10748	10777	29	GGGTGCCGGCGGGGAGAGAGGGTCGGGGG	G4CKY962518000010748	+
ITS2	KY962518	10389	10410	21	GGGGGAGGGGGAAGGGGCGGG	G4CKY962518000010389	-
ITS2	KY962518	10393	10418	25	GGGCCCGCGGGGGAGGGGGAAGGGG	G4CKY962518000010393	-
ITS2	KY962518	10394	10418	24	GGGCCCGCGGGGGAGGGGGAAGGG	G4CKY962518000010394	-
ITS2	KY962518	10846	10876	30	GGGGCGGCGGGGGAGGAGGAGGGGCGCGGG	G4CKY962518000010846	-
ITS2	KY962518	10975	10994	19	GGGCGGGCCGCGGGGCGGG	G4CKY962518000010975	-
ITS2	KY962518	10979	11004	25	GGGCGCGGCCGGGCGGGCCGCGGGG	G4CKY962518000010979	-
ITS2	KY962518	10980	11004	24	GGGCGCGGCCGGGCGGGCCGCGGG	G4CKY962518000010980	-
ITS2	KY962518	11220	11239	19	GGGCCGGGGGCGGGACGGG	G4CKY962518000011220	-
ITS2	KY962518	11225	11246	21	GGGGCACGGGCCGGGGGCGGG	G4CKY962518000011225	-
ITS2	KY962518	11229	11250	21	GGGAGGGGCACGGGCCGGGGG	G4CKY962518000011229	-
ITS2	KY962518	11230	11250	20	GGGAGGGGCACGGGCCGGGG	G4CKY962518000011230	-
ITS2	KY962518	11231	11250	19	GGGAGGGGCACGGGCCGGG	G4CKY962518000011231	-
Promoter	KY962518	2182	2197	15	GGGAGGGAGGGAGGG	G4CKY962518000002182	-
Promoter	KY962518	2186	2201	15	GGGAGGGAGGGAGGG	G4CKY962518000002186	-
Promoter	KY962518	2190	2205	15	GGGAGGGAGGGAGGG	G4CKY962518000002190	-
Promoter	KY962518	2194	2212	18	GGGAGCAGGGAGGGAGGG	G4CKY962518000002194	-
Promoter	KY962518	2198	2217	19	GGGAAGGGAGCAGGGAGGG	G4CKY962518000002198	-
Promoter	KY962518	2202	2221	19	GGGAGGGAAGGGAGCAGGG	G4CKY962518000002202	-
Promoter	KY962518	2209	2229	20	GGGAAGGAGGGAGGGAAGGG	G4CKY962518000002209	-
Promoter	KY962518	2360	2375	15	GGGAGGGAGGGAGGG	G4CKY962518000002360	-
Promoter	KY962518	2364	2379	15	GGGAGGGAGGGAGGG	G4CKY962518000002364	-
Promoter	KY962518	2368	2383	15	GGGAGGGAGGGAGGG	G4CKY962518000002368	-
Promoter	KY962518	2372	2387	15	GGGAGGGAGGGAGGG	G4CKY962518000002372	-
Promoter	KY962518	2376	2391	15	GGGAGGGAGGGAGGG	G4CKY962518000002376	-

Supplementary Table 4. Mapping and characterization of predicted i-motif–forming sequences (iMFSs) in the human rDNA locus.

rDNA_region	chr	iMFS_start	iMFS_end	iMFS_length	iMFS_sequence	predict_score	predict_tranPH	strand
Promoter	KY962518	2183	2197	14	CCCTCCCTCCCTCCC	0.419	6.27	+
Promoter	KY962518	2199	2217	18	CCCTCCCTGCTCCCTTCCC	0.345	6.07	+
Promoter	KY962518	2361	2375	14	CCCTCCCTCCCTCCC	0.419	6.27	+
Promoter	KY962518	2377	2391	14	CCCTCCCTCCCTCCC	0.419	6.27	+
Promoter	KY962518	2894	2925	31	CCCGAGGCCCGAGCCGCGACCCGCGGGGACCC	0.342	6.06	+
5'ETS	KY962518	4101	4127	26	CCCCGGCCCGACCTCGCCGTCCCGCCC	0.361	6.11	+
5'ETS	KY962518	4822	4845	23	CCCCCTCCCTTCCCCAGGCGTCCC	0.305	5.95	+
5'ETS	KY962518	5666	5692	26	CCCGGGTGCCCTTGCCCTCGCGGTCCC	0.402	6.23	+
5'ETS	KY962518	5696	5721	25	CCCTCGCCCGTCTGTGCCCTCTTCCC	0.358	6.1	+
5'ETS	KY962518	6367	6393	26	CCCGAGCGCGGCCCGGTGGTCCCTCCC	0.423	6.28	+
5'ETS	KY962518	6969	6992	23	CCCCGCCCCGGCCCCACCGGTCCC	0.302	5.94	+
5'ETS	KY962518	7001	7025	24	CCCCCGCGCCCGCTCGCTCCCTCCC	0.289	5.91	+
18S	KY962518	8895	8931	36	CCCTCGGATCGGCCCCGCCGGGGTCGGCCCACGGCCC	0.502	6.51	+
ITS1	KY962518	9075	9096	21	CCCTCCGCACACCCACCCCCCC	0.214	5.7	+
ITS1	KY962518	9385	9417	32	CCCTCCTCCCCGCCGGGGCCCGTCGTCCGGCCC	0.526	6.57	+
ITS1	KY962518	9682	9716	34	CCCGCGGGGGAGTCCCGTCGGGAGGGGCCCGGCCC	0.365	6.12	+
ITS2	KY962518	10312	10344	32	CCCTCGCAGGGCCCGCCGGGGGCCCTCCGTCCC	0.482	6.45	+
ITS2	KY962518	10390	10408	18	CCCGCCCCTTCCCCCTCCC	0.204	5.67	+
ITS2	KY962518	10847	10875	28	CCCGCGCCCCTCCTCCTCCCCCGCCGCCC	0.368	6.13	+
ITS2	KY962518	10887	10911	24	CCCCGCCCGTCCTCCTCGCCCTCCC	0.286	5.9	+
ITS2	KY962518	10976	10994	18	CCCGCCCCGCGGCCCGCCC	0.348	6.07	+
ITS2	KY962518	11221	11239	18	CCCGTCCCGCCCCCGGCCC	0.365	6.12	+
ITS2	KY962518	11309	11343	34	CCCGTCGCCTCTCCCCCTCGCCGGGCCCGTCTCCC	0.502	6.51	+
28S	KY962518	11921	11953	32	CCCGGCGGATCTTTCCCGCCCCCCGTTCCTCCC	0.393	6.2	+
28S	KY962518	11956	11974	18	CCCCTCCACCCGCCCTCCC	0.179	5.6	+
28S	KY962518	12213	12246	33	CCCCCGTCTCCGCCCCCCGGCCCCGCGTCCTCCC	0.451	6.36	+
28S	KY962518	12314	12338	24	CCCCCCCGAGTGTTACAGCCCCCCC	0.261	5.83	+
28S	KY962518	12396	12416	20	CCCCCCTCCCGGCGCCCACCC	0.251	5.8	+
28S	KY962518	12516	12537	21	CCCACCCCTCCTCCCCGCGCCC	0.283	5.89	+
28S	KY962518	13599	13626	27	CCCCCGCCCCCCCCCACGCCTCCTCCCC	0.364	6.12	+
28S	KY962518	13631	13650	19	CCCGCCCACGCCCCGCTCCC	0.375	6.15	+
28S	KY962518	14373	14395	22	CCCCCCCCACGCCCGGGGCACCC	0.367	6.13	+
28S	KY962518	14396	14417	21	CCCTCGCGGCCCTCCCCCGCCC	0.368	6.13	+
28S	KY962518	14440	14458	18	CCCTCCCCACCCCGCGCCC	0.325	6.01	+
28S	KY962518	14474	14501	27	CCCCCGCTCCCCGTCCTCCCCCCTCCCC	0.388	6.19	+
28S	KY962518	14836	14880	44	CCCCCCGCCCTACCCCCCCGGCCCCGTCCGCCCCCCGTTCCCCCC	0.469	6.41	+
28S	KY962518	15377	15400	23	CCCCCGGCGCCCCCCCGGTGTCCC	0.569	6.69	+
28S	KY962518	16173	16219	46	CCCCCGCCTGTCCCCGCCGGCGGCCGCCCCCCCCTCCACGCGCCCC	0.484	6.46	+
3'ETS	KY962518	16616	16658	42	CCCCGGTCGGCGCGCCCCGCTTCTTCGGTTCCCGCCTCCTCCC	0.408	6.24	+
3'ETS	KY962518	16752	16776	24	CCCGTGGCCCGCCGGTCCCCGTCCC	0.368	6.13	+
IGS	KY962518	17719	17733	14	CCCTCCCTCCCTCCC	0.419	6.27	+
IGS	KY962518	17735	17749	14	CCCTCCCTCCCTCCC	0.419	6.27	+
IGS	KY962518	17751	17765	14	CCCTCCCTCCCTCCC	0.419	6.27	+
IGS	KY962518	18475	18491	16	CCCTCCCCCTCCCTCCC	0.316	5.99	+
IGS	KY962518	21007	21022	15	CCCCCCCCACCCCCCC	0.228	5.74	+
IGS	KY962518	21346	21378	32	CCCGAGGGCCTCCTTCCCTCTCCCCCTTGTCCC	0.443	6.34	+
IGS	KY962518	21546	21568	22	CCCCCACCCCCCCGTCACGTCCC	0.287	5.9	+
IGS	KY962518	22168	22186	18	CCCGCCCCACCCTCCACCC	0.201	5.66	+
IGS	KY962518	33504	33531	27	CCCTTCTGGAGGCCCCTCCCTCTCTCCC	0.436	6.32	+
IGS	KY962518	33678	33707	29	CCCCCCGGCTCCCCCCACTACCCACGTCCC	0.322	6	+
IGS	KY962518	33743	33767	24	CCCCCAAACCGCCCCCCCCCCCCCC	0.304	5.95	+
IGS	KY962518	34029	34062	33	CCCCCCGCTGCTTCCCGCCTCAGGCCTCCCTCCC	0.384	6.17	+
IGS	KY962518	36801	36821	20	CCCCACCCACGCCCCACGCCC	0.273	5.86	+
IGS	KY962518	42342	42371	29	CCCCAGTGGATCTGCCCGCCCCGGCCTCCC	0.354	6.09	+
IGS	KY962518	42991	43009	18	CCCGGACCCCCCCTTTCCC	0.284	5.9	+
IGS	KY962518	43477	43503	26	CCCCCCACCCCCTCCCCAGGGGATCCC	0.354	6.09	+
IGS	KY962518	44828	44847	19	CCCGCCCTCCCTGGTTGCCC	0.387	6.18	+
ITS2	KY962518	11325	11352	27	CCCCACAACCCCCAACCCACCCCACCCC	0.236	5.76	-
IGS	KY962518	19359	19384	25	CCCCCCCCCTCTCCCCCTCTCTCCCC	0.354	6.09	-
IGS	KY962518	24189	24218	29	CCCTTAGGACGCTCCCCTCCGTCCCCACCC	0.332	6.03	-
IGS	KY962518	24223	24250	27	CCCCCTCCCCACACACACCCTCATTCCC	0.166	5.56	-
IGS	KY962518	26030	26064	34	CCCGGCCCCAAGCGATCCACCCGCCTCGGCCTCCC	0.402	6.23	-
IGS	KY962518	31521	31560	39	CCCCGGGTGGCCGCCCCACCGGGGCCCGCGCGGCCAACCC	0.491	6.47	-
IGS	KY962518	31617	31645	28	CCCAAACCACGCTCCCCGGACCCCGTCCC	0.362	6.11	-
IGS	KY962518	31731	31745	14	CCCTCCCCCCCACCC	0.257	5.82	-
IGS	KY962518	33783	33821	38	CCCCTGCCGCCCCGACCCTTCTCCCCCCGCCGCCGCCCC	0.538	6.61	-
IGS	KY962518	33997	34022	25	CCCAGCCCCGCTTCGCGCCCCAGCCC	0.239	5.77	-
IGS	KY962518	35690	35728	38	CCCCGAGAGAACCTCCCCCCGGGCCCGACGGCGCGACCC	0.437	6.32	-
IGS	KY962518	35755	35783	28	CCCCGCCCCCCGACCCGCGCGCGGCACCC	0.377	6.16	-
IGS	KY962518	36070	36090	20	CCCCGACCCGCGCGCCCTCCC	0.299	5.94	-
IGS	KY962518	36159	36199	40	CCCCCGAGCCACCTTCCCCGCCGGGCCTTCCCAGCCGTCCC	0.459	6.38	-
IGS	KY962518	36271	36296	25	CCCCCGCCGACCCCACCCCCGGCCCC	0.349	6.08	-
IGS	KY962518	36298	36316	18	CCCGCCCACCCCCGCACCC	0.37	6.13	-
IGS	KY962518	37562	37590	28	CCCCCGACCCTCTCTCCCCGCCGGCACCC	0.402	6.23	-
IGS	KY962518	37882	37904	22	CCCCCCTCTCCCCCCCGCCACCC	0.253	5.81	-
IGS	KY962518	38006	38046	40	CCCCCGGCGGGCCCTGCGAGGGAACCCCCAGCCGCGCACCC	0.457	6.38	-
IGS	KY962518	38457	38484	27	CCCCACAGGCGCCCGGGGGTTCCCGCCC	0.417	6.27	-
IGS	KY962518	38631	38660	29	CCCCTCCCGACGGGACTCCCCCGCGGGCCC	0.42	6.28	-
IGS	KY962518	41700	41732	32	CCCGGGGCCCGGCCGAAGCCCGGCTCCGAGCCC	0.492	6.48	-
IGS	KY962518	41851	41870	19	CCCCGGCCCGGGCCCCACCC	0.331	6.03	-
IGS	KY962518	42401	42433	32	CCCCCGGAACCCTCCGGGAAGCCCACCGGGCCC	0.456	6.38	-
IGS	KY962518	43261	43283	22	CCCTCGCTTCTCCCCCCCAACCC	0.285	5.9	-
IGS	KY962518	43606	43630	24	CCCCACCAGGCCCCCCCGTCCACCC	0.345	6.07	-
IGS	KY962518	45470	45499	29	CCCGGGCAGCCCCACGCCCGCCGCGGGCCC	0.435	6.32	-
IGS	KY962518	45833	45865	32	CCCCTCTGCCCCAACCCCACACAAACGGTGCCC	0.4	6.22	-

Supplementary Table 5. Predicted counts G4FS, RIZ, and iMFS across bins of the human rDNA locus

Bins	G4FS_counts	RIZ_counts	imotif_counts	RIZ_bin	G4FS_bin	iMFS_bin
1	0	0	0	0	0	0
2	0	0	1	0	0	1
3	12	9	3	1	1	1
4	0	6	1	1	0	1
5	0	3	0	1	0	0
6	0	5	0	1	0	0
7	0	5	1	1	0	1
8	4	5	1	1	1	1
9	0	2	0	1	0	0
10	8	5	2	1	1	1
11	0	7	0	1	0	0
12	5	8	1	1	1	1
13	6	9	2	1	1	1
14	0	3	0	1	0	0
15	0	4	0	1	0	0
16	0	0	0	0	0	0
17	0	1	1	1	0	1
18	0	5	1	1	0	1
19	1	8	2	1	1	1
20	3	3	0	1	1	0
21	4	9	2	1	1	1
22	8	11	3	1	1	1
23	5	10	3	1	1	1
24	22	9	2	1	1	1
25	8	9	3	1	1	1
26	3	9	1	1	1	1
27	0	0	0	0	0	0
28	6	8	2	1	1	1
29	1	1	0	1	1	0
30	23	19	4	1	1	1
31	10	13	1	1	1	1
32	3	7	1	1	1	1
33	0	0	0	0	0	0
34	0	4	1	1	0	1
35	5	11	2	1	1	1
36	0	0	0	0	0	0
37	9	4	3	1	1	1
38	0	0	0	0	0	0
39	4	1	1	1	1	1
40	0	1	0	1	0	0
41	0	0	1	0	0	1
42	0	3	0	1	0	0
43	0	0	0	0	0	0
44	2	2	1	1	1	1
45	1	3	1	1	1	1
46	8	5	1	1	1	1
47	1	2	1	1	1	1
48	0	2	0	1	0	0
49	0	0	0	0	0	0

50	0	0	0	0	0	0
51	10	7	0	1	1	0
52	0	2	2	1	0	1
53	0	0	0	0	0	0
54	0	1	0	1	0	0
55	0	0	0	0	0	0
56	0	0	1	0	0	1
57	0	0	0	0	0	0
58	0	0	0	0	0	0
59	0	1	0	1	0	0
60	0	1	0	1	0	0
61	0	0	0	0	0	0
62	2	5	0	1	1	0
63	0	0	0	0	0	0
64	0	1	0	1	0	0
65	0	0	0	0	0	0
66	0	0	0	0	0	0
67	0	0	0	0	0	0
68	0	0	3	0	0	1
69	0	1	0	1	0	0
70	0	1	0	1	0	0
71	0	0	0	0	0	0
72	0	1	1	1	0	1
73	15	8	5	1	1	1
74	0	0	0	0	0	0
75	0	0	0	0	0	0
76	0	0	0	0	0	0
77	0	0	2	0	0	1
78	0	0	2	0	0	1
79	0	1	2	1	0	1
80	13	10	1	1	1	1
81	0	1	1	1	0	1
82	0	0	2	0	0	1
83	0	0	1	0	0	1
84	0	1	1	1	0	1
85	0	0	0	0	0	0
86	0	0	0	0	0	0
87	0	0	0	0	0	0
88	0	0	0	0	0	0
89	0	0	0	0	0	0
90	0	5	0	1	0	0
91	0	0	2	0	0	1
92	0	1	2	1	0	1
93	0	0	1	0	0	1
94	2	3	1	1	1	1
95	4	3	2	1	1	1
96	0	0	0	0	0	0
97	0	0	0	0	0	0
98	2	1	1	1	1	1
99	0	0	1	0	0	1
100	0	0	1	0	0	1

Supplementary Table 6. Input dataset for POLR1A ChIP-seq and non-canonical structure associations in the human rDNA locus.

					Template non-canonical structures											
GenBank ID	bin	POLR1A ChIP-seq signal	norm_POLR1A	diff_norm_POLR1A	RLFS_counts	norm_RLFS_counts	G4FS_counts	norm_G4FS_counts	iMFS_counts	norm_iMFS_counts	diff_norm_RLFS_counts	diff_norm_G4FS_counts	diff_norm_iMFS_counts	POLR1_RLFS_sign_match	POLR1_G4FS_sign_match	POLR1_iMFS_sign_match
KY962518	1	224.4598214	0.005731471	NA	0	0	0	0	0	0	NA	NA	NA	NA	NA	NA
KY962518	2	399.1135857	0.012622645	0.006891174	0	0	0	0	0	0	0	0	0	1	1	1
KY962518	3	398.8526786	0.012612351	-1.03E-05	0	0	0	0	0	0	0	0	0	1	1	1
KY962518	4	427.109375	0.013727253	0.001114902	0	0	0	0	0	0	0	0	0	1	1	1
KY962518	5	356.6659243	0.010947822	-0.002779431	0	0	0	0	1	0.333333333	0	0	0.333333333	1	1	1
KY962518	6	244.6316964	0.006527377	-0.004420445	0	0	0	0	0	0	0	0	-0.333333333	1	1	0
KY962518	7	309.6057906	0.009091008	0.002563631	0	0	0	0	0	0	0	0	0	1	1	1
KY962518	8	334.9754464	0.010091998	0.00100099	0	0	0	0	0	0	0	0	0	1	1	1
KY962518	9	391.4308036	0.012319512	0.002227514	2	0.181818182	2	0.125	0	0	0.181818182	0.125	0	0	0	1
KY962518	10	354.9910913	0.010881739	-0.001437773	0	0	0	0	0	0	-0.181818182	-0.125	0	0	0	1
KY962518	11	229.2767857	0.00592153	-0.004960209	0	0	4	0.25	0	0	0	0.25	0	1	1	1
KY962518	12	544.5401786	0.018360626	0.012439096	0	0	0	0	0	0	0	-0.25	0	1	1	1
KY962518	13	405.0957684	0.012858679	-0.005501947	0	0	0	0	0	0	0	0	0	1	1	1
KY962518	14	406.0892857	0.01289788	2004201208284e	0	0	2	0.125	0	0	0	0.125	0	1	0	1
KY962518	15	350.1848552	0.010692103	-0.002205776	0	0	0	0	0	0	0	-0.125	0	1	0	1
KY962518	16	102.7946429	0.000931025	-0.009761078	0	0	0	0	0	0	0	0	0	1	1	1
KY962518	17	424.46875	0.013623064	0.012692038	0	0	0	0	0	0	0	0	0	1	1	1
KY962518	18	79.19821826	0	-0.013623064	0	0	7	0.4375	0	0	0	0.4375	0	1	1	1
KY962518	19	558.1696429	0.018898393	0.018898393	4	0.363636364	5	0.3125	0	0	0.363636364	-0.125	0	0	1	1
KY962518	20	4850.084821	0.188241062	0.169342669	3	0.272727273	0	0	0	0	-0.090909091	-0.3125	0	1	1	1
KY962518	21	4120.2049	0.159442773	-0.028798289	0	0	0	0	0	0	-0.272727273	0	0	0	1	1
KY962518	22	10901.375	0.427001985	0.267559212	3	0.272727273	0	0	0	0	0.272727273	0	0	0	1	1
KY962518	23	13064.72829	0.512359688	0.085357703	4	0.363636364	0	0	1	0.333333333	0.090909091	0	0.333333333	0	1	0
KY962518	24	12727.54688	0.49905579	-0.013303898	1	0.090909091	3	0.1875	0	0	-0.272727273	0.1875	-0.333333333	0	1	0
KY962518	25	11616.85268	0.455232016	-0.043823774	0	0	0	0	0	0	-0.090909091	-0.1875	0	0	0	1
KY962518	26	10023.72606	0.392373292	-0.062858725	2	0.181818182	8	0.5	0	0	0.181818182	0.5	0	1	1	1
KY962518	27	6755.549107	0.263423444	-0.128949848	4	0.363636364	0	0	0	0	0.181818182	-0.5	0	1	0	1
KY962518	28	6674.073661	0.260208733	-0.003214712	4	0.363636364	0	0	0	0	0	0	0	1	1	1
KY962518	29	4002.623608	0.154803462	-0.105405271	4	0.363636364	6	0.375	0	0	0	0.375	0	1	1	1
KY962518	30	19500.57366	0.766293698	0.611490236	2	0.181818182	0	0	0	0	-0.181818182	-0.375	0	1	1	1
KY962518	31	17746.94866	0.697102317	-0.069191381	1	0.090909091	0	0	0	0	-0.090909091	0	0	0	1	1
KY962518	32	22153.07127	0.870951178	0.17384886	0	0	0	0	0	0	-0.090909091	0	0	1	1	1
KY962518	33	25423.7567	1	0.129048822	0	0	0	0	0	0	0	0	0	1	1	1
KY962518	34	7748.806236	0.302613597	-0.697386403	0	0	0	0	2	0.666666667	0	0	0.666666667	1	1	1
KY962518	35	5666.607143	0.220457931	-0.082155666	4	0.363636364	0	0	0	0	0.363636364	0	-0.666666667	1	1	0
KY962518	36	15793.5	0.620026654	0.399568723	0	0	0	0	0	0	-0.363636364	0	0	1	1	1
KY962518	37	8822.423163	0.344974443	-0.275052211	3	0.272727273	3	0.1875	0	0	0.272727273	0.1875	0	1	1	1
KY962518	38	1443.508929	0.053830518	-0.291143925	6	0.545454545	4	0.25	1	0.333333333	0.272727273	0.0625	0.333333333	1	1	1
KY962518	39	12355.4308	0.484373504	0.430542986	6	0.545454545	5	0.3125	0	0	0	0.0625	-0.333333333	1	0	1
KY962518	40	10303.99332	0.403431573	-0.08094193	4	0.363636364	10	0.625	0	0	-0.181818182	0.3125	0	0	1	1
KY962518	41	3839.084821	0.148350843	-0.255080731	7	0.636363636	10	0.625	0	0	0.272727273	0	0	1	1	1
KY962518	42	13379.28062	0.524770728	0.376419886	2	0.181818182	0	0	0	0	-0.454545455	-0.625	0	1	1	1
KY962518	43	21639.68527	0.850694916	0.325924188	0	0	0	0	0	0	-0.181818182	0	0	1	1	1
KY962518	44	11725.6183	0.459523495	-0.391171421	4	0.363636364	6	0.375	0	0	0.363636364	0.375	0	1	1	1
KY962518	45	17066.81514	0.670266832	0.210743338	0	0	0	0	0	0	-0.363636364	-0.375	0	1	1	1
KY962518	46	1382.303571	0.051415587	-0.618851245	11	1	16	1	0	0	1	1	0	1	1	1
KY962518	47	9724.464286	0.380565559	0.329149972	6	0.545454545	10	0.625	0	0	-0.454545455	-0.375	0	1	1	1
KY962518	48	14178.8686	0.556319432	0.175753873	0	0	3	0.1875	0	0	-0.545454545	-0.4375	0	1	1	1
KY962518	49	19780.52902	0.777339673	0.221020241	0	0	0	0	0	0	0	-0.1875	0	1	1	1
KY962518	50	10109.38085	0.395752904	-0.381586769	2	0.181818182	0	0	3	1	0.181818182	0	1	1	1	1
KY962518	51	2741.392857	0.105040087	-0.290712817	3	0.272727273	1	0.0625	0	0	0.090909091	0.0625	-1	1	1	0
KY962518	52	667.7633929	0.023222546	-0.081817541	0	0	0	0	0	0	-0.272727273	-0.0625	0	0	0	1
KY962518	53	1232.184855	0.045492473	0.022269927	4	0.363636364	9	0.5625	0	0	0.363636364	0.5625	0	0	0	1
KY962518	54	716.8683036	0.025160039	-0.020332434	0	0	0	0	0	0	-0.363636364	-0.5625	0	0	0	1
KY962518	55	509.9553571	0.01699604	-0.008163999	1	0.090909091	4	0.25	1	0.333333333	0.090909091	0.25	0.333333333	1	1	1
KY962518	56	403.4298441	0.012792948	-0.004203092	0	0	0	0	1	0.333333333	-0.090909091	-0.25	0	0	0	1
KY962518	57	247.6071429	0.006644776	-0.006148172	0	0	0	0	0	0	0	0	-0.333333333	1	1	0
KY962518	58	310.6919643	0.009133864	0.002489087	1	0.090909091	0	0	0	0	0.090909091	0	0	0	1	1
KY962518	59	369.8351893	0.011467431	0.002333567	0	0	0	0	2	0.666666667	-0.090909091	0	0.666666667	1	1	0
KY962518	60	381.5602679	0.011930058	0.000462627	0	0	2	0.125	2	0.666666667	0	0.125	0	1	0	1
KY962518	61	313.0824053	0.009228182	-0.002701876	0	0	1	0.0625	2	0.666666667	0	-0.0625	0	1	0	1
KY962518	62	257.8415179	0.007048586	-0.002179596	3	0.272727273	8	0.5	0	0	0.272727273	0.4375	-0.666666667	1	1	0
KY962518	63	353.4933036	0.010822642	0.003774056	1	0.090909091	1	0.0625	0	0	-0.181818182	-0.4375	0	1	1	1
KY962518	64	337.9198218	0.010208172	-0.00061447	0	0	0	0	2	0.666666667	-0.090909091	-0.0625	0.666666667	0	0	1
KY962518	65	396.2767857	0.012510716	0.002302544	0	0	0	0	1	0.333333333	0	0	-0.333333333	1	1	1
KY962518	66	431.09375	0.013884461	0.001373745	0	0	0	0	2	0.666666667	0	0	0.333333333	1	1	0
KY962518	67	323.3474388	0.009633201	-0.00425126	0	0	0	0	0	0	0	0	-0.666666667	1	1	0
KY962518	68	367.8973214	0.01139097	0.001757769	0	0	0	0	0	0	0	0	0	1	1	1
KY962518	69	396.8485523	0.012533276	0.001142306	0	0	0	0	0	0	0	0	0	1	1	1
KY962518	70	458.8459821	0.014979459	0.002446183	0	0	0	0	0	0	0	0	0	1	1	1
KY962518	71	201.7366071	0.004834899	-0.010144559	0	0	0	0	0	0	0	0	0	1	1	1
KY962518	72	355.3808463	0.010897117	0.006062218	0	0	0	0	0	0	0	0	0	1	1	1
KY962518	73	343.8080357	0.010440498	-0.000456619	0	0	0	0	2	0.666666667	0	0	0.666666667	1	1	1
KY962518	74	303.2857143	0.008841641	-0.001598857	0	0	0	0	1	0.333333333	0	0	-0.333333333	1	1	0
KY962518	75	298.9420935	0.008670259	-0.000171383	0	0	0	0	0	0	0	0	-0.333333333	1	1	0
KY962518	76	310.0982143	0.009110437	0.000440178	1	0.090909091	0	0	1	0.333333333	0.090909091	0	0.333333333	0	1	0
KY962518	77	374.40625	0.011647788	0.002537351	0	0	0	0	1	0.333333333	-0.090909091	0	0	1	1	1
KY962518	78	308.6971047	0.009055154	-0.002592633	0	0	0	0	0	0	0	0	-0.333333333	1	1	0
KY962518	79	359.1808036	0.011047049	0.001991895	0	0	0	0	0	0	0	0	0	1	1	1
KY962518	80	384.9175947	0.012062525	0.001015476	0	0	0	0	0	0	0	0	0	1	1	1
KY962518	81	219.9799107	0.005554711	-0.006507814	0	0	0	0	1	0.333333333	0	0	0.333333333	1	1	1
KY962518	82	291.9308036	0.00839362	0.002838909	0	0	0	0	1	0.333333333	0	0	0	1	1	1
KY962518	83	249.8195991	0.006732072	-0.001661548	0	0	0	0	0	0	0	0	-0.333333333	1	1	0
KY962518	84	296.7544643	0.008583943	0.001851871	0	0	0	0	0	0	0	0	0	1	1	1
KY962518	85	295.359375	0.008528898	-5.50E-05	0	0	0	0	0	0	0	0	0	1	1	1
KY962518	86	446.2293987	0.014481656	0.005952758	0	0	0	0	0	0	0	0	0	1	1	1
KY962518	87	371.7299107	0.011542189	-0.002939467	0	0	0	0	0	0	0	0	0	1	1	1
KY962518	88	457.857461	0.014940455	0.003398266	0	0	0	0	0	0	0	0	0	1	1	1
KY962518	89	262.6830357	0.007239614	-0.007700841	2	0.181818182	15	0.9375	0	0	0.181818182	0.9375	0	1	1	1
KY962518	90	413.953125	0.013208157	0.005968543	0	0	0	0	0	0	-0.181818182	-0.9375	0	1	1	1
KY962518	91	522.6325167	0.017496233	0.004288076	0	0	0	0	0	0	0	0	0	1	1	1
KY962518	92	385.2075893	0.012073967	-0.005422266	0	0	0	0	0	0	0	0	0	1	1	1
KY962518	93	250.9308036	0.006775915	-0.005298052	0	0	0	0	0	0	0	0	0	1	1	1
KY962518	94	308.8084633	0.009059548	0.002283633	0	0	0	0	0	0	0	0	0	1	1	1
KY962518	95	390.328125	0.012276004	0.003216456	0	0	0	0	0	0	0	0	0	1	1	1
KY962518	96	187.0846325	0.004256788	-0.008019216	2	0.181818182	5	0.3125	0	0	0.181818182	0.3125	0	1	1	1
KY962518	97	239.1450893	0.006310896	0.002054108	0	0	0	0	0	0	-0.181818182	-0.3125	0	1	1	1
KY962518	98	135.6026786	0.002225506	-0.00408539	0	0	0	0	0	0	0	0	0	1	1	1
KY962518	99	287.4142539	0.008215414	0.005989908	0	0	0	0	0	0	0	0	0	1	1	1
KY962518	100	161.6071429	0.003251543	-0.004963871	0	0	0	0	0	0	0	0	0	1	1	1

Supplementary Table 7. Sequence conservation of rDNA regions across species compared to human.

		Human rDNA						
Species(GenBank ID, percent identity to human rDNA)	Region	5'ETS_KY962518	18S_KY962518	ITS1_KY962518	5.8S_KY962518	ITS2_KY962518	28S_KY962518	3'ETS_KY962518
Rhesus macaque (KX061890, 88%)	5'ETS_KX061890	80.83	34.62	25.51	4.12	27.28	46.41	9.59
	18S_KX061890	34.17	99.89	38	8.08	40.16	27.89	17.08
	ITS1_KX061890	24.6	37.88	77.71	12.17	54.39	18.14	29.77
	5.8S_KX061890	4.05	8.08	11.87	100	11.48	2.91	28.25
	ITS2_KX061890	27.95	39.64	55.78	11.19	79.09	20.19	26.56
	28S_KX061890	48.33	29.47	19.31	3.18	21.09	93.7	7.11
	3'ETS_KX061890	8.70	15.73	26.59	30.82	24.59	6.26	83.7
Mouse (BK000964, 72%)	5'ETS_BK000964	54.04	32.89	22.88	3.72	24.83	46.5	8.58
	18S_BK000964	34.15	99.15	38.71	8.13	40.17	27.97	17.1
	ITS1_BK000964	23.26	36.63	55.65	13	50.45	17.28	30
	5.8S_BK000964	4.02	7.92	11.68	98.1	11.31	2.87	27.7
	ITS2_BK000964	25.49	39.27	52.68	12.04	54.71	18.77	29.69
	28S_BK000964	48.47	30.27	19.87	3.21	21.64	86.34	7.21
	3'ETS_BK000964	13.92	24.01	38.96	22.14	35.96	9.94	46.33
Chicken (KT445934, 63%)	5'ETS_KT445934	38.05	47.99	43.56	7.57	45.73	29.05	17.75
	18S_KT445934	33.13	94.44	39.12	8.23	40.84	27.31	17.83
	ITS1_KT445934	48.93	42.21	34.2	5.57	38.01	38.36	13.52
	5.8S_KT445934	4.02	8.03	11.4	98.73	11.57	2.91	28.53
	ITS2_KT445934	17.99	30.34	48.81	16.92	47.52	13.52	40.54
	28S_KT445934	49.66	30.87	21.07	3.42	22.57	78.15	7.54
	3'ETS_KT445934	8.97	15.94	27.38	28.99	25.36	6.55	55.53

Supplementary Table 8. Annotation of predicted R-loops across rDNA loci of multiple species

Species	rDNA_region	RLFS_start	RLFS_end	RLFS_length	RLFS_name	strand
Rhesus macaque	5'ETS	475	613	138	KX061890.138.475.m1	+
	5'ETS	582	1259	677	KX061890.677.582.m1	+
	5'ETS	1353	3419	2066	KX061890.2066.1353.m1	+
	5'ETS	1545	3542	1997	KX061890.1997.1545.m1	+
	5'ETS	1894	3526	1632	KX061890.1632.1894.m1	+
	5'ETS	1984	3491	1507	KX061890.1507.1984.m1	+
	5'ETS	2408	3622	1214	KX061890.1214.2408.m1	+
	5'ETS	2447	3542	1095	KX061890.1095.2447.m1	+
	5'ETS	2487	3509	1022	KX061890.1022.2487.m1	+
	5'ETS	2965	3561	596	KX061890.596.2965.m1	+
	5'ETS	3023	3588	565	KX061890.565.3023.m1	+
	5'ETS	3111	3561	450	KX061890.450.3111.m1	+
	5'ETS	3243	3526	283	KX061890.283.3243.m1	+
	5'ETS	3370	3511	141	KX061890.141.3370.m1	+
	ITS1	5720	7730	2010	KX061890.2010.5720.m1	+
	ITS1	5767	6399	632	KX061890.632.5767.m1	+
	ITS1	6086	7820	1734	KX061890.1734.6086.m1	+
	ITS1	6113	7823	1710	KX061890.1710.6113.m1	+
	ITS1	6316	7245	929	KX061890.929.6316.m1	+
	ITS2	6709	8179	1470	KX061890.1470.6709.m1	+
	ITS2	6743	8178	1435	KX061890.1435.6743.m1	+
	ITS2	6862	8185	1323	KX061890.1323.6862.m1	+
	ITS2	6998	8056	1058	KX061890.1058.6998.m1	+
	ITS2	7115	7833	718	KX061890.718.7115.m1	+
	ITS2	7149	7728	579	KX061890.579.7149.m1	+
	ITS2	7520	7848	328	KX061890.328.7520.m1	+
	ITS2	7590	7832	242	KX061890.242.7590.m1	+
	28S	8448	9447	999	KX061890.999.8448.m1	+
	28S	8470	9420	950	KX061890.950.8470.m1	+
	28S	8484	9222	738	KX061890.738.8484.m1	+
	28S	8581	9222	641	KX061890.641.8581.m1	+
	28S	8664	9108	444	KX061890.444.8664.m1	+
	28S	8814	9285	471	KX061890.471.8814.m1	+
	28S	8933	9374	441	KX061890.441.8933.m1	+
	28S	8981	9244	263	KX061890.263.8981.m1	+
	28S	9004	9213	209	KX061890.209.9004.m1	+
	28S	10029	11237	1208	KX061890.1208.10029.m1	+
	28S	10611	11383	772	KX061890.772.10611.m1	+
	28S	10630	11361	731	KX061890.731.10630.m1	+
	28S	10781	11442	661	KX061890.661.10781.m1	+
	28S	10858	11360	502	KX061890.502.10858.m1	+
	28S	11021	11246	225	KX061890.225.11021.m1	+
	28S	11054	11241	187	KX061890.187.11054.m1	+
	28S	11603	11876	273	KX061890.273.11603.m1	+
	28S	11687	11876	189	KX061890.189.11687.m1	+
	3'ETS	12747	12978	231	KX061890.231.12747.m1	+
	3'ETS	12838	12978	140	KX061890.140.12838.m1	+
	3'ETS	12598	12926	328	KX061890.328.12598.m1	-
	28S	10482	11156	674	KX061890.674.10482.m1	-
	28S	10483	11092	609	KX061890.609.10483.m1	-
	28S	10560	10937	377	KX061890.377.10560.m1	-
	28S	10556	10877	321	KX061890.321.10556.m1	-
	28S	10529	10781	252	KX061890.252.10529.m1	-
	28S	10559	10761	202	KX061890.202.10559.m1	-
	28S	10600	10732	132	KX061890.132.10600.m1	-
	28S	7158	9102	1944	KX061890.1944.7158.m1	-
	28S	7142	8927	1785	KX061890.1785.7142.m1	-
	28S	7169	8842	1673	KX061890.1673.7169.m1	-
	28S	7190	8806	1616	KX061890.1616.7190.m1	-
	28S	8276	8664	388	KX061890.388.8276.m1	-
	28S	7190	8637	1447	KX061890.1447.7190.m1	-
	28S	8268	8445	177	KX061890.177.8268.m1	-
	28S	8283	8417	134	KX061890.134.8283.m1	-
	28S	6610	7994	1384	KX061890.1384.6610.m1	-
	ITS2	5838	7856	2018	KX061890.2018.5838.m1	-
	ITS2	5824	7837	2013	KX061890.2013.5824.m1	-
	ITS2	5789	7768	1979	KX061890.1979.5789.m1	-
	ITS2	6608	7694	1086	KX061890.1086.6608.m1	-
	ITS2	5784	7677	1893	KX061890.1893.5784.m1	-
	ITS2	6751	7374	623	KX061890.623.6751.m1	-
	ITS2	7127	7326	199	KX061890.199.7127.m1	-

	ITS2	5804	6849	1045	KX061890.1045.5804.m1	-
	ITS2	6624	6797	173	KX061890.173.6624.m1	-
	ITS1	5694	6162	468	KX061890.468.5694.m1	-
	ITS1	5694	6011	317	KX061890.317.5694.m1	-
	18S	2590	3900	1310	KX061890.1310.2590.m1	-
	5'ETS	2084	3567	1483	KX061890.1483.2084.m1	-
	5'ETS	2501	3519	1018	KX061890.1018.2501.m1	-
	5'ETS	2516	3498	982	KX061890.982.2516.m1	-
	5'ETS	2503	3105	602	KX061890.602.2503.m1	-
	5'ETS	2506	3018	512	KX061890.512.2506.m1	-
	5'ETS	2506	2996	490	KX061890.490.2506.m1	-
	5'ETS	2495	2898	403	KX061890.403.2495.m1	-
	5'ETS	2019	2302	283	KX061890.283.2019.m1	-
	5'ETS	1216	1340	124	KX061890.124.1216.m1	-
	5'ETS	528	916	388	KX061890.388.528.m1	-
	5'ETS	528	793	265	KX061890.265.528.m1	-
	5'ETS	1824	3542	1718	KX061890.1718.1824.m2	+
	5'ETS	1991	3491	1500	KX061890.1500.1991.m2	+
	5'ETS	2810	3588	778	KX061890.778.2810.m2	+
	5'ETS	2970	3561	591	KX061890.591.2970.m2	+
	5'ETS	3243	3542	299	KX061890.299.3243.m2	+
	5'ETS	3370	3526	156	KX061890.156.3370.m2	+
	18S	5241	6381	1140	KX061890.1140.5241.m2	+
	ITS1	5720	7730	2010	KX061890.2010.5720.m2	+
	ITS2	6730	8178	1448	KX061890.1448.6730.m2	+
	ITS2	6866	8185	1319	KX061890.1319.6866.m2	+
	ITS2	7041	7938	897	KX061890.897.7041.m2	+
	ITS2	7115	7855	740	KX061890.740.7115.m2	+
	ITS2	7162	7728	566	KX061890.566.7162.m2	+
	ITS2	7590	7832	242	KX061890.242.7590.m2	+
	28S	8470	9384	914	KX061890.914.8470.m2	+
	28S	8490	9222	732	KX061890.732.8490.m2	+
	28S	8603	9304	701	KX061890.701.8603.m2	+
	28S	8814	9374	560	KX061890.560.8814.m2	+
	28S	8949	9374	425	KX061890.425.8949.m2	+
	28S	9004	9213	209	KX061890.209.9004.m2	+
	28S	10630	11368	738	KX061890.738.10630.m2	+
	28S	10781	11445	664	KX061890.664.10781.m2	+
	28S	10826	11383	557	KX061890.557.10826.m2	+
	28S	11021	11294	273	KX061890.273.11021.m2	+
	28S	11603	11853	250	KX061890.250.11603.m2	+
	28S	11687	11876	189	KX061890.189.11687.m2	+
	3'ETS	12747	12978	231	KX061890.231.12747.m2	+
	28S	10482	11156	674	KX061890.674.10482.m2	-
	28S	10483	11092	609	KX061890.609.10483.m2	-
	28S	10480	10781	301	KX061890.301.10480.m2	-
	28S	10587	10732	145	KX061890.145.10587.m2	-
	28S	6950	8981	2031	KX061890.2031.6950.m2	-
	28S	7135	8927	1792	KX061890.1792.7135.m2	-
	28S	7167	8842	1675	KX061890.1675.7167.m2	-
	28S	7182	8445	1263	KX061890.1263.7182.m2	-
	28S	6615	7994	1379	KX061890.1379.6615.m2	-
	ITS2	5789	7677	1888	KX061890.1888.5789.m2	-
	ITS2	5784	6849	1065	KX061890.1065.5784.m2	-
	ITS1	5694	6011	317	KX061890.317.5694.m2	-
	18S	2590	3900	1310	KX061890.1310.2590.m2	-
	5'ETS	2490	3519	1029	KX061890.1029.2490.m2	-
	5'ETS	2506	3506	1000	KX061890.1000.2506.m2	-
	5'ETS	2506	3018	512	KX061890.512.2506.m2	-
	5'ETS	528	916	388	KX061890.388.528.m2	-
Species	rDNA_region	RLFS_start	RLFS_end	RLFS_length	RLFS_name	strand
Mouse	5'ETS	1705	1894	189	BK000964.189.6705.m1	+
	5'ETS	2153	3012	859	BK000964.859.7153.m1	+
	5'ETS	2642	3119	477	BK000964.477.7642.m1	+
	5'ETS	3705	3917	212	BK000964.212.8705.m1	+
	ITS1	6453	6676	223	BK000964.223.11453.m1	+
	ITS2	7509	7969	460	BK000964.460.12509.m1	+
	28S	8748	9236	488	BK000964.488.13748.m1	+
	28S	9026	9370	344	BK000964.344.14026.m1	+
	28S	9104	9240	136	BK000964.136.14104.m1	+
	28S	9334	9564	230	BK000964.230.14334.m1	+
	28S	10024	10656	632	BK000964.632.15024.m1	+
	28S	10056	10637	581	BK000964.581.15056.m1	+

	28S	10190	10601	411	BK000964.411.15190.m1	+
	28S	10770	11416	646	BK000964.646.15770.m1	+
	28S	10789	11338	549	BK000964.549.15789.m1	+
	28S	10964	11536	572	BK000964.572.15964.m1	+
	28S	10995	11492	497	BK000964.497.15995.m1	+
	28S	11171	11416	245	BK000964.245.16171.m1	+
	28S	11860	12051	191	BK000964.191.16860.m1	+
	28S	12613	12732	119	BK000964.119.17613.m1	+
	3'ETS	13214	13394	180	BK000964.180.18214.m1	+
	28S	10754	11243	489	BK000964.489.15754.m1	-
	28S	10758	10924	166	BK000964.166.15758.m1	-
	28S	10773	10896	123	BK000964.123.15773.m1	-
	28S	8549	9251	702	BK000964.702.13549.m1	-
	28S	8566	9026	460	BK000964.460.13566.m1	-
	28S	8541	8696	155	BK000964.155.13541.m1	-
	ITS2	7658	7992	334	BK000964.334.12658.m1	-
	ITS2	6951	7354	403	BK000964.403.11951.m1	-
	ITS2	7039	7258	219	BK000964.219.12039.m1	-
	ITS1	6317	6452	135	BK000964.135.11317.m1	-
	ITS1	6022	6254	232	BK000964.232.11022.m1	-
	5'ETS	2962	3671	709	BK000964.709.7962.m1	-
	5'ETS	2946	3537	591	BK000964.591.7946.m1	-
	5'ETS	2922	3382	460	BK000964.460.7922.m1	-
	5'ETS	1799	2056	257	BK000964.257.6799.m1	-
	5'ETS	2653	3119	466	BK000964.466.7653.m2	+
	5'ETS	3724	3892	168	BK000964.168.8724.m2	+
	ITS1	6458	6676	218	BK000964.218.11458.m2	+
	ITS2	7443	8033	590	BK000964.590.12443.m2	+
	ITS2	7521	7969	448	BK000964.448.12521.m2	+
	ITS2	7721	7963	242	BK000964.242.12721.m2	+
	28S	8752	9236	484	BK000964.484.13752.m2	+
	28S	8764	9055	291	BK000964.291.13764.m2	+
	28S	9036	9370	334	BK000964.334.14036.m2	+
	28S	9104	9247	143	BK000964.143.14104.m2	+
	28S	10056	10601	545	BK000964.545.15056.m2	+
	28S	10789	11352	563	BK000964.563.15789.m2	+
	28S	10964	11536	572	BK000964.572.15964.m2	+
	28S	11061	11416	355	BK000964.355.16061.m2	+
	28S	11781	12051	270	BK000964.270.16781.m2	+
	28S	11860	12051	191	BK000964.191.16860.m2	+
	3'ETS	13214	13394	180	BK000964.180.18214.m2	+
	IGS	13232	13559	327	BK000964.327.18232.m2	-
	28S	10763	11329	566	BK000964.566.15763.m2	-
	28S	10764	11208	444	BK000964.444.15764.m2	-
	28S	10746	10924	178	BK000964.178.15746.m2	-
	28S	10762	10904	142	BK000964.142.15762.m2	-
	28S	8534	9147	613	BK000964.613.13534.m2	-
	28S	8534	8696	162	BK000964.162.13534.m2	-
	ITS2	7658	7988	330	BK000964.330.12658.m2	-
	5'ETS	2964	3524	560	BK000964.560.7964.m2	-
	5'ETS	2384	2535	151	BK000964.151.7384.m2	-

Species	rDNA_region	RLFS_start	RLFS_end	RLFS_length	RLFS_name	strand
Chicken	5'ETS	291	599	308	KT445934.308.291.m1	+
	5'ETS	1102	1932	830	KT445934.830.1102.m1	+
	5'ETS	1243	1872	629	KT445934.629.1243.m1	+
	5'ETS	1582	1918	336	KT445934.336.1582.m1	+
	ITS1	3865	5887	2022	KT445934.2022.3865.m1	+
	ITS1	4062	6077	2015	KT445934.2015.4062.m1	+
	ITS1	4214	6236	2022	KT445934.2022.4214.m1	+
	ITS1	4309	6324	2015	KT445934.2015.4309.m1	+
	ITS1	4573	6613	2040	KT445934.2040.4573.m1	+
	ITS1	4638	6682	2044	KT445934.2044.4638.m1	+
	ITS1	5010	7066	2056	KT445934.2056.5010.m1	+
	ITS2	7021	8631	1610	KT445934.1610.7021.m1	+
	28S	7628	8977	1349	KT445934.1349.7628.m1	+
	28S	7645	8845	1200	KT445934.1200.7645.m1	+
	28S	7737	8769	1032	KT445934.1032.7737.m1	+
	28S	7993	8761	768	KT445934.768.7993.m1	+
	28S	8017	8712	695	KT445934.695.8017.m1	+
	28S	8089	8643	554	KT445934.554.8089.m1	+
	28S	8156	8631	475	KT445934.475.8156.m1	+
	28S	8254	8631	377	KT445934.377.8254.m1	+
	28S	9140	10194	1054	KT445934.1054.9140.m1	+

		28S	9723	10322	599	KT445934.599.9723.m1	+
		28S	9742	10321	579	KT445934.579.9742.m1	+
		28S	9827	10261	434	KT445934.434.9827.m1	+
		28S	10615	10799	184	KT445934.184.10615.m1	+
		3'ETS	11566	11861	295	KT445934.295.11566.m1	+
		3'ETS	11677	11862	185	KT445934.185.11677.m1	+
		3'ETS	11639	11786	147	KT445934.147.11639.m1	-
		3'ETS	11496	11741	245	KT445934.245.11496.m1	-
		28S	9591	10019	428	KT445934.428.9591.m1	-
		28S	9673	9975	302	KT445934.302.9673.m1	-
		28S	9717	9957	240	KT445934.240.9717.m1	-
		28S	9726	9909	183	KT445934.183.9726.m1	-
		28S	7467	8319	852	KT445934.852.7467.m1	-
		28S	7477	8004	527	KT445934.527.7477.m1	-
		28S	6278	7210	932	KT445934.932.6278.m1	-
		ITS2	5046	7070	2024	KT445934.2024.5046.m1	-
		ITS2	4737	6751	2014	KT445934.2014.4737.m1	-
		ITS2	4591	6612	2021	KT445934.2021.4591.m1	-
		ITS2	4537	6521	1984	KT445934.1984.4537.m1	-
		ITS1	3522	5535	2013	KT445934.2013.3522.m1	-
		ITS1	3522	5323	1801	KT445934.1801.3522.m1	-
		ITS1	3630	5177	1547	KT445934.1547.3630.m1	-
		ITS1	3671	4970	1299	KT445934.1299.3671.m1	-
		ITS1	3665	4801	1136	KT445934.1136.3665.m1	-
		ITS1	3671	4631	960	KT445934.960.3671.m1	-
		ITS1	3652	4304	652	KT445934.652.3652.m1	-
		5'ETS	402	971	569	KT445934.569.402.m1	-
		5'ETS	407	709	302	KT445934.302.407.m1	-
		5'ETS	81	580	499	KT445934.499.81.m2	+
		5'ETS	1243	1883	640	KT445934.640.1243.m2	+
		5'ETS	1582	1931	349	KT445934.349.1582.m2	+
		18S	3393	5356	1963	KT445934.1963.3393.m2	+
		ITS1	3865	5887	2022	KT445934.2022.3865.m2	+
		ITS1	4062	6072	2010	KT445934.2010.4062.m2	+
		ITS1	4309	6319	2010	KT445934.2010.4309.m2	+
		ITS1	4573	6585	2012	KT445934.2012.4573.m2	+
		ITS1	4638	6682	2044	KT445934.2044.4638.m2	+
		ITS1	4729	6701	1972	KT445934.1972.4729.m2	+
		ITS1	4863	6544	1681	KT445934.1681.4863.m2	+
		ITS1	5604	7104	1500	KT445934.1500.5604.m2	+
		ITS1	5813	5951	138	KT445934.138.5813.m2	+
		28S	7628	9173	1545	KT445934.1545.7628.m2	+
		28S	7645	8907	1262	KT445934.1262.7645.m2	+
		28S	8004	8761	757	KT445934.757.8004.m2	+
		28S	8101	8643	542	KT445934.542.8101.m2	+
		28S	8156	8635	479	KT445934.479.8156.m2	+
		28S	9742	10281	539	KT445934.539.9742.m2	+
		28S	9827	10270	443	KT445934.443.9827.m2	+
		28S	10066	10261	195	KT445934.195.10066.m2	+
		28S	10615	10799	184	KT445934.184.10615.m2	+
		3'ETS	11677	11862	185	KT445934.185.11677.m2	+
		3'ETS	11698	11861	163	KT445934.163.11698.m2	+
		3'ETS	11496	11741	245	KT445934.245.11496.m2	-
		3'ETS	11496	11661	165	KT445934.165.11496.m2	-
		28S	9687	9971	284	KT445934.284.9687.m2	-
		28S	9717	9946	229	KT445934.229.9717.m2	-
		28S	7477	7993	516	KT445934.516.7477.m2	-
		28S	7515	7902	387	KT445934.387.7515.m2	-
		28S	6293	7254	961	KT445934.961.6293.m2	-
		28S	6278	7210	932	KT445934.932.6278.m2	-
		ITS2	4741	6751	2010	KT445934.2010.4741.m2	-
		ITS2	4689	6721	2032	KT445934.2032.4689.m2	-
		ITS2	4537	6521	1984	KT445934.1984.4537.m2	-
		ITS1	4072	6087	2015	KT445934.2015.4072.m2	-
		ITS1	3654	4801	1147	KT445934.1147.3654.m2	-
		ITS1	3671	4631	960	KT445934.960.3671.m2	-
		ITS1	3641	4509	868	KT445934.868.3641.m2	-
		ITS1	3679	4292	613	KT445934.613.3679.m2	-
		5'ETS	1186	1580	394	KT445934.394.1186.m2	-
		5'ETS	407	701	294	KT445934.294.407.m2	-

Supplementary Table 9. Annotation of predicted G-quadruplex forming sequences (G4FS) across rDNA loci of multiple species

Species	rDNA_region	G4FS_start	G4FS_end	G4FS_length	sequence	name	strand
Rhesus macaque	5'ETS	475	499	24	CGGGACCGGGCCGCG	KX061890000000475	+
	5'ETS	480	506	26	CGGGCCGCGACGGGC	KX061890000000480	+
	5'ETS	486	510	24	CGCGACGGGCGAGGG	KX061890000000486	+
	ITS1	5767	5784	17	GGCGCGGGGGGGAG	KX061890000005767	+
	ITS1	6316	6346	30	GGAACCCCCGGGCGC	KX061890000006316	+
	ITS1	6317	6346	29	GAACCCCCGGGCGC	KX061890000006317	+
	ITS1	6318	6346	28	GAACCCCCGGGCGCC	KX061890000006318	+
	ITS2	6862	6893	31	GGGGGAGGGGGGGCC	KX061890000006862	+
	ITS2	6866	6893	27	GGGAGGGGGGGCCC	KX061890000006866	+
	ITS2	6867	6893	26	GGGAGGGGGGGCCCG	KX061890000006867	+
	ITS2	6868	6893	25	GGAGGGGGGGCCCG	KX061890000006868	+
	ITS2	6869	6893	24	GGAGGGGGGGCCCGG	KX061890000006869	+
	ITS2	6870	6893	23	GAGGGGGGGCCCGG	KX061890000006870	+
	ITS2	6871	6893	22	GAGGGGGGGCCCGGC	KX061890000006871	+
	ITS2	6872	6893	21	AGGGGGGGCCCGGC	KX061890000006872	+
	ITS2	7130	7152	22	GGTGGGGGGGAAGGA	KX061890000007130	+
	ITS2	7131	7152	21	GTGGGGGGGAAGGAA	KX061890000007131	+
	ITS2	7132	7152	20	GTGGGGGGGAAGGAA	KX061890000007132	+
	ITS2	7590	7623	33	GGGACGGGTCGGGG	KX061890000007590	+
	ITS2	7591	7623	32	GGGACGGGTCGGGGG	KX061890000007591	+
	ITS2	7592	7623	31	GGACGGGTCGGGGG	KX061890000007592	+
	ITS2	7593	7623	30	GGACGGGTCGGGGGG	KX061890000007593	+
	ITS2	7594	7623	29	GACGGGTCGGGGGG	KX061890000007594	+
	ITS2	7595	7623	28	GACGGGTCGGGGGGT	KX061890000007595	+
	ITS2	7596	7623	27	ACGGGTCGGGGGGTC	KX061890000007596	+
	ITS2	7597	7623	26	ACGGGTCGGGGGGTC	KX061890000007597	+
	ITS2	7598	7623	25	CGGGTCGGGGGGTCG	KX061890000007598	+
	ITS2	7599	7623	24	CGGGTCGGGGGGTCG	KX061890000007599	+
	ITS2	7604	7629	25	GGGGGGTCGGTGGGC	KX061890000007604	+
	28S	8448	8475	27	GGGCTCCGGCGGGTG	KX061890000008448	+
	28S	8449	8475	26	GGGCTCCGGCGGGTG	KX061890000008449	+
	28S	8450	8475	25	GGCTCCGGCGGGTGC	KX061890000008450	+
	28S	8454	8479	25	CCGGCGGGTGCGGGG	KX061890000008454	+
	28S	8464	8483	19	GTGCGGGGGTGGGCG	KX061890000008464	+
	28S	8470	8488	18	GGGGTGGGCGGGCGG	KX061890000008470	+
	28S	8471	8488	17	GGGTGGGCGGGCGGG	KX061890000008471	+
	28S	8472	8488	16	GGGTGGGCGGGCGGG	KX061890000008472	+
	28S	8476	8495	19	GCGGGCGGGGCCGGG	KX061890000008476	+
	28S	8480	8500	20	GCGGGGCCGGGGGTG	KX061890000008480	+
	28S	8484	8510	26	CGGGGGTGGGGTCGG	KX061890000008484	+
	28S	8485	8510	25	GGGGGTGGGGTCGGC	KX061890000008485	+
	28S	8664	8683	19	GAGGGCGCGGGTCGG	KX061890000008664	+
	28S	8933	8959	26	CCCGCGCGGGTGGGG	KX061890000008933	+
	28S	8934	8959	25	CCGCGCGGGTGGGGG	KX061890000008934	+
	28S	8935	8959	24	CCGCGCGGGTGGGGG	KX061890000008935	+
	28S	8998	9022	24	CCGGGGGGGTTCTCT	KX061890000008998	+
	28S	10611	10634	23	TGGGTCGGTCGGGCT	KX061890000010611	+
	28S	10781	10805	24	GTGCGGGGGGAAGGG	KX061890000010781	+
	28S	10782	10805	23	TGCGGGGGGAAGGGT	KX061890000010782	+
	28S	10783	10805	22	TGCGGGGGGAAGGGT	KX061890000010783	+
	28S	10789	10813	24	GGAAGGGTCGGGCGG	KX061890000010789	+
	28S	10790	10813	23	GAAGGGTCGGGCGGA	KX061890000010790	+
	28S	10791	10813	22	GAAGGGTCGGGCGGA	KX061890000010791	+
	28S	10792	10813	21	AAGGGTCGGGCGGAG	KX061890000010792	+
	28S	10858	10883	25	GGGCCCGGGCACCCC	KX061890000010858	+
	28S	10859	10883	24	GGGCCCGGGCACCCG	KX061890000010859	+
	5'ETS	1316	1340	24	CGCCTGGGGAAGGGA	KX061890000001316	-
	5'ETS	1317	1340	23	CGCCTGGGGAAGGGA	KX061890000001317	-
	5'ETS	1318	1340	22	ACGCCTGGGGAAGGG	KX061890000001318	-
	5'ETS	2633	2654	21	GAGGGGGGGCGCCGG	KX061890000002633	-
	5'ETS	2634	2654	20	GGAGGGGGGGCGCCG	KX061890000002634	-
	5'ETS	2635	2654	19	GGAGGGGGGGCGCCG	KX061890000002635	-
	5'ETS	2981	3004	23	CCGAGGGCCCCGGGC	KX061890000002981	-
	5'ETS	2986	3010	24	CCGGGGCCGAGGGCC	KX061890000002986	-
	5'ETS	2993	3018	25	ACCGGGACCGGGGCC	KX061890000002993	-
	5'ETS	3494	3519	25	GATGGGGCGGGGGCG	KX061890000003494	-
	5'ETS	3495	3519	24	GGATGGGGCGGGGGC	KX061890000003495	-
	ITS2	7355	7374	19	GCGGGCCGCGGGGCG	KX061890000007355	-
	ITS2	7673	7694	21	GACGACGGGAGGGAG	KX061890000007673	-
	ITS2	7674	7694	20	GGACGACGGGAGGGA	KX061890000007674	-
	ITS2	7824	7841	17	GGCGGGGGTGGGCGG	KX061890000007824	-
	28S	8377	8402	25	CGGGAGGAACGGGGG	KX061890000008377	-
	28S	8381	8409	28	AGGGGTCGGGAGGAA	KX061890000008381	-
	28S	8382	8409	27	AGGGGTCGGGAGGAA	KX061890000008382	-
	28S	8383	8409	26	GAGGGGTCGGGAGGA	KX061890000008383	-
	28S	8384	8409	25	GAGGGGTCGGGAGGA	KX061890000008384	-
	28S	8393	8413	20	GCGGGTGGAGGGGTC	KX061890000008393	-
	28S	8398	8417	19	GAGGGCGGGTGGAGG	KX061890000008398	-
	28S	8399	8417	18	GGAGGGCGGGTGGAG	KX061890000008399	-
	28S	8406	8423	17	GGGGAGGGAGGGCGG	KX061890000008406	-
	28S	8410	8431	21	GCGGCGGGGGAGGGA	KX061890000008410	-
	28S	8623	8648	25	GAAGACGGGACGGGA	KX061890000008623	-
	28S	8624	8648	24	GGAAGACGGGACGGG	KX061890000008624	-
	28S	8629	8652	23	GGGGGGAAGACGGGA	KX061890000008629	-
	28S	8634	8664	30	GACGCGGGCGGGGGG	KX061890000008634	-
	28S	8789	8812	23	GTGGGCGCCGGGAGG	KX061890000008789	-
	28S	8790	8812	22	GGTGGGCGCCGGGAG	KX061890000008790	-
	28S	8791	8812	21	GGTGGGCGCCGGGAG	KX061890000008791	-
	28S	8792	8812	20	GGGTGGGCGCCGGGA	KX061890000008792	-
	28S	8796	8825	29	CCCCGCGGGGGTGGG	KX061890000008796	-
	28S	8905	8927	22	GGTGCGGGGAGGGGG	KX061890000008905	-
	28S	9948	9974	26	TCCGGGGGCGGGCGG	KX061890000009948	-
	28S	9949	9974	25	TCCGGGGGCGGGCGG	KX061890000009949	-
	28S	9950	9974	24	CTCCGGGGGCGGGCG	KX061890000009950	-
	28S	9951	9974	23	CTCCGGGGGCGGGCG	KX061890000009951	-
	28S	9952	9974	22	GCTCCGGGGGCGGGC	KX061890000009952	-
	28S	9953	9974	21	GCTCCGGGGGCGGGC	KX061890000009953	-
	28S	10681	10716	35	GGGGGTGCCCCGGGC	KX061890000010681	-
	28S	10682	10716	34	GGGGGGTGCCCCGGG	KX061890000010682	-
	28S	10683	10716	33	GGGGGGTGCCCCGGC	KX061890000010683	-
	28S	10684	10716	32	AGGGGGGTGCCCCGG	KX061890000010684	-
	28S	10685	10716	31	AGGGGGGTGCCCCGG	KX061890000010685	-
	28S	10686	10716	30	GAGGGGGGTGCCCCG	KX061890000010686	-
	28S	10692	10722	30	GGCCGCGAGGGGGGT	KX061890000010692	-
	28S	10701	10727	26	GGGGGAGGGCCGCGA	KX061890000010701	-
	28S	10702	10727	25	GGGGGAGGGCCGCGA	KX061890000010702	-
	28S	10703	10727	24	CGGGGGAGGGCCGCG	KX061890000010703	-
	28S	10704	10727	23	CGGGGGAGGGCCGCC	KX061890000010704	-
	28S	10713	10732	19	GGTGGGGCGGGGGAG	KX061890000010713	-
	28S	10746	10771	25	AGAGAGGGCGCGGGG	KX061890000010746	-
	28S	10750	10776	26	GGGGGAGAGAGGGCGG	KX061890000010750	-

	28S	10751	10776	25	GGGGGAGAGAGGGCG	KX061890000010751	-
	28S	10752	10776	24	AGGGGGAGAGAGGGC	KX061890000010752	-
	28S	10758	10781	23	AGGGGAGGGGGAGAG	KX061890000010758	-

Species	rDNA_region	G4FS_start	G4FS_end	G4FS_length	sequence	name	strand
Mouse	5'ETS	2653	2678	25	CTTGGGGGGGGGCCG	G4CBK000964000007653	+
	5'ETS	2654	2678	24	TTGGGGGGGGGCCG	G4CBK000964000007654	+
	ITS1	6008	6031	23	TGGGTGGGGTCGGTC	G4CBK000964000011008	+
	ITS1	6009	6031	22	TGGGTGGGGTCGGTC	G4CBK000964000011009	+
	ITS1	6013	6041	28	GGTCGGTCTGGGTCC	G4CBK000964000011013	+
	ITS1	6453	6481	28	GGGGCGGTGGGGCC	G4CBK000964000011453	+
	28S	8748	8768	20	CGGGGCCGGGGGTG	G4CBK000964000013748	+
	28S	8752	8778	26	GGGGGTGGGGTCGG	G4CBK000964000013752	+
	28S	8753	8778	25	GGGGGTGGGGTCGGC	G4CBK000964000013753	+
	28S	9026	9047	21	CGGGCGTGGGGGTGG	G4CBK000964000014026	+
	28S	9030	9052	22	GTGGGGGTGGGGGC	G4CBK000964000014030	+
	28S	10770	10793	23	TGGGTCGGTCGGGCT	G4CBK000964000015770	+
	5'ETS	1885	1908	23	AGACGGGTGGGGACC	G4CBK000964000006885	-
	5'ETS	3356	3382	26	GGCAGGGGCTCACG	G4CBK000964000008356	-
	5'ETS	3509	3537	28	GCAGGCGGGGGTCG	G4CBK000964000008509	-
	5'ETS	3510	3537	27	GGCAGGCGGGGGTCG	G4CBK000964000008510	-
	5'ETS	3511	3537	26	GGCAGGCGGGGGTC	G4CBK000964000008511	-
	5'ETS	3635	3666	31	AGGGTGGTGGGTTGG	G4CBK000964000008635	-
	5'ETS	3636	3666	30	CAGGGTGGTGGGTTG	G4CBK000964000008636	-
	5'ETS	3637	3666	29	CAGGGTGGTGGGTTG	G4CBK000964000008637	-
	5'ETS	3647	3671	24	CGGGAGAGCAGGGTG	G4CBK000964000008647	-
	ITS1	6055	6076	21	AGAGGGGAGGGGGAG	G4CBK000964000011055	-
	ITS1	6056	6076	20	GAGAGGGGAGGGGGA	G4CBK000964000011056	-
	ITS2	7236	7258	22	GTGCACGGGCGAGGG	G4CBK000964000012236	-
	ITS2	7314	7345	31	GGTGGGTTCCCACGG	G4CBK000964000012314	-
	ITS2	7320	7354	34	GGGGGCGCGGTGGGT	G4CBK000964000012320	-
	ITS2	7321	7354	33	GGGGGCGCGGTGGG	G4CBK000964000012321	-
	ITS2	7971	7992	21	AGGGGAGGGCGGCGG	G4CBK000964000012971	-
	ITS2	7972	7992	20	GAGGGGAGGGCGGCG	G4CBK000964000012972	-
	ITS2	7973	7992	19	GAGGGGAGGGCGGCG	G4CBK000964000012973	-
	28S	8639	8664	25	CGGGAGGAACGGGGA	G4CBK000964000013639	-
	28S	8645	8671	26	GAGGGGTCGGGAGGA	G4CBK000964000013645	-
	28S	8646	8671	25	GAGGGGTCGGGAGGA	G4CBK000964000013646	-
	28S	10878	10896	18	GGAGGGCGGGCCCG	G4CBK000964000015878	-
	28S	10884	10904	20	GGAAGAGGGGAGGGC	G4CBK000964000015884	-
	28S	10888	10914	26	CCCGCGGGGAAGAGG	G4CBK000964000015888	-
	28S	10892	10924	32	CGGGGCCCCGCGGG	G4CBK000964000015892	-
	28S	10893	10924	31	ACGGGGCCCCGCGG	G4CBK000964000015893	-
	3'ETS	13289	13313	24	CGGGAGTGGGGATGC	G4CBK000964000018289	-

Species	rDNA_region	G4FS_start	G4FS_end	G4FS_length	sequence	name	strand
Chicken	ITS1	4309	4331	22	GACGGGGCCGGGTCG	G4CKT445934000004309	+
	ITS1	4310	4331	21	ACGGGGCCGGGTCGC	G4CKT445934000004310	+
	ITS1	4482	4512	30	GAGGGAAACCCCGG	G4CKT445934000004482	+
	ITS1	5010	5036	26	GGCCCGGGCCGGAG	G4CKT445934000005010	+
	ITS1	5011	5036	25	GGCCCGGGCCGGAG	G4CKT445934000005011	+
	ITS2	7021	7053	32	GGCGAGCGCGGGCT	G4CKT445934000007021	+
	28S	7628	7652	24	GGGGCGGGCCCAGG	G4CKT445934000007628	+
	28S	7629	7652	23	GGGGCGGGCCCAGG	G4CKT445934000007629	+
	28S	7633	7656	23	CGGGCCCAGGGGGG	G4CKT445934000007633	+
	28S	7634	7656	22	GGGCCCAGGGGGGG	G4CKT445934000007634	+
	28S	7638	7660	22	CCAGGGGGGGCGGG	G4CKT445934000007638	+
	28S	7645	7666	21	GGGGCGGGCGGGCCC	G4CKT445934000007645	+
	28S	7646	7666	20	GGGCGGGCGGGCCG	G4CKT445934000007646	+
	28S	7647	7666	19	GGGCGGGCGGGCCGG	G4CKT445934000007647	+
	28S	7648	7666	18	GGCGGGCGGGCCGG	G4CKT445934000007648	+
	28S	7649	7666	17	GGCGGGCGGGCCGGC	G4CKT445934000007649	+
	28S	8004	8030	26	CGGGGGCGGGGGGG	G4CKT445934000008004	+
	28S	8005	8030	25	CGGGGGCGGGGGGG	G4CKT445934000008005	+
	28S	8011	8030	19	GGGCGGGGGGGGTCG	G4CKT445934000008011	+
	28S	8012	8030	18	GGCGGGGGGGGTCG	G4CKT445934000008012	+
	28S	8013	8030	17	GGCGGGGGGGGTCGG	G4CKT445934000008013	+
	28S	9723	9746	23	TGGGTCGGTCGGGCT	G4CKT445934000009723	+
	28S	9827	9854	27	CCGGGGGGGGGGGTC	G4CKT445934000009827	+
	28S	9828	9854	26	CGGGGGGGGGGGTC	G4CKT445934000009828	+
	28S	9835	9854	19	GGGGGGGGGTCAGCG	G4CKT445934000009835	+
	28S	10090	10117	27	GGCGGTCCCGGGCGG	G4CKT445934000010090	+
	28S	10094	10125	31	CCCGGGCGGGGGGG	G4CKT445934000010094	+
	28S	10095	10125	30	CCGGGCGGGGGGGG	G4CKT445934000010095	+
	28S	10105	10125	20	CGGGGGGGGTCTCC	G4CKT445934000010105	+
	5'ETS	688	709	21	AGAAAGGGGGTGGGG	G4CKT445934000000688	-
	ITS1	3721	3741	20	GCTCGGGGAAGGGAA	G4CKT445934000003721	-
	ITS1	4268	4299	31	GGGGTGGGGGGTGAG	G4CKT445934000004268	-
	ITS1	4269	4299	30	GGGGTGGGGGGTGA	G4CKT445934000004269	-
	ITS1	4270	4299	29	GGGGGTGGGGGGTGA	G4CKT445934000004270	-
	ITS1	4280	4304	24	GGGCGAAGGGGGTG	G4CKT445934000004280	-
	ITS1	4281	4304	23	CGGGCGAAGGGGGTG	G4CKT445934000004281	-
	ITS1	4282	4304	22	CGGGCGAAGGGGGT	G4CKT445934000004282	-
	ITS1	4283	4304	21	CCGGGCGAAGGGGGT	G4CKT445934000004283	-
	ITS1	4607	4631	24	GGGACGGGGCGCCG	G4CKT445934000004607	-
	ITS1	4608	4631	23	CGGGACGGGGCGCCC	G4CKT445934000004608	-
	ITS1	4942	4964	22	GGGAGAGGGCCGGC	G4CKT445934000004942	-
	ITS1	4943	4964	21	AGGGAGAGGGCCGGC	G4CKT445934000004943	-
	ITS1	4951	4970	19	GCTCGGGAGGGAGAG	G4CKT445934000004951	-
	ITS2	6496	6521	25	TGCGGGGGAGGGAAT	G4CKT445934000006496	-
	28S	9790	9814	24	AAGGGGGGGCGGGG	G4CKT445934000009790	-
	28S	9794	9820	26	GGGGAAAGGGGGGG	G4CKT445934000009794	-
	28S	9795	9820	25	CGGGGAAAGGGGGGG	G4CKT445934000009795	-
	28S	9796	9820	24	CGGGGAAAGGGGGG	G4CKT445934000009796	-
	28S	9800	9820	20	GAGCGGGGAAAGGGG	G4CKT445934000009800	-
	28S	9930	9963	33	GGGGCGGAGGGGCAA	G4CKT445934000009930	-
	28S	9931	9963	32	GGGGCGGAGGGGCA	G4CKT445934000009931	-
	28S	9932	9963	31	GGGGGCGGAGGGGC	G4CKT445934000009932	-
	28S	9933	9963	30	GGGGGCGGAGGGGC	G4CKT445934000009933	-
	28S	9942	9971	29	CGGGAGCGGGGGGC	G4CKT445934000009942	-
	28S	9943	9971	28	CGGGAGCGGGGGGG	G4CKT445934000009943	-
	28S	9950	9975	25	GGGCGCCGGGAGCGG	G4CKT445934000009950	-
	28S	9951	9975	24	GGGCGCCGGGAGCG	G4CKT445934000009951	-
	28S	9952	9975	23	GGGGCGCCGGGAGCG	G4CKT445934000009952	-
	28S	9953	9975	22	GGGGCGCCGGGAGC	G4CKT445934000009953	-
	28S	9954	9975	21	AGGGGCGCCGGGAGC	G4CKT445934000009954	-
	28S	9987	10014	27	TCGGGACGGGGGGCG	G4CKT445934000009987	-
	28S	9995	10019	24	GGGCGCTCGGGACG	G4CKT445934000009995	-
	28S	9996	10019	23	AGGGCGCTCGGGACG	G4CKT445934000009996	-
	28S	9997	10019	22	GAGGGCGCTCGGGAC	G4CKT445934000009997	-
	28S	9998	10019	21	GAGGGCGCTCGGGAC	G4CKT445934000009998	-

Supplementary Table 10. Annotation of predicted i-Motif Forming Sequence (iMFS) across rDNA loci of multiple species

Species	rDNA_region	iMFS_start	iMFS_end	IMFS_length	imotif_sequence	predict_score	predict_tranPH	strand
Rhesus macaque	5'ETS	529	561	32	CCCGTTCTTCTCGTCCCGCCCGCGGGGTTTCCC	0.479	6.44	+
	5'ETS	616	642	26	CCCGGCCCGACCTCGCTGTCCCGACCC	0.303	5.95	+
	5'ETS	1317	1340	23	CCCCCTCCCTTCCCCAGGCGTCCC	0.305	5.95	+
	5'ETS	2139	2171	32	CCCGAGTTAGCCCCCTGCGGGTTCCCCGTGCCC	0.375	6.15	+
	5'ETS	2634	2653	19	CCCCCGGCGCCCCCCCTCCC	0.353	6.09	+
	5'ETS	2872	2898	26	CCCGAGTGCGGCCCGGTGGTCCCTCCC	0.311	5.97	+
	5'ETS	2982	3003	21	CCCGGCCCGGGGCCCTCGGCCC	0.404	6.23	+
	5'ETS	3474	3497	23	CCCCGCCCTCCCGCCGCGCCACCC	0.417	6.27	+
	18S	5377	5413	36	CCCTCGGATCGGCCCCGCCGGGGTCGGCCCACGGCCC	0.502	6.51	+
	ITS1	5817	5849	32	CCCTCCCCGCCGCCGGGGCCCGTCGTCGTTCCC	0.461	6.39	+
	ITS1	5984	6009	25	CCCGGTCCCCCCCTCCTCGTCCGCCC	0.491	6.47	+
	ITS1	6136	6161	25	CCCGGACCCCTCCCGTCGCCTCGCCC	0.431	6.31	+
	ITS2	6762	6797	35	CCCGCCGGGGCCCTCCGTCCCCCCAAGCGCAGACCC	0.439	6.33	+
	ITS2	6809	6840	31	CCCTCCCCTTCCGCCGCGCCCGCACCTTTCCC	0.356	6.1	+
	ITS2	7356	7374	18	CCCGCCCCGCGGCCCGCCC	0.348	6.07	+
	ITS2	7652	7676	24	CCCGTCCCGTCCCCGGTCCGTGCCC	0.386	6.18	+
	ITS2	7825	7841	16	CCCGCCCACCCCCGCCC	0.386	6.18	+
	28S	8364	8396	32	CCCGGCGGATCTTTCCCGCCCCCCGTTCCTCCC	0.393	6.2	+
	28S	8399	8417	18	CCCCTCCACCCGCCCTCCC	0.179	5.6	+
	28S	8624	8645	21	CCCCGTCCCGTCCCGTCTTCCC	0.312	5.97	+
	28S	8790	8810	20	CCCCCCTCCCGGCGCCCACCC	0.251	5.8	+
	28S	8906	8925	19	CCCACCCCCTCCCCGCACCC	0.208	5.68	+
	28S	9935	9974	39	CCCCTCCTCCTCCTCCCCCCCCGCCCGCCCCCGGAGCCCC	0.461	6.39	+
	28S	10682	10704	22	CCCCCCCCACGCCCGGGGCACCC	0.367	6.13	+
	28S	10705	10726	21	CCCTCGCGGCCCTCCCCCGCCC	0.368	6.13	+
	28S	10747	10769	22	CCCTCCCCCGCGCCCTCTCTCCC	0.28	5.89	+
	3'ETS	12768	12809	41	CCCTGCTCGGCGCCCCCGCTTCTTCGGTTCCCGCCTCCTCCC	0.459	6.39	+
	5'ETS	109	136	27	CCCACCCAGGCTCCCCGGACACCGTCCC	0.263	5.84	-
	5'ETS	220	246	26	CCCTCCCCCCCCCACTCCTCCACGCCC	0.19	5.63	-
	5'ETS	2097	2120	23	CCCCCCGGGTGCCCGGGCCCCCCC	0.458	6.38	-
	5'ETS	2167	2187	20	CCCCTCCGCCCGACCCTTCCC	0.266	5.84	-
	5'ETS	2329	2354	25	CCCAGCCCCGCTTCGCGCCCCAGCCC	0.239	5.77	-
	18S	3958	3981	23	CCCCGAGAGAACCCCCCCGGGCCC	0.399	6.22	-
	18S	4021	4044	23	CCCCGCCCCCACCCGCGCGGGCCC	0.429	6.3	-
	18S	4297	4315	18	CCCCGACCCGCGCCCTCCC	0.301	5.94	-
	18S	4358	4398	40	CCCCCGAGCCACCTTCCCCGCCGGGCCTTCCCAGCCGTCCC	0.459	6.38	-
	18S	4470	4495	25	CCCCCGCCGACCCCACCCCCGGCCCC	0.349	6.08	-
	18S	4497	4515	18	CCCGCCCACCCCCGCACCC	0.37	6.13	-
	18S	5351	5388	37	CCCCCGCCCACCGACCCCCCGACCCGTCCCCCCCCCCC	0.378	6.16	-
	ITS1	5792	5830	38	CCCCCGACCTCTCCCCGCGCAACCCCTCCGACCTCGCCC	0.438	6.33	-
	ITS1	5837	5862	25	CCCCCCCACCCCCACGACACACGCCC	0.217	5.71	-
	ITS1	5909	5937	28	CCCGGCCCGGGCCAACGAACCCCGCACCC	0.356	6.1	-
	ITS1	6069	6099	30	CCCTCCCTTCTCCGGTCTCCCAGCCGGGCCC	0.324	6.01	-
	ITS1	6100	6117	17	CCCCTCCCCCCCCCACCC	0.036	5.2	-
	ITS1	6209	6248	39	CCCCGGCGGGCCCTGCGAGGCAACCCCCAGCCGCGCACCC	0.453	6.37	-
	5.8S	6634	6661	27	CCCCACAGGCGCCCGGGGGTTCCCGCCC	0.417	6.27	-
	ITS2	6845	6878	33	CCCCTCCCGACGGAACTCCCCCGCCGGCGCGCCC	0.401	6.22	-
	ITS2	7196	7212	16	CCCTCCCCCCCGCGCCC	0.387	6.18	-
	28S	9835	9867	32	CCCGGGGCCCGGCCGAGGCCCGGCTCCGAGCCC	0.499	6.5	-
	28S	9987	10014	27	CCCCGGGCCGGGCCCCACCCCCCGGCCC	0.416	6.27	-
	28S	10950	10987	37	CCCTTGGGCAGCCCACCGAGCAACCCCCTCACGAGCCC	0.405	6.23	-
	28S	11596	11626	30	CCCCACCCGCCAGTCGCCCCTCGTCGGGCCC	0.353	6.09	-
	28S	12470	12493	23	CCCGCCCCTCGCCCGTCGCGGCCC	0.456	6.38	-
Species	rDNA_region	iMFS_start	iMFS_end	IMFS_length	imotif_sequence	predict_score	predict_tranPH	strand
Mouse	5'ETS	1605	1646	41	CCCGAATGGTCCCCTGGAGGGCTCGCCCCCTGACCGCCTCCC	0.499	6.5	+
	5'ETS	1886	1908	22	CCCGGGTCCCCACCCGTCTTCCC	0.416	6.26	+
	5'ETS	2044	2070	26	CCCCCCCCTTCCCGCGGCAGCGTTCCC	0.381	6.17	+
	5'ETS	2443	2487	44	CCCTTGTGAGGACCCCCTTCCGGGAGGGGCCCGTTTCGGCCGCCC	0.476	6.43	+
	5'ETS	3357	3382	25	CCCGCCCGTGAGCCCCTGCCGCACCC	0.341	6.05	+
	5'ETS	3510	3537	27	CCCCCGCCGACCCCCGCCTGCCCGTCCC	0.307	5.96	+
	5'ETS	3636	3666	30	CCCCCACGCCAACCCACCACCCTGCTCTCCC	0.31	5.97	+
	5'ETS	3881	3911	30	CCCGCCCGGCTCACCCCCGGTTTGTCCTCCC	0.405	6.23	+
	18S	5745	5781	36	CCCTCGGATCGGCCCCGCCGGGGTCGGCCCACGGCCC	0.502	6.51	+
	ITS1	6056	6076	20	CCCCTCCCCCTCCCCTCTCCC	0.212	5.69	+
	ITS1	6698	6724	26	CCCGACCCTTTTTTTTTCCTCCCCCCC	0.289	5.91	+
	ITS2	7099	7130	31	CCCCCCAACCCGGGTCGGGCCCTCCGTCTCCC	0.377	6.16	+
	ITS2	7223	7249	26	CCCGCTCCGCCGTTCCCGCCCTCGCCC	0.466	6.4	+
	ITS2	7315	7343	28	CCCGTGCCCCGTGGGAACCCACCGCGCCC	0.399	6.22	+
	ITS2	7662	7687	25	CCCGCGTCCGTCCGTCCCTCCCTCCC	0.446	6.35	+
	ITS2	7888	7920	32	CCCGTTGCGTCCCGGCTTCCCTGGGGGGGACCC	0.369	6.13	+
	ITS2	7972	7992	20	CCCCCGCCGCCCTCCCCTCCC	0.26	5.83	+
	28S	8617	8648	31	CCCGGTGGTCCCGGCGGATCTTTCCCGCTCCC	0.335	6.04	+
	28S	8656	8684	28	CCCGACCCCTCCACCCGCGCGTCGTTCCC	0.356	6.1	+
	28S	9338	9366	28	CCCCGCCCGGGGGCCCGAGGTGGGATCCC	0.311	5.97	+
	28S	10864	10891	27	CCCCCGTCCTTTCCGCCCGGGCCCGCCC	0.385	6.18	+
	28S	10893	10923	30	CCCCTCTTCCCCGCGGGGCCCCGTCGTCCCC	0.423	6.29	+
	28S	12531	12576	45	CCCCGGATAGCCGGGTCCCCGTCCGTCCCCGCTCGGCGGGGTCCCC	0.438	6.33	+
	3'ETS	13290	13312	22	CCCGAGCATCCCCACTCCCGCCC	0.318	5.99	+
	5'ETS	774	805	31	CCCCGGTCCCGACGCCCGGCGGGGGGAGACCC	0.401	6.22	-
	5'ETS	2191	2231	40	CCCGCGGGGAGGGGACCCGCGCCGGCACCCGCCGGGCTCCC	0.368	6.13	-
	5'ETS	2363	2400	37	CCCGCTCCGGAACCCCCGCGGGGTTGGACCCGCCGCCC	0.541	6.61	-
	5'ETS	2594	2619	25	CCCAGCCCCGCTTCGCGCCCCAGCCC	0.239	5.77	-
	5'ETS	3324	3362	38	CCCCCGCCACGCACCCCCGACCCCGAGAGACGCGCACCC	0.376	6.15	-
	18S	4352	4373	21	CCCGGCCCCCACCCCCACGCCC	0.367	6.13	-
	18S	4497	4532	35	CCCTGAGACGCGCCGCCCCCCCCGAGCCACCTTCCC	0.398	6.21	-
	18S	4626	4651	25	CCCCCGCCGACCCCACCCCCGGCCCC	0.349	6.08	-
	18S	5526	5554	28	CCCTCTCCCTCCCCGAACTCGCAACACCC	0.295	5.93	-
	ITS1	5855	5894	39	CCCGCGAGCGGTCGACCCCGCCTCCCCCAGCGCAAGACCC	0.416	6.27	-
	5.8S	6923	6950	27	CCCTACGGGCCCCACCGCCCCCCGACCC	0.339	6.05	-
	ITS2	7363	7390	27	CCCAGACGGACCCAGACCGACCCCACCC	0.252	5.81	-

	28S	9659	9691	32	CCCGCTCTCCCCTCCGTCCCCGGCACAAATCCC	0.445	6.35	-
	28S	9899	9935	36	CCCTGGATAGGCCACCCGTCGGAGCCCGGCGACACCC	0.448	6.35	-
	28S	10726	10750	24	CCCCTCGGCCCCCCCCCAAGACCCC	0.413	6.26	-
	28S	11513	11543	30	CCCGGGAGCCCACCGTCGCCTACCCACACCC	0.268	5.85	-

Species	rDNA_region	iMFS_start	iMFS_end	IMFS_length	imotif_sequence	predict_score	predict_tranPH	strand
Chicken	5'ETS	689	709	20	CCCCCCCACCCCCTTTCTCCC	0.241	5.77	+
	5'ETS	942	971	29	CCCGCCCTGTCGGTGCCCCAGGGCTCGCCC	0.403	6.23	+
	18S	2910	2942	32	CCCATGACCCGCCGGGCAGCTCCCGGGAAACCC	0.3	5.94	+
	ITS1	3722	3741	19	CCCTTCCCTTCCCCGAGCCC	0.365	6.12	+
	ITS1	4269	4299	30	CCCCCGGCCTCACCCCCCACCCCCTTCGCCC	0.388	6.19	+
	ITS1	4608	4630	22	CCCCTCGGCGCCCCGTCCCGCCC	0.389	6.19	+
	ITS1	4943	4964	21	CCCCGCCGGCCCTCTCCCTCCC	0.348	6.07	+
	ITS1	5512	5535	23	CCCGAATCCGTCCCCGTCCCGCCC	0.298	5.94	+
	ITS2	6497	6519	22	CCCGGATTCCCTCCCCCGCACCC	0.331	6.03	+
	28S	7592	7621	29	CCCCGCCTCCGCCTCCCCTCCGTCCCTCCC	0.292	5.92	+
	28S	7865	7899	34	CCCTCCCCCGGAGGGGGCGGCCCCCCGGAGGGCCC	0.491	6.47	+
	28S	9791	9807	16	CCCGCCCCCGCCCCCCC	0.229	5.74	+
	28S	9931	9971	40	CCCCCCTCCTTGCCCCTCCGCCCCCCCGCTCCCGGCGCCCC	0.498	6.49	+
	28S	9973	10006	33	CCCGTCGGCCGTCGTCCCGGCCGCCCCCCGTCCC	0.503	6.51	+
	5'ETS	155	186	31	CCCCCCGCCCCTCTCCCCCTCTTTCCAACCCC	0.437	6.32	-
	5'ETS	1739	1758	19	CCCGGAGACCCCCCCCGCCC	0.365	6.12	-
	18S	2010	2028	18	CCCGCTGACCCCCCCCCCC	0.264	5.84	-
	18S	2101	2126	25	CCCAGCCCCGCTTCGCGCCCCAGCCC	0.239	5.77	-
	18S	3576	3604	28	CCCCGGGCGCCCTGGCGTGGCCCGAGCCC	0.399	6.22	-
	ITS1	3746	3774	28	CCCCCCCGGCGCCCCCCGCGGCCTCGCCC	0.359	6.1	-
	ITS1	3834	3850	16	CCCGACCCCCCCCGCCC	0.389	6.19	-
	ITS1	4099	4126	27	CCCGCCGGCAGCCCTTCCCAGCCGTCCC	0.384	6.17	-
	ITS1	4198	4235	37	CCCCGGCCCGCCCGCCCCCCCTGGGCCCGCCCCGCCCC	0.482	6.45	-
	ITS1	4490	4523	33	CCCAAGCAACCCGACTCCGAGAAGCCCCGGGCCC	0.271	5.86	-
	ITS1	4798	4834	36	CCCGAACGCCGGGCCCCGGGCGAGCCCGCGCTCGCCC	0.486	6.46	-
	ITS1	5735	5769	34	CCCGAAGGCGCTCGGCCCGCGCCCGCGGCCAGCCC	0.464	6.4	-
	ITS1	6142	6174	32	CCCGCGCCCGCGCCGGACCGCCCGACGGCGCCC	0.431	6.31	-
	ITS2	6828	6852	24	CCCCCTCTCCGGCCCGGGCCCGCCC	0.27	5.86	-
	28S	7188	7220	32	CCCGAAGGCCCCGCGGGGGTGCCTCCCCCGCCC	0.461	6.39	-
	28S	7260	7289	29	CCCGGCACCCGCCAGCCGGCCCCGGCGCCC	0.448	6.35	-
	28S	7352	7381	29	CCCGGGGCCCGGGGTTTCCCTCGGCAACCC	0.362	6.11	-
	28S	7533	7553	20	CCCGCGACCCGGCCCCGTCCC	0.362	6.11	-
	28S	7609	7637	28	CCCGCGCCCCGCGCTCGGACCCGCCGCCC	0.452	6.37	-
	28S	10246	10277	31	CCCGATCCCCGGGGCGGCCCCGCCGACCTCCC	0.477	6.44	-
