# Supplementary material for: In silico mapping of non-canonical DNA structures across the human ribosomal DNA locus
Source: G3 (Bethesda). 2025 Dec 10;16(2):jkaf299. doi: 10.1093/g3journal/jkaf299 (PMC12869075; doi:10.1093/g3journal/jkaf299)
Supplement: jkaf299_Supplementary_Data [file jkaf299_supplementary_data.zip › Supplemental_Material_Legends_G3-2025-406410.docx]

**Supplementary Fig 1. Comparison of QmRLFS finder with deep learning-based tools.** Top: Annotation of the human rDNA transcriptional unit (KY962518). Strand specific R-loops predictions for **non-template** (left) and **template** (right) are shown for two approaches: QmRLFS (**a**) and DeepER (**b**).

**Supplementary Fig. 2. Strand-specific RLFS distribution across rDNA repeats on human rDNA repeats.** UCSC genome browser tracks from the T2T CHM13v2.0 assembly are shown for chromosomes 13, 14, 15, 21, and 22. The coordinate-based rDNA region annotation for each chromosome is provided above its respective panel. Within each panel, the rDNA array is marked in black and individual repeat units are shown in green. Strand-specific RLFS coverage tracks are displaced below, with non-template in red and template in blue.

**Supplementary Fig. 3. Strand-specific G4FS distribution across human rDNA repeats.** UCSC genome browser tracks from the T2T CHM13v2.0 assembly are shown for chromosomes 13, 14, 15, 21, and 22. The coordinate-based rDNA region annotation for each chromosome is provided above its respective panel. Within each panel, the rDNA array is marked in black and individual repeat units are shown in green. Strand-specific G4FS coverage tracks are displaced below, with non-template in red and template in blue.

**Supplementary Fig. 4. Strand-specific iMFS distribution across human rDNA repeats.** UCSC genome browser tracks from the T2T CHM13v2.0 assembly are shown for chromosomes 13, 14, 15, 21, and 22. The coordinate-based rDNA region annotation for each chromosome is provided above its respective panel. Within each panel, the rDNA array is marked in black and individual repeat units are shown in green. Strand-specific iMFS coverage tracks are displaced below, with non-template in red and template in blue.

**Supplementary Table 1. Characterization of the human rDNA locus.** The table provides the nucleotide distribution, GC content, and GC skew of the human rDNA region.

**Supplementary Table 2. Mapping and characterization of R-loop forming sequences (RLFSs) in the human rDNA locus.** Columns indicate the rDNA and its associated RLFSs along with genomic coordinates, sequence features, and strand orientation, where plus (+) represent non-template strand and minus (-) represents the template strand.

**Supplementary Table 3. Mapping and characterization of G-quadruplex forming sequences (G4FS) in the human rDNA locus.** For each rDNA region, predicted G4FS are listed with genomic coordinates, sequence features, and strand orientation (“+” = non-template; “-” = template)

**Supplementary Table 4. Mapping and characterization of predicted i-motif–forming sequences (iMFSs) in the human rDNA locus.** For each rDNA region, iMFS are listed with genomic coordinates, sequence features, and strand orientation (“+” = non-template; “-” = template)

**Supplementary Table 5. Predicted counts of G4FS, RIZ, and iMFS across bins of the human rDNA locus.** Columns show the counts per bin for each predicted non-canonical DNA structure sequence, along with binary indicators marking the presence or absence of each motif.

**Supplementary Table 6. Input dataset for POLR1A ChIP-seq and non-canonical structure associations in the human rDNA locus.** Binned data (100 bins across the human rDNA transcription unit) showing POLR1A ChIP-seq signal, predicted non-canonical structure counts (RLFSs, G4FS, iMFSs), normalized values, and strand-specific sign-match indicators used for correlation and comparative analyses.

**Supplementary Table 7. Sequence conservation of rDNA regions across species compared to human.** Percent identity of rDNA regions in Macaca mulatta (Rhesus macaque, KX061890) (Agrawal and Ganley 2016), Mus musculus (Mouse, BK000964) (Grozdanov et al. 2003), and Gallus gallus (Chicken, KT445934) (Dyomin et al. 2016) relative to human (GenBank ID KY962518) (Kim et al. 2018). Individual rDNA regions (5′ETS, 18S, ITS1, 5.8S, ITS2, 28S, and 3′ETS) are listed with their corresponding percent identity values.

**Supplementary Table 8. Annotation of RLFS across rDNA loci of multiple species.** These tables provide a comprehensive annotation of RLFS within the rDNA loci of Macaca mulatta (Rhesus macaque, KX061890) (Agrawal and Ganley 2016), Mus musculus (Mouse, BK000964) (Grozdanov et al. 2003), and Gallus gallus (Chicken, KT445934) (Dyomin et al. 2016). For each rDNA region, genomic coordinates, sequence features, prediction scores, and strand orientation are provided.

**Supplementary Table 9. Annotation of G4FS across rDNA loci of multiple species.** These tables provide a comprehensive annotation of G4FS within the rDNA loci of Macaca mulatta (Rhesus macaque, KX061890) (Agrawal and Ganley 2016), Mus musculus (Mouse, BK000964) (Grozdanov et al. 2003), and Gallus gallus (Chicken, KT445934) (Dyomin et al. 2016). For each rDNA region, genomic coordinates, sequence features, prediction scores, and strand orientation are provided.

**Supplementary Table 10. Annotation of iMFS across rDNA loci of multiple species.** These tables provide a comprehensive annotation of iMFS within the rDNA loci of Macaca mulatta (Rhesus macaque, KX061890) (Agrawal and Ganley 2016), Mus musculus (Mouse, BK000964) (Grozdanov et al. 2003), and Gallus gallus (Chicken, KT445934) (Dyomin et al. 2016). For each rDNA region, genomic coordinates, sequence features, prediction scores, and strand orientation are provided.
